# Discovery and characterization of bromodomain 2–specific inhibitors of BRDT

**DOI:** 10.1073/pnas.2021102118

**Published:** 2021-02-26

**Authors:** Zhifeng Yu, Angela F. Ku, Justin L. Anglin, Rajesh Sharma, Melek Nihan Ucisik, John C. Faver, Feng Li, Pranavanand Nyshadham, Nicholas Simmons, Kiran L. Sharma, Sureshbabu Nagarajan, Kevin Riehle, Gundeep Kaur, Banumathi Sankaran, Marta Storl-Desmond, Stephen S. Palmer, Damian W. Young, Choel Kim, Martin M. Matzuk

**Affiliations:** ^a^Center for Drug Discovery, Department of Pathology & Immunology, Baylor College of Medicine, Houston, TX 77030;; ^b^Department of Pharmacology and Chemical Biology, Baylor College of Medicine, Houston, TX 77030;; ^c^Berkeley Center for Structural Biology, Lawrence Berkeley National Laboratory, Berkeley, CA 94720;; ^d^Verna and Marrs McLean Department of Biochemistry and Molecular Biology, Baylor College of Medicine, Houston, TX 77030

**Keywords:** BET bromodomains, DNA-encoded chemistry, small-molecule inhibitors, male contraceptive

## Abstract

Bromodomain testis (BRDT), a member of the bromodomain and extraterminal (BET) subfamily that includes the cancer targets BRD2, BRD3, and BRD4, is a validated contraceptive target. All BET subfamily members have two tandem bromodomains (BD1 and BD2). Knockout mice lacking BRDT-BD1 or both bromodomains are infertile. Treatment of mice with JQ1, a BET BD1/BD2 nonselective inhibitor with the highest affinity for BRD4, disrupts spermatogenesis and reduces sperm number and motility. To assess the contribution of each BRDT bromodomain, we screened our collection of DNA-encoded chemical libraries for BRDT-BD1 and BRDT-BD2 binders. High-enrichment hits were identified and resynthesized off-DNA and examined for their ability to compete with JQ1 in BRDT and BRD4 bromodomain AlphaScreen assays. These studies identified CDD-1102 as a selective BRDT-BD2 inhibitor with low nanomolar potency and >1,000-fold selectivity over BRDT-BD1. Structure–activity relationship studies of CDD-1102 produced a series of additional BRDT-BD2/BRD4-BD2 selective inhibitors, including CDD-1302, a truncated analog of CDD-1102 with similar activity, and CDD-1349, an analog with sixfold selectivity for BRDT-BD2 versus BRD4-BD2. BROMOscan bromodomain profiling confirmed the great affinity and selectivity of CDD-1102 and CDD-1302 on all BET BD2 versus BD1 with the highest affinity for BRDT-BD2. Cocrystals of BRDT-BD2 with CDD-1102 and CDD-1302 were determined at 2.27 and 1.90 Å resolution, respectively, and revealed BRDT-BD2 specific contacts that explain the high affinity and selectivity of these compounds. These BD2-specific compounds and their binding to BRDT-BD2 are unique compared with recent reports and enable further evaluation of their nonhormonal contraceptive potential in vitro and in vivo.

## Significance

There is no nonhormonal contraceptive pill for men, although hundreds of genes have been identified to play roles during spermatogenesis and fertilization in the male reproductive tract. To address the absence of contraceptive drugs for men, we established a DNA-encoded chemistry technology (DEC-Tec) platform. Our drug discovery campaign on BRDT, a validated spermatogenic-specific contraceptive target, yielded rapid discovery of potent and specific inhibitors of the second bromodomain of BRDT that have unique binding characteristics to BRDT-BD2 relative to BRDT-BD1. Our study emphasizes the robustness and validation of the DEC-Tec platform where the obtained structure–affinity relationship data would allow us to identify specific protein binders immediately without performing exhaustive medicinal chemistry optimization of compounds with potential as male contraceptives.

Although women have had small-molecule contraceptive pill options for over 50 y (1), there is no effective oral contraceptive pill for men. Despite this need, several pharmaceutical firms have terminated their contraceptive development divisions. Thus, the burden is on scientists in academia to identify and validate contraceptives that target novel proteins and structures that are not based on endocrine interventions. Based on proof-of-principle knockout and in vivo chemical biology studies, BRDT is a validated germ cell target for small-molecule contraceptives. BRDT is a tissue-restricted BET subfamily member expressed in pachytene spermatocytes, diplotene spermatocytes, and round spermatids (2). Deletions of either BD1 or both BD1 and BD2 in the mouse BRDT gene result in male sterility (2, 3), indicating that BRDT is a testis-specific target for male contraception. We have shown that JQ1, a selective small-molecule inhibitor of the BET subfamily, induces a complete and reversible contraceptive effect in mice (4). We demonstrated that JQ1 easily crosses the blood–testis boundary (100% bioavailable) to specifically inhibit BRDT function in spermatocytes and spermatids and reduce spermatozoa number and motility; daily intraperitoneal or subcutaneous injections of JQ1 resulted in grossly smaller testes compared with control males. These proof-of-principle small-molecule studies have validated BRDT as a spermatogenic-specific contraceptive target and have shown that JQ1 is a lead compound targeting the male germ cell for reversible contraception.

Despite the above exciting findings, there are two features of JQ1 that limit its contraceptive development. First, the half-life of JQ1 is too short, and it is metabolized rapidly (5). Second, JQ1 binds to the somatic bromodomain family member BRD4 with higher affinity than for BRDT, which influences its side effect profile. Thus, to identify highly potent and selective inhibitors of BRDT for male contraception and to find molecules that bind selectively to the individual bromodomains of BRDT, a DNA-encoded chemistry technology (DEC-Tec) platform was developed and applied for our drug screening campaign. While high-throughput screening (HTS) with million-compound drug libraries is extensively utilized by pharmaceutical companies in early drug development, DNA-encoded chemical libraries are an alternative technology for ligand discovery that addresses the limitations and economic shortcomings of HTS (6). DEC-Tec has been utilized by several pharmaceutical companies and academic institutions to develop potent and specific small-molecule inhibitors to various target proteins, including EPHX2 (7), RIP1 kinase (8), *Mycobacterium tuberculosis* InhA (9), autotaxin (10), BTK (11), the β_2_-adrenergic receptor (12), etc. Using the libraries that we created, our DEC-Tec screening against thrombin (13) and OXA-48 carbapenemase (14) also resulted in nanomolar small-molecule inhibitors for further development.

Recent studies have shown the potential to identify selective inhibitors of BET bromodomains for cancer and immunoinflammation (15–17). BET proteins are believed to be tethered to chromatin predominantly via the first bromodomain based on results with RVX-208 (higher-affinity BD2 inhibitor than BD1 inhibitor), which could not cause significant displacement of BRD4 from chromatin (17). The recent identification of a better BD2-specific inhibitor, GSK046 with >300-fold selectivity over BD1 (16), revealed an important role of the second bromodomain in the recruitment of BET proteins for the induction of gene expression, particularly in the context of immunoinflammation. An improved analog of GSK046 (GSK620) that exhibited similar selective BRD4-BD2 inhibition provided better resolution of immunoinflammatory diseases in their preclinical models (16), suggesting a more effective therapeutic option. Alternatively, several published BD2 selective inhibitors provided contradictory results in various cancer models (15, 16, 18) based on their variable BD2 selectivity and the cellular context of the cancer models used. However, ABBV-744, a small-molecule inhibitor with 95- to 290-fold selectivity for BRD4-BD2, showed similar anticancer efficacy with improved tolerability in prostate cancer xenografts compared to dual-bromodomain BET inhibitors (15). These recent results suggest the potential for BD2-specific inhibitors to avoid the toxicity associated with recent BET inhibitors that have advanced to clinical trials.

In our studies, we demonstrated the discovery and development of an inhibitor series both potent and highly selective for the BD2 domains of BET subfamily members versus BD1 domains. Initial hits from our DEC-Tec screening underwent pharmacophore identification, and high-resolution cocrystal structures between human BRDT-BD2 and two high-affinity analogs were solved. We also describe optimization of microsomal stability properties of the exemplar compounds in vitro and in vivo.

## Results and Discussion

### Production of Recombinant Bromodomain Proteins.

To obtain quality binders from the DEC-Tec selection, it is critical that the target recombinant protein is well folded and highly pure. We chose pET15b and pET28b bacterial expression vectors with an N-terminal polyhistidine tag (6-His; linear) to express and purify human BRDT and BRD4 recombinant bromodomain proteins as previously described (19). His-tagged recombinant proteins eluted from immobilized metal affinity chromatography were further purified with size exclusion gel filtration chromatography to ensure the homogeneity of our preparation. To test whether the purified bromodomain proteins were properly folded, a fluorescence thermal shift stability assay was performed to measure the melting temperature. Further, to determine if the bromodomains were functional, we used an amplified luminescent proximity homogeneous assay (AlphaScreen), using biotinylated JQ1 to confirm the binding and stability of each bromodomain in DEC-Tec selection buffer.

### DEC-Tec Affinity Selection with Bromodomain Proteins.

To identify potent and selective BRDT-BD2 inhibitors for potential male contraception, we established a DEC-Tec platform for which we generated >50 unique chemical libraries cumulatively containing >4.5 billion drug-like compounds. The libraries were combinatorially constructed by sequential cycles of chemical building block attachment to a molecular scaffold and ligation of a corresponding DNA barcode that enabled precise identification in a pool of billions of compounds. Using a split-and-pool strategy, each individual library of ∼100 million compounds was synthesized with a unique core scaffold. Quality assurance of libraries for selections was ensured through optimization and validation of on-DNA chemistry reactions prior to library synthesis and the precision of our DNA barcode reads using Illumina sequencing after library synthesis (13, 20–22). These individual DEC-Tec libraries were subsequently pooled and screened as multibillion compound mixtures against His-tagged recombinant bromodomain proteins. DNA-encoded molecules with affinity to the His-tagged bromodomain protein were separated from the mixture by using nickel nitrilotriacetic magnetic beads that bind polyhistidine; these beads provide a rapid and easy capture of small-scale His-tagged proteins and therefore can minimize the nonspecific interaction between DNA-encoded chemical library materials and the beads, resulting in a clean background in a DNA environment. Illumina next-generation sequencing and informatic analysis allowed the determination of the isolated DNA barcode sequences, and thus, the structures of the enriched drug-like compounds could be identified. Enrichment of binding compounds was measured by a normalized Z-score metric (23). Enrichment was compared between different experimental conditions to identify potent and selective BRDT-BD2 inhibitors.

**Fig. 1.**
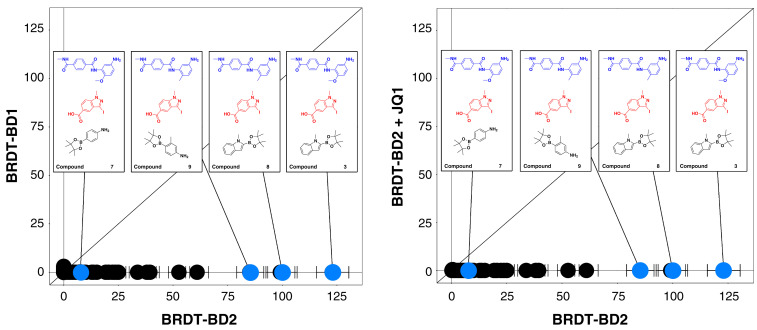
Comparisons of normalized enrichment from parallel DEC-Tec screens against the isolated bromodomains of BRDT. Selected library members are highlighted which showed significant and selective enrichment for BRDT-BD2 compared with BRDT-BD1 (*Left*) and strong dependence on the presence of JQ1 (*Right*). For each highlighted library member, the building blocks for cycles 1, 2, and 3 are shown from top to bottom.

Our selection for BRDT-BD2 binders comprised a pair of affinity selections with a protein concentration of 0.3 µM. One condition included JQ1 as a competitive inhibitor at a concentration of 100 µM, and the parallel condition contained no such inhibitor. To identify bromodomain-specific binders, we also conducted an equivalent pair of affinity selections against BRDT-BD1. Finally, one additional affinity selection was conducted without protein to serve as a no-target control to identify any nonprotein-specific enrichment. Comparison of the normalized enrichment of library members in the data sets highlighted the compound series shown in Fig. 1, which demonstrated strong enrichment for BRDT-BD2 but not BRDT-BD1 and only in the absence of JQ1. Such an enrichment profile is consistent with selective binding to BRDT-BD2 in competition with JQ1. The observed compound series contained a conserved methyl indazole building block in cycle 2, a small number of cycle 1 linker building blocks, and several different aryl boronic acids in cycle 3. Taken together, the strong enrichment and reasonable structure–enrichment relationship suggested a promising chemical series for further investigation. In a parallel selection strategy, BRDT-BD1 inhibitors were also identified with highly selective binding relative to BD2 (e.g., CDD-787; BD1 half-maximal inhibitory concentration (IC_50_) = 2 nM; BD2 IC_50_ = 10,400 nM) that further validates our ability to identify highly selective BET inhibitors using this DEC-Tec platform.

### Validation of BD2 DEC-Tec Selection Hits.

**Scheme 1.**
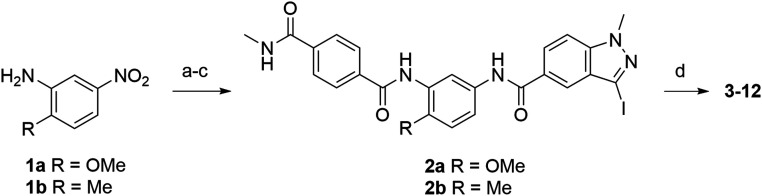
Syntheses of DEC-Tec selection hits **3** to **12**. Reagents and conditions: step a, 4-(methylcarbamoyl)benzoic acid, *O*-(7-azabenzotriazol-1-yl)-*N,N,N',N'*-tetramethyluronium hexafluorophosphate (HATU), *N,N*-diisopropylethylamine (DIEA), *N,N*-dimethylformamide (DMF), room temperature (rt), 16 h; step b, Zn, AcOH, CH_3_OH, rt, 1 h; step c, 3-iodo-1-methyl-1H-indazole-5-carboxylic acid, HATU, DIEA, DMF, rt, 16 h, 27% for **2a** and 35% for **2b** (three steps); step d, boronic acid derivative, [1,1′-bis(diphenylphosphino)ferrocene]dichloropalladium(II) dichloromethane complex (Pd(dppf)Cl_2_·CH_2_Cl_2_) (10 mol %), 1,2-dimethoxyethane (DME)/Na_2_CO_3(sat)_ (vol/vol, 1:1), microwave 85 to 110 °C, 1 h; 24 to 94%.

We first validated the DEC-Tec selection results by resynthesizing hit molecules off-DNA, truncating the DNA barcode linkage to a methyl amide (Table 1). The synthetic pathway of DEC-Tec hits is illustrated in Scheme 1 (see *SI Appendix* for details). Using sequential *O*-(7-azabenzotriazol-1-yl)-*N,N,N',N'*-tetramethyluronium hexafluorophosphate (HATU)-mediated amide coupling, nitro reduction, and another HATU-mediated amide coupling, amines **1a** and **1b** were converted to intermediates **2a** and **2b**, respectively. A subsequent Suzuki−Miyaura cross-coupling reaction furnished the desired products **3** to **12**. These compounds were assayed for inhibition of BRDT-BD2 using an AlphaScreen competition assay with biotinylated JQ1 as the ligand. We confirmed not only that the chemical series significantly inhibited BRDT-BD2 at low nanomolar concentrations but that all compounds showed poor inhibition of BRDT-BD1 with IC_50_ values generally greater than 10 µM, an ∼1,000-fold loss in activity from BRDT-BD2 (Table 1). Thus, our DEC-Tec screening accurately and rapidly guided discovery of potent and selective BRDT-BD2 binding compounds with confirmation through in vitro binding assays. While the correlation between observed sequence counts of hits identified in the DEC-Tec screening and inhibitory activity of compounds resynthesized off-DNA was not one to one due to variance in synthetic yield among library members (24), we were able to separate compounds with low nanomolar affinity (e.g., compounds **3** and **8**) from weaker-binding library members (e.g., compounds **11** and **12**) and identify compounds with high selectivity by using parallel screens.

**Table 1.** Analysis of DEC-Tec hits and predicted library members

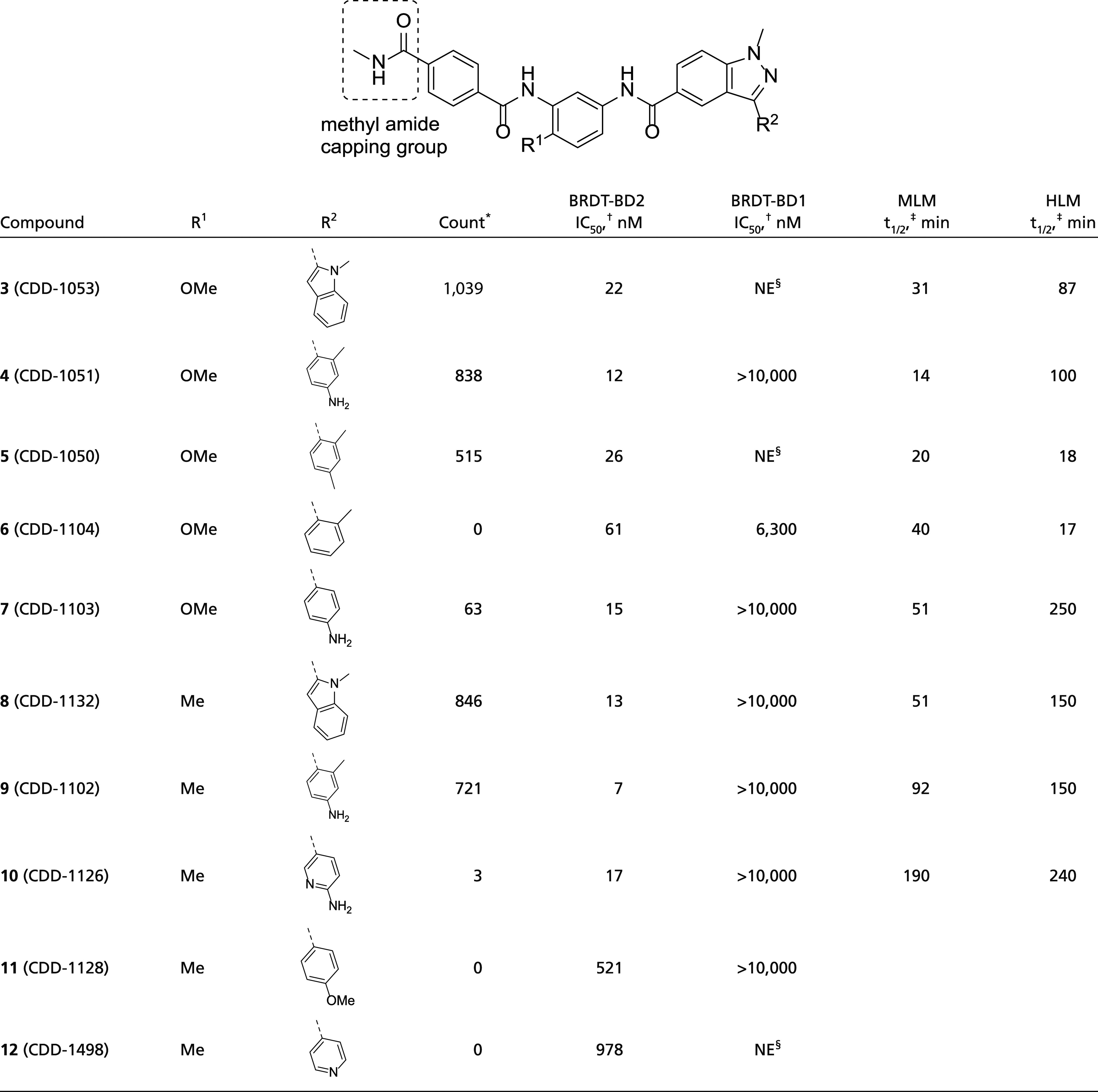

* Observed molecule count in the DEC-Tec screen against BRDT-BD2. A “0” indicates that the compound was theoretically present in the library but was not detected in the sequencing data.

^†^ IC_50_ determined using the AlphaScreen assay protocol.

^‡^ t_1/2_ measured using the liver microsomal stability assay; assay data >60 min are an extrapolated estimate and included for relative determination of the half-life.

^§^ NE: no effect at 10 µM in the AlphaScreen assay.

**Fig. 2.**
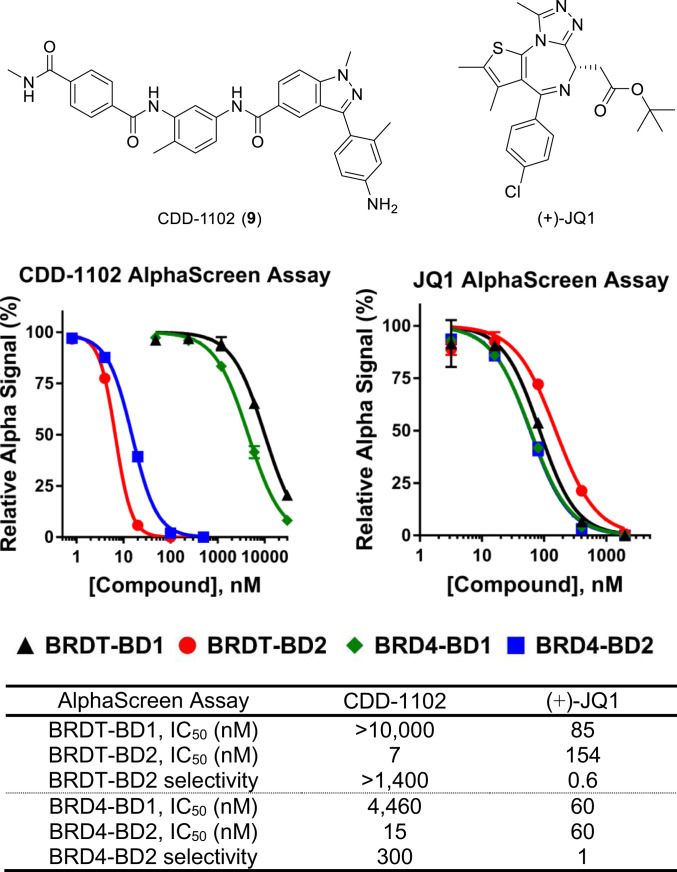
CDD-1102 is a potent and selective inhibitor of the second bromodomain of BRDT and BRD4. Competitive inhibition of human BET bromodomain (BRDT-BD1, BRDT-BD2, BRD4-BD1, and BRD4-BD2) binding to biotinylated JQ1 by CDD-1102 and (+)−JQ1 using proximity detection assays. GraphPad Prism software was used to generate inhibition fitting curves and to determine IC_50_ values.

Among the validated compounds in our initial AlphaScreen assay, compound **9** (herein called CDD-1102; Table 1 and Fig. 2) emerged as an optimal BRDT-BD2 inhibitor with 1) single-digit IC_50_, 2) 22-fold higher potency than JQ1, 3) 1,400-fold selectivity over BRDT-BD1, 4) high metabolic stability in human liver microsomes (HLMs), and 5) greater stability in mouse liver microsomes (MLMs) over other hits. To confirm the direct binding between CDD-1102 and BRDT-BD2, we performed biophysical analysis using a fluorescence thermal shift stability assay (*SI Appendix*, Fig. S2). Upon protein binding, CDD-1102 showed a significant stabilization of the second bromodomain but not the first bromodomain of both BRDT and BRD4. Moreover, the increase in melting temperatures of the second bromodomains by CDD-1102 is more than that of JQ1, indicating a higher protein binding affinity of our compound consistent with the AlphaScreen results.

The binding of CDD-1102 to BET bromodomains was also studied using a NanoBRET target engagement assay which measures compound binding at select target proteins within intact cells. The inhibition of CDD-1102 on tracer binding was demonstrated to be potent and selective in NanoLuc-BET second bromodomain fusion proteins in transiently transfected HEK293 cells (*SI Appendix*, Fig. S3).

### Exploration of Structure–Activity Relationships.

**Scheme 2.**
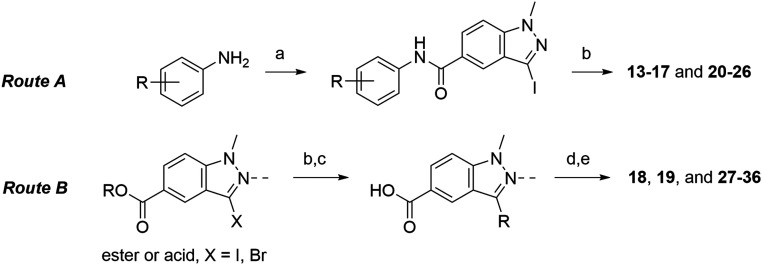
General synthetic routes to analogs **13** to **36**. Reagents and conditions: step a, 3-iodo-1-methyl-1*H*-indazole-5-carboxylic acid, HATU, DIEA, DMF, rt, 16 h, 71 to 95%; step b, boronic acid derivative, Pd(dppf)Cl_2_·CH_2_Cl_2_ (10 mol%), DME/Na_2_CO_3(sat)_ (vol/vol, 1:1), microwave 90 to 110 °C, 1 h, 29 to 95%; step c, for ester only: LiOH·H_2_O, THF, H_2_O, 45 °C, 16 h; step d, RNH_2_, HATU, DIEA, DMF, rt, 16 h; step e, for *N*-Boc deprotection only: 4 N HCl, 1,4-dioxane, rt, 1 h, 14 to 91% (three steps).

Because BRDT is expressed in adult male germ cells in the testes, the blood–testis barrier (BTB), a lining of Sertoli cells impermeable to many small molecules, is a serious obstacle for drug development (25). Based on reports describing similarities between the BTB and blood–brain barrier (BBB) in terms of small-molecule permeability and drug transporters (26, 27), we reasoned that using BBB permeability guidelines, such as molecular weight ≤ 400 g/mol, logP ≤ 5, hydrogen bond donor ≤ 3, and hydrogen bond acceptor ≤ 7, would be a useful goal for hit optimization (28, 29). As CDD-1102 had a molecular weight of 537 g/mol, calculated partition coefficient (cLogP) of 3.1, and five hydrogen bond donors, we explored truncated analogs of the hit series with lower molecular weight and a reduced number of hydrogen bond donors while maintaining cLogP. All analogs were prepared by following a general synthetic pathway (route A or B) in Scheme 2 (see *SI Appendix* for details). In route A, commercially available or synthesized amines were converted to amides, and a subsequent Suzuki coupling with a variety of boronic acid derivatives furnished analogs **13** to **17** and **20** to **26**. In route B, the Suzuki coupling reaction was carried out first. Subsequent saponification for the ester followed by amide coupling provided compounds **18**, **19**, and **28** to **36**. The same procedure with additional acid treatment in the last step produced analog **27**. These synthesized compounds are summarized into three groups (Tables 2–4) based on the type of heterocycles attached to the indazole core.

**Table 2.** Structures and activities of aminopyridine analogs 13 to 19

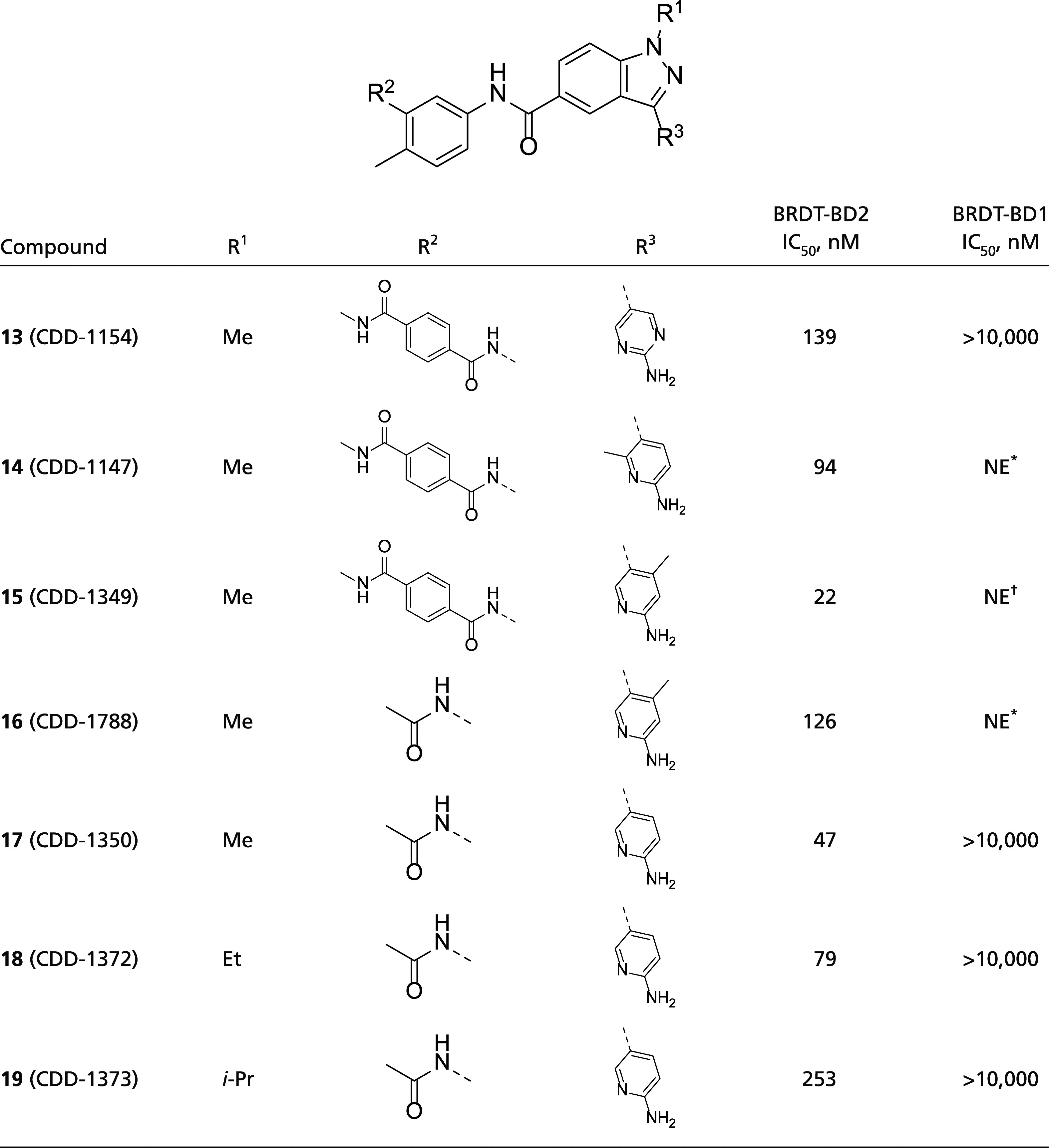

* NE: no effect at 10 µM in the AlphaScreen assay.

^†^ NE: no effect at 20 µM in the AlphaScreen assay.

**Table 3.** Structures and activities of CDD-1102 analogs 20 to 27

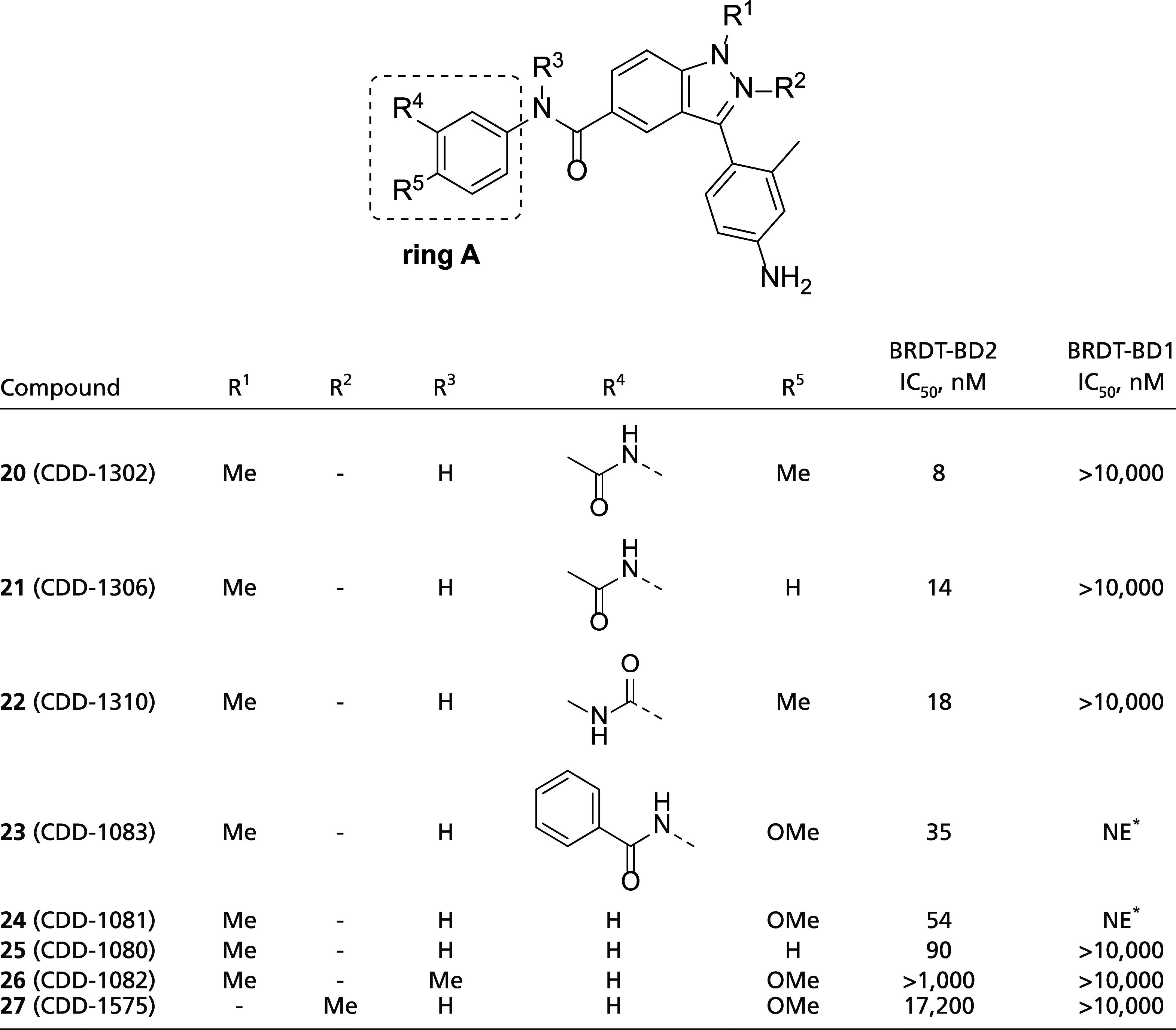

* NE: no effect at 10 µM in the AlphaScreen assay.

**Table 4.** Exploration of functionalized ring A among indole derivatives 28 to 36

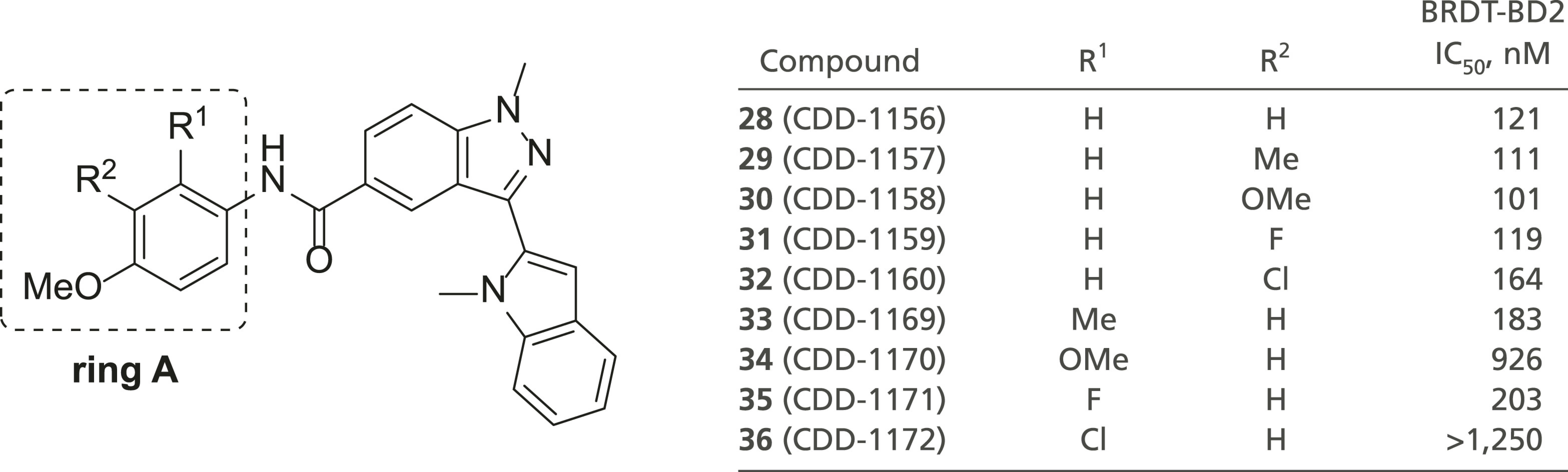

Aminopyridine **10**, despite a slightly lower affinity than CDD-1102, showed high metabolic stability in mouse and human liver microsomes and mitigation of potential Ames positive behavior in CDD-1102 based on the exposed aniline. The aminopyrimidine analog (compound **13**) and methyl substitution of the aminopyridine to mimic the 2-methyl-4-aminophenyl ring in CDD-1102 (compounds **14** and **15**) were attempts to improve affinity, but all displayed lower affinity than compound **10** (Table 2). On the indazole core, methyl homologation led to loss in potency (compounds **17** to **19**), and switching the methyl from N1 to N2 caused a dramatic loss of activity (Table 3, compounds **24** and **27**). It is noteworthy that loss of potency in compound **26** suggested the amide adjacent to the indazole is critical.

A truncation series (compounds **4**, **23** to **25**) from the DNA attachment point toward ring A demonstrated progressive loss of activity while still retaining IC_50_ < 100 nM (Table 3). Interestingly, truncation from CDD-1102 to compound **20** (herein called CDD-1302) showed no loss in activity despite the loss of >100 g/mol molecular weight. From CDD-1302, the loss of the R^3^ methyl (compound **21**) or reversing the amide (compound **22**) imparted minimal effects on potency. Based on these findings, we investigated a series of simple substituents off ring A to assess whether other groups could substitute for the amide and retain activity while removing a hydrogen bond donor (Table 4). Relative to simplified comparator **28**, methyl, methoxy, and fluoro substituents (compounds **29** to **31**) at R^1^ showed equivalent activity, while Cl at R^1^ and all examined R^2^ substituents showed a loss in activity.

**Fig. 3.**
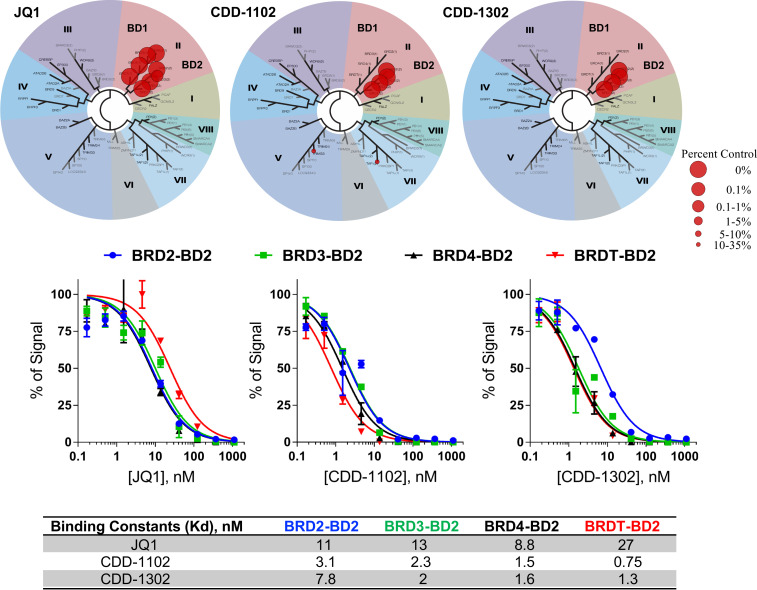
BROMOscan bromodomain profiling of JQ1, CDD-1102, and CDD-1302 on various bromodomains. Phylogenetic tree of bromodomains demonstrating preferential compound binding of CDD-1102 and CDD-1302 for the BET subfamily BD2 domains using the BROMOscan bromodomain competition binding assay performed by the Eurofins DiscoverX Corporation (*Top*). BromoKdELECT dose–response curves and the calculated binding constants (*K*_d_) confirmed that CDD-1102 and CDD-1302 are strong binders to the BET subfamily BD2 domains, with the highest affinity for BRDT-BD2 (*Middle* and *Bottom*). JQ1 binding to both BET bromodomains served as a control in these assays.

Selected potent compounds with IC_50_ < 50 nM were further studied for BRD4 activity and microsomal stability (summarized in Table 5). All tested analogs demonstrated both high BRD4-BD2 versus BRD4-BD1 selectivity and BRDT-BD2 versus BRDT-BD1 selectivity, although intrabromodomain selectivity was greater for BRDT than BRD4. Interestingly, aminopyridine analog **15** (herein called CDD-1349) was observed to have enhanced BRDT-BD2 versus BRD4-BD2 selectivity over other tested analogs. High human microsomal stability was observed for all tested derivatives, while high mouse microsomal stability was measured for compounds **10**, **15**, **17**, and **22**. As CDD-1302 displayed a good balance of microsomal stability and activity, it was selected as an optimized hit for further analysis. BROMOscan bromodomain competition binding assays further confirmed the affinity and the selectivity of these compounds for the second bromodomain of BET subfamily members (Fig. 3). While CDD-1102 demonstrated very weak binding to two bromodomains outside of the BET subfamily, CDD-1302 showed potent and selective binding to only the second bromodomains within the BET subfamily. Both of these CDD compounds have the highest affinity for BRDT-BD2 among all of the bromodomains (Fig. 3). BromoKdELECT dose–response curves show that CDD-1102 and CDD-1302 are more than 20-fold more potent than JQ1 at binding BRDT-BD2 in this assay (Fig. 3), approximating the AlphaScreen findings presented in Table 5.

**Table 5.** Summary and comparison of BRDT and BRD4 activities for JQ1 and potent compounds from DEC-Tec hits and analogs

Compound	BRDT IC_50_, nM		BRDT-BD2 vs. BRDT-BD1	BRD4 IC_50_, nM		BRDT-BD2 vs. BRD4-BD2	BRD4-BD2 vs. BRD4-BD1	t_1/2_,* min	
	BD2	BD1		BD2	BD1			MLM	HLM
**JQ1** (control)	154	85	0.6	60	60	0.4	1	15	6
**8** (CDD-1132)	13	>10,000^†^	>750	31	26,200	2.4	850	51	150
**9** (CDD-1102)	7	10,100	1,440	15	4,460	2.1	300	92	150
**10** (CDD-1126)	17	>10,000^‡^	>600	46	>10,000^‡^	2.7	>210	190	240
**15** (CDD-1349)	22	NE^§^	∼1,000	140	NE^§^	6.4	>140	150	220
**17** (CDD-1350)	47	36,200	770	51	25,100	1.1	490	200	490
**20** (CDD-1302)	8	15,800	2,000	12	10,400	1.5	860	72	370
**21** (CDD-1306)	14	19,700	1,400	21	14,100	1.5	670	9	180
**22** (CDD-1310)	18	10,700	590	20	10,900	1.1	540	150	460

* t_1/2_ measured using liver microsomal stability assay; assay data >60 min are an extrapolated estimate and included for relative determination of the half-life.

^†^ Inhibition is 11% at 10 µM for BRDT-BD1.

^‡^ Inhibition is 16% at 10 µM for BRDT-BD1 and 20% at 10 µM for BRD4-BD1.

^§^ NE: no effect at 20 µM in the AlphaScreen assay.

### Cocrystal Structures of CDD-1102 and CDD-1302 with BRDT-BD2.

**Fig. 4.**
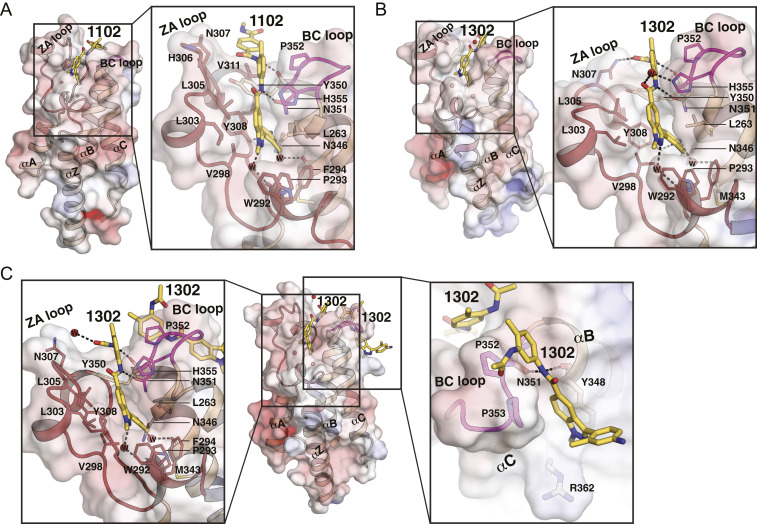
Detailed BRDT-BD2 inhibitor interactions. (*A*) BRDT-BD2/CDD-1102 complex. The electrostatic potential surface is shown with the secondary structure on the *Left* and a zoom-in view of detailed interactions on the *Right*. The ZA loop is colored in red, the BC loop is in magenta, and the rest are in wheat. CDD-1102 is shown as sticks and colored by atom type, with carbon in yellow, nitrogen in blue, and oxygen in red. The ordered waters are shown by red spheres. The hydrogen bonds are shown by dotted lines. (*B*) BRDT-BD2/CDD-1302 complex with one CDD-1302 bound. The electrostatic potential surface is shown with the secondary structure on the *Left* and a zoom-in view of detailed interactions on the *Right*. CDD-1302 is colored by atom type using the same color scheme as in *A*. (*C*) BRDT-BD2/CDD-1302 complex with two CDD-1302 bound. The electrostatic potential surface is shown with the secondary structure in the cartoon in the *Center*. Zoom-in views of detailed interactions for CDD-1302 bound at the KAc pocket are shown on the *Left*, and the second site is on the *Right*.

To understand the structural basis of high affinity and selectivity, we solved crystal structures of BRDT-BD2 bound with CDD-1102 (Protein Data Bank [PDB] ID code 7L9A) and CDD-1302 (PDB ID code 7L99) at 2.27 and 1.90 Å resolution, respectively (Fig. 4 and *SI Appendix*, Table S2). The BRDT-BD2/CDD-1102 crystal contained two molecules per asymmetric unit, whereas the BRDT-BD2/CDD-1302 crystal contained four (*SI Appendix*, Figs. S4 and S5). The overall structure of two molecules in the BRDT-BD2/CDD-1102 crystal was similar, with one CDD-1102 bound at each acetylated lysine (KAc) binding pocket (showing an rmsd of 0.25 Å between shared 97 CA atoms) (*SI Appendix*, Fig. S4*B*). The overall structure of four molecules in the BRDT-BD2/CDD-1302 crystal was nearly identical except for a short segment of the ZA loop (residues 305 to 308). In two of the BRDT-BD2/CDD-1302 complexes in the crystal (molecules A and B), an arginine residue (R341) from the neighboring molecule in the crystal interacted with N307 and the bound CDD-1302 through hydrogen bonds (*SI Appendix*, Fig. S5*A*). This caused a segment of the ZA loop to move away slightly from the core (*SI Appendix*, Fig. S5*B*). Last, the other two molecules showed an additional CDD-1302 bound at a previously unobserved site near the BC loop (*SI Appendix*, Fig. S5*A*). The additional CDD-1302 molecules bound at molecules C and D showed several contacts with other protein molecules in the asymmetric unit mediated through Jeffamine ED-2000 and ordered waters. These contacts suggested that the different ZA loop conformations and the second CDD-1302 binding were caused by crystal packing.

CDD-1102 binds to the extended pocket consisting of the KAc binding pocket, ZA channel, WPF shelf, and a channel formed between ZA and BC loops (Fig. 4*A*) (30, 31). Specifically, the aniline nitrogen interacts with the side chain of Y308 in the ZA loop, whereas the two internal amide nitrogens form hydrogen bonds with the backbone carbonyl of Y350 and the side chain of N351 at the BC loop. The aniline ring binds deep into a hydrophobic pocket consisting of nonpolar residues from the ZA loop (V298) and B and C helices (Y308 and C347). The indazole ring docks to the WPF shelf (W292, P293, F294), the ZA (V298, L303, and L305) and the BC (H355) loops, and the C helix (V357). The following *m*-phenylenediamine ring is partially accessible to solvent and docks to the channel formed between the ZA (H306 and V311) and BC (Y350, P352, and H355) loops though hydrophobic contacts. The terminal amide does not provide any hydrogen bonding interactions, and the terephthalamide ring docks to a shallow hydrophobic surface consisting of N307 and V311 side chains at the ZA loop (Fig. 4*A*). Two ordered water molecules mediate BRDT-BD2/CDD-1102 interactions at the ZA channel and the KAc binding pocket. The backbone carbonyl oxygen atoms of P293 and M343 form hydrogen bonds with the aniline and indazole nitrogens through ordered waters.

**Fig. 5.**
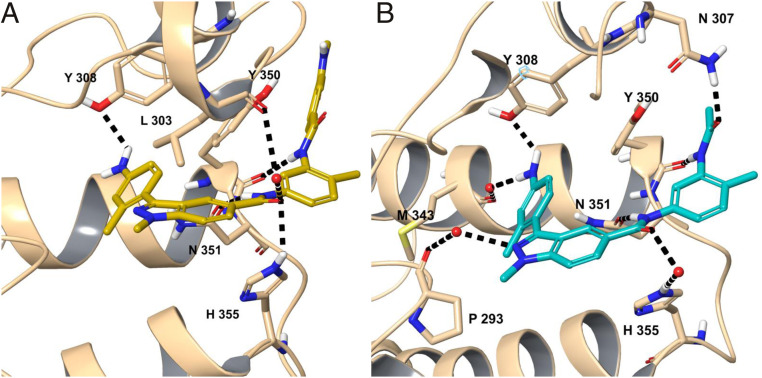
The H bonding interactions between the KAc binding site residues of BRDT-BD2 and the small-molecule inhibitors (*A*) CDD-1102 (yellow) and (*B*) CDD-1302 (cyan). To better show these, the cocrystal structures of CDD-1102 and CDD-1302 were subjected to a restrained geometry optimization after addition of hydrogens, estimation of protonation states, and optimization of hydrogen bond networks via the Schrodinger suite of programs. The residues interacting with the ligand are shown in stick representation and labeled. The water molecules are shown as red dots. The black dashed lines represent H bonds. The point of view angle was adjusted to display the H bonding interactions as clearly as possible.

**Fig. 6.**
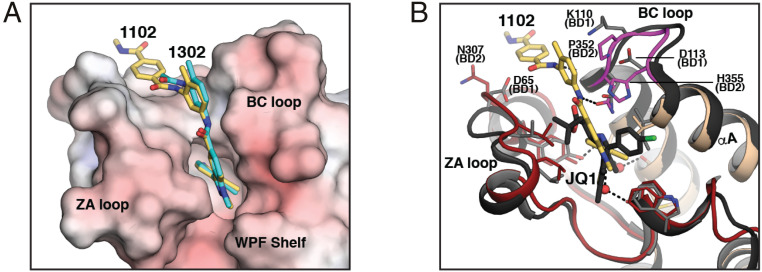
Structure comparisons. (*A*) Superposition of the BRDT-BD2/CDD-1102 and BRDT-BD2/CDD-1302 complexes. The BD2/CDD-1102 complex is superimposed with the BD2/CDD-1302 complex. Only the electrostatic potential surface belonging to the BD2/CDD-1302 complex is shown. CDD-1102 and CDD-1302 are colored using the same color scheme as in Fig. 4 except for carbon atoms of CDD-1302 (cyan). (*B*) Superposition with the BRDT-BD1/JQ1 complex. The BRDT-BD2/CDD-1102 complex (PDB ID code 7L9A) is superimposed with the BRDT-BD1/JQ1 complex (PDB ID code 4FLP). For the BRDT-BD2/CDD-1102 complex, the ZA loop is colored in red, the BC loop is in magenta, and the rest is in wheat. BRDT-BD1 is colored in black. Key interacting residues are shown as sticks. BD2-specific contact residues and corresponding BD1 residues are labeled. JQ1 is colored by atom type using the same color scheme except for carbon (black), chloride (green), and sulfur (yellow).

The BRDT-BD2/CDD-1302 complex revealed that CDD-1302 binds similarly to CDD-1102 excluding the contacts with the missing methyl benzamide ring (Figs. 4*B*, 5, and 6*A*). As mentioned, a segment of the ZA loop containing H306 and N307 moves away slightly (*SI Appendix*, Fig. S5*B*), and this allows the side chain of N307 to move closer to CDD-1302 for a new hydrogen bond with the terminal amide oxygen (Fig. 4*B*). Also unique to the BRDT-BD2/CDD-1302 complex is a water-mediated interaction between the side chain of H355 and the internal amide oxygen. To show the hydrogen bonds formed by CDD-1102 and CDD-1302 to the binding pocket more clearly, the cocrystal structures of CDD-1102 and CDD-1302 were subjected to a restrained geometry optimization after the addition of hydrogens, estimation of protonation states, and optimization of hydrogen bond networks via the Schrodinger program suite (32) (Fig. 5).

Despite having two CDD-1302 bound, molecules C and D show structures highly similar to the BRDT-BD2/CDD-1102 complex including the ZA loop. As for the CDD-1302 bound at the KAc pocket, their interactions are nearly identical to those seen in the BRDT-BD2/CDD-1102 complex, excluding the contacts with the missing terephthalamide ring and one hydrogen bond (Figs. 4*C* and 5). The side chain of Y308 moves away slightly and no longer forms a hydrogen bond with the aniline nitrogen. The secondary CDD-1302 molecule binds to the back side of the BC loop through hydrogen bonds and stacking interactions. Backbone carbonyl oxygens of Y348 and N351 form hydrogen bonds with the amide nitrogens. Additionally, two consecutive prolines, P352 and P353, are perfectly positioned to interact with the *m*-phenylenediamine and indazole rings through hydrophobic interactions (Fig. 4*C*). The aniline ring is fully exposed to solvent, showing no interaction with BD2.

Comparison of the BRDT-BD2/CDD-1102 complex with the BRDT-BD1/JQ1 complex explains the high BRDT-BD2 versus BRDT-BD1 selectivity observed for CDD-1102 and CDD-1302 (Fig. 6*B*) (4). As shown in Fig. 4, the indazole and *m*-phenylenediamine rings make extensive hydrophobic contacts with P352 and H355 at the BC loop as well as a water-mediated hydrogen bond with H355. These conserved BRDT/BRD4-BD2 domain residues are replaced with Lys and Asp (K110 and D113) in BD1, significantly different residues in terms of hydrophobicity and electrostatic charge; we hypothesize that these changes are responsible for the observed selectivity for BRDT-BD2 versus BDRT-BD1 (Fig. 6*B*). A similar BD2 selectivity mechanism was observed for a recently reported BRDT-BD2 inhibitor, iBET-BD2 (16). The relatively compact JQ1 shows no interaction with any of the aforementioned residues, which may explain its low BRDT-BD2 versus BRDT-BD1 selectivity. The BRDT-BD2/CDD-1302 complex shows additional BD2-specific contacts that may contribute to its selectivity. As mentioned, the internal amide nitrogen interacts with N351 through a direct hydrogen bond, whereas the internal amide oxygen interacts with the BD2-specific H355 through water (Figs. 4*B* and 5).

Our structures revealed reorganization of solvent molecules upon inhibitor binding. Unseen in previously published structures, both CDD-1102 and CDD-1302 bind deep into the KAc pocket, replacing a conserved solvent molecule (4, 30). This water molecule bridges a conserved Tyr (Y308 in BRDT-BD2) to the carbonyl oxygen of KAc on the histone or the bound small-molecule inhibitor acting as a second handle in binding. The same carbonyl oxygen of KAc also interacts with the conserved N351, further highlighting its crucial role. Our computationally modeled binding poses prior to the production of cocrystals retained this water, whose replacement in the cocrystals surprised us (*SI Appendix*, Fig. S6). To date, there has been only one other BET inhibitor documented to replace this conserved ZA channel water, albeit with only micromolar affinity and selective for BRD4-BD1 over BRD4-BD2 (33). Last, the structures show a conserved water molecule in the ZA channel that bridges the indazole nitrogen and the backbone carbonyl group of P293 (Fig. 5). P293 belongs to the conserved WPF shelf motif of BET proteins; hence, this water-mediated interaction of compound **27** is significant. P293’s contribution to binding is also supported by the inactivity of compound **27**, which possesses a 2-methyl-2H-indazole blocking water-mediated H bonding to the WPF shelf.

In summary, our drug discovery campaign to identify BRDT selective inhibitors with DNA-encoded chemical libraries identified a chemical series with potent and selective binding to the second bromodomain of BET family proteins. After off-DNA hit confirmation, the potency and the selectivity of these compounds were validated in multiple in vitro biochemical and biophysical binding assays. CDD-1102 was identified as a potent, BD2 versus BD1 selective compound with enhanced metabolic stability over other compounds. Optimization studies led to identification of CDD-1302 with similar potency, selectivity, and metabolic stability with significantly reduced molecular weight and less hydrogen bond donors. Cocrystal structures of BRDT-BD2 with CDD-1102 and CDD-1302 were solved, and binding modes were determined to be similar to one another. Our medicinal chemistry optimization strategy also allowed us to identify CDD-1349 as a molecule with greater than sixfold higher affinity for BRDT-BD2 versus BRD4-BD2, suggesting that this molecule could have a more potent effect in the testis versus somatic cells. Cumulatively, our results have identified structurally unique BET-BD2-specific compounds with both strong potency and improved selectivity compared with recent reports, enabling us to perform additional in vitro and in vivo tests of contraceptive efficacy in the future.

## Materials and Methods

Protocols of recombinant protein production, DEC-Tec affinity selection, assay validation, metabolic stability assay, BROMOscan, cocrystallization, and procedures for and characterization of synthetic compounds are available in *SI Appendix*.

## Supplementary Material

Supplementary File

## Data Availability

The paper and *SI Appendix* contain all datasets generated during this study.
