# Supplementary material for: Discovery and characterization of bromodomain 2–specific inhibitors of BRDT
Source: Proc Natl Acad Sci U S A. 2021 Feb 26;118(9):e2021102118. doi: 10.1073/pnas.2021102118 (PMC7936271; doi:10.1073/pnas.2021102118)
Supplement: Supplementary File [file pnas.2021102118.sapp.pdf]

PNAS
www.pnas.org

Supplementary Information for

Discovery and characterization of bromodomain 2-specific inhibitors of BRDT

Zhifeng Yu[1,*], Angela F. Ku[1,*], Justin L. Anglin[1,*], Rajesh Sharma[2,*], Melek Nihan Ucisik[1], John C. Faver[1], Feng Li[1,2], Pranavanand Nyshadham[1], Nicholas Simmons[1], Kiran L. Sharma[1], Sureshbabu Nagarajan[1], Kevin Riehle[1], Gundeep Kaur[2], Banumathi Sankaran[4], Marta Storl-Desmond[1], Stephen S. Palmer[1], Damian W. Young[1,2,3], Choel Kim[1,2,3,#], and Martin M. Matzuk[1,2,#]

[1]Center for Drug Discovery, Department of Pathology & Immunology, Baylor College of Medicine, Houston, Texas 77030, United States

[2]Department of Pharmacology and Chemical Biology, Baylor College of Medicine, Houston, Texas 77030, United States of America

[3]Verna and Marrs McLean Department of Biochemistry and Molecular Biology, Baylor College of Medicine, Houston, Texas 77030, United States of America

[4]Berkeley Center for Structural Biology, Lawrence Berkeley National Laboratory, Berkeley, California 94720, United States of America.

*, equal author; #, communicating authors, mmatzuk@bcm.edu or ckim@bcm.edu

Table of Contents

1. DEC-Tec Affinity Selection
1a) Production of recombinant bromodomain proteins……………………………..…..……….. S3
1b) DEC-Tec affinity selection with bromodomain proteins…... S3
2. Assays
2a) Bromodomain proximity assay………….……….……………………………….…..........S5
2b) Thermal shift assay……….……….……………………………………….........................S5
2c) NanoBRET target engagement intracellular BET bromodomain assay……….….................S5
2d) Metabolic stability assay in liver microsomes…………………….……….…..................... S5
2e) BROMOscan bromodomain profiling……………………………………………….S5
3. Crystallography……………….…….………………………………………….…......................... S6
4. Syntheses of Compounds
4a) Materials and instrumentation………………….………………………….………… S7
4b) General procedure for the Suzuki-Miyaura reaction………………………………………… S7
4c) Synthesis of intermediate **2a**……………………………………………………………….S7
4d) Synthesis of intermediate **2b**……………………………………………………………… S8
4e) Syntheses of DEC-Tec hits **3−12** and compounds **13−15**……………………………….S9
4f) Syntheses of compounds **16**, **17** and **20**………………………………………………………S14
4g) Synthesis of compound **18**……………………………………………………………..S16
4h) Synthesis of compound **19**……………………………………………………………..S17
4i) Synthesis of compound **21**…………………………………………………………...S18
4j) Synthesis of compound **22**…………………………………………………………...S18
4k) Synthesis of compound **23**……………………………………………………………..S19
4l) Synthesis of compound **24**…………………………………………………………...S20
4m) Synthesis of compound **25**……………………………………………………………S21
4n) Synthesis of compound **26**……………………………………………………………..S22
4o) Synthesis of compound **27**……………………………………………………………..S22
4p) Syntheses of compounds **28−36**………………………………………………………..S23
4q) ^{1}H and ^{13}C NMR spectra of CDD-1102 (**9**), CDD-1349 (**15**) and CDD-1302 (**20**)…………S28
5. Reference…..……………….…….……………………………………….………..............................S31
6. Supplementary Figures and Tables
6a) Figure S1……………………………………………………………………………….S32
6b) Figure S2……………………………………………………………………………….S33
6c) Figure S3……………………………………………………………………………….S34
6d) Figure S4……………………………………………………………………………….S35
6e) Figure S5……………………………………………………………………………….S36
6f) Figure S6……………………………………………………………………………….S37
6g) Table S1……………………………………………………………………………..S38
6h) Table S2……………………………………………………………………………..S39

1. DEC-Tec Affinity Selection

1a) Production of recombinant bromodomain proteins. cDNAs encoding human BRDT and BRD4 bromodomains were either synthesized by Genewiz with codon optimization for bacterial expression or reverse-transcribed from mRNA isolated from the human testes. De-identified human testis tissue was obtained from the Human Tissue Acquisition and Pathology core service (Baylor college of Medicine, USA). Informed consent of these human tissues was obtained. The polymerase chain reaction (PCR) using Phusion high-fidelity DNA polymerase (ThermoFisher Scientific, USA) was applied to amplify the bromodomain regions of BRDT and BRD4 from these cDNA templates. Sequence boundaries for individual recombinant bromodomains to be produced were specified as follows: BRDT-BD1, NP_001717.3 amino acid 21−137; BRDT-BD2, NP_001717.3 amino acid 269−380; BRD4, NP_055114.1 amino acid 44−168; and BRD4-BD2, NP_055114.1 amino acid 333−460. PCR products were purified using Zymo DNA clean & concentrator kit (Zymo Research, USA) and digested with restriction enzymes to generate sticky ends for further subcloning into pET15b or pET28b bacterial expression vectors (Addgene, USA) with an *N*-terminal polyhistidine tag. The constructs were transformed into TOP10 competent cells to yield the final plasmid DNA and sequence alignment was validated by Sanger sequencing.

To produce the individual recombinant bromodomain proteins (Table S1), colonies from freshly transformed competent E. coli NiCo21 (DE3) cells were cultured overnight at 37 °C in Luria-Bertani medium (LB-broth, Merck) with 50 μg/mL kanamycin. These cells were diluted 1:50 in fresh medium and cultured at 37 °C until the optical density reached about 0.5 (OD600). The temperature of the culture was then decreased to 18 °C. Once the system equilibrated at this temperature, OD600 was re-checked to assure not exceeding 0.8, and protein expression was induced overnight by the addition of 0.2 mM isopropyl-β-Dthiogalactopyranoside (IPTG). The bacterial cells were harvested by centrifugation at 4,000 rpm for 45 min at 4 °C. The pellet of cells expressing His-tagged bromodomain proteins was resuspended in lysis buffer (20 mL buffer/cells from 250 ml culture) containing 50 mM Hepes pH 7.5, 500 mM NaCl, 1 mM TCEP (tris(2-carboxyethyl)phosphinehydrochloride)) and 10 mM imidazole, supplemented with Roche cOmplete™ Protease Inhibitor Cocktail. The well-resuspended cells were lysed using French Press in a pre-chilled cell. The lysate was cleared by centrifugation at 16,000 g for 1 h at 4 °C on a Beckman Coulter ultracentrifuge and incubated with activated Talon cobalt resins (Takara Bio, USA) overnight at 4 °C with end over end rotation. Talon cobalt resins with protein bound were packed onto a 10 mL glass column to enable a gravity-flow purification of His-tagged bromodomain proteins. The column was washed once with 30 mL of lysis buffer then twice with 10 mL of lysis buffer containing 30 mM imidazole. The protein was eluted using a step elution of imidazole in lysis buffer (50, 100, 150 and 250 mM imidazole in 50 mM Hepes, pH 7.5, 500 mM NaCl). All fractions were collected and monitored by SDS-polyacrylamide gel electrophoresis (Invitrogen NuPAGE™ 4 to 12%, Bis-Tris gradient gel). Fractions with enriched His-tagged bromodomain proteins were pooled and further purified with size exclusion chromatography on a superdex75 increase 10/300 GL gel filtration column (GE/Amersham Biosciences, USA) at a flow rate of 0.5 mL/min. Samples were monitored by SDS-polyacrylamide gel electrophoresis, pooled, concentrated in the gel filtration buffer (50 mM Hepes pH 7.5, 150 mM NaCl, 1 mM TCEP), aliquoted, flash frozen in liquid nitrogen and stored at −80 °C for DEC-Tec selection and assay validation.

1b) DEC-Tec affinity selection with bromodomain proteins. To identify BRDT-BD2 selective compounds, we screened our DEC-Tec library pool in five tubes: 1) absence of bromodomain proteins (bead binding non-target control, NTC), 2) presence of His-BRDT-BD2 at 0.3 μM, 3) presence of His-

BRDT-BD1 at 0.3 μM (a counter-screen for bromodomain selective compounds), 4) presence of His-BRDT-BD2 plus JQ1 at 100 μM, and 5) presence of His-BRDT-BD1 plus JQ1 at 100 μM. JQ1 binds to BRDT bromodomains and the addition of JQ1 can block the binding of compounds in the library to bromodomain functional site so as to serve as controls. To initiate the screening experiment, the pool of our DEC-Tec libraries was quantitated using quantitative PCR (qPCR) so that each screening tube would start with the same amount of library molecules with each compound having one million copies. Three rounds of DEC-Tec selection have been performed to improve ligand enrichment. The DNA barcode from the last round of selection was PCR amplified and sequenced to identify the linked drug-like binders. In brief, DEC-Tec screening and sequencing will be performed as follows: 1) His-BRDT-BD2 or His-BRDT-BD1 at designated concentration was incubated with DEC-Tec libraries in 50 mM HEPES buffer, pH 7.5, containing 150 mM NaCl, 10 mM imidazole, 1 mM TCEP, 1 mM CHAPS and 0.1 mg/ml sheared salmon sperm DNA for 45 min at room temperature with continuous shaking; 2) His-BRDT-BD2 or BD1 along with the binding molecules were then captured instantaneously with HisPur Ni-NTA magnetic beads; 3) The Ni-NTA magnetic beads were washed 1 time with the aforementioned selection buffer without sheared salmon sperm DNA by brief and vigorous vortex; 4) To dissociate the binding molecules from His-tagged protein, Ni-NTA magnetic beads in 100 μL washing buffer were heated at 80 °C for 10 min with continuous shaking; 5) The resulting eluent, in which protein binding molecules are enriched, were further incubated with fresh His-BRDT-BD2 or BD1 to initiate another round selection following the same protocol described above; 6) After the last round of selection, the eluted encoding oligonucleotides were amplified using Platinum Taq DNA Polymerase High Fidelity using primers that incorporate complementary sequences to the library headpiece or tailpiece along with the Illumina READ 1 or READ 2 sequences required for clustering and Illumina sequencing; 7) The amplified DNA were cleaned by Agencourt AMPure XP beads and quantitated with Agilent high sensitivity DNA kit using a Bioanalyzer; 8) DNA was then denatured and sequenced along with a 3% PhiX spike-in in a single-read 105-cycle sequencing on an Illumina NextSeq 500 instrument; 9) The FASTQ format sequencing data was generated through Illumina BaseSpace and decoded and analyzed. Small molecules identified from the above screens were resynthesized off of DNA and evaluated in *in vitro* assays.

2. Assays

2a) Bromodomain proximity assay. The AlphaScreen™ assay was performed following previous publication (1) with minor modifications from the manufacturer's protocol (PerkinElmer, USA). A 20-µL reaction was set up in a PerkinElmer 384-well AlphaPlate where His-bromodomain at 10 nM was incubated with biotinylated JQ1 at 10 nM, nickel chelate acceptor beads at 12.5 µg/mL, and tested compound at various concentrations for 15 min at room temperature, followed by the addition of streptavidin donor beads at 12.5 µg/mL and another 60-min incubation at room temperature. The plate was read on a Tecan Infinite M1000 Pro plate reader.

2b) Thermal shift assay. The dye SYPRO Orange (ThermoFisher Scientific, USA) was used to perform the protein thermal shift assay. The assay was set up on a 384-well Roche plate where His-bromodomain at a concentration of 2 µM was incubated with the test compound at various concentrations, and SYPRO Orange dye at 5 × in a 10-µL reaction. The melting curve experiment and data analysis was run on a Roche Lightcycler 480 real-time PCR instrument.

2c) NanoBRET target engagement intracellular BET bromodomain assay. The NanoLuc-BRDT-BD1 and NanoLuc-BRDT-BD2 vectors were constructed by subcloning the same BRDT bromodomain sequences applied for protein expression into the NanoLuc-BRD4-BD1 and NanoLuc-BRD4-BD2 vectors (Promega, USA) to replace the corresponding BRD4 bromodomain sequences. The NanoBRET tracer competition assay was performed in transiently transfected HEK293 cells expressing each NanoLuc-bromodomain vector on a 384-well plate following the manufacturer's protocol (Promega, USA). Tracer titration was performed for each NanoLuc fusion vector to determine the optimized tracer concentration. The phusion protein was allowed to express for 36 h. The cells were then preincubated with tested compounds at different concentrations for 2 h followed by 2 h of incubation with tracer. Freshly prepared NanoBRET Nano-Glo substrate plus extracellular NanoLuc inhibitor were then added to initiate the subsequent bioluminescence resonance energy transfer (BRET) measurements using a CLARIOstar Plus BMG LABTECH plate reader. Data analysis was done by measuring the ratio of acceptor emission to donor emission (BRET ratio) and normalized by subtracting no-tracer-control-background.

2d) Metabolic stability assay in liver microsomes. Compounds **3–10**, **15**, **17** and **20–22** (2.0 µM) were incubated in the mouse or human liver microsomes (0.5 mg protein/mL) at 37 °C. The samples are collected at specific time-points 0, 5, 10, 20, 40 and 60 min. The reactions are terminated by adding equivalent volume of ice-cold CH_3OH and vortexed. The reaction mixtures are centrifuged at rcf 15,000 for 15 min. Five µL of the supernatant was analyzed by UHPLC-Q Exactive Orbitrap MS (Thermo Fisher Scientific, USA) equipped with 50 mm × 4.6 mm column (XDB C-18, Agilent Technologies, USA). The column temperature was maintained at 40 °C. The flow rate was at 0.3 mL/min with a 30% mobile phase (acetonitrile containing 0.1% formic acid). Q Exactive MS was operated in positive or negative mode with electrospray ionization. Ultra-pure nitrogen was applied as the sheath (45 arbitrary unit), auxiliary (10 arbitrary unit), sweep (1.0 arbitrary unit) and the collision gas. The capillary gas temperature was set at 275 °C and the capillary voltage was set at 3.7 kV. MS data was acquired from 80 to 1,200 Da in profile mode.

2e) BROMOscan bromodomain profiling. BROMOscan bromodomain profiling was provided by Eurofins DiscoverX Corp. (San Diego, CA, USA, http://www.discoverx.com). Determination of the Kd between test compounds and DNA tagged bromodomains was achieved through binding competition against a proprietary reference immobilized ligand.

3. Crystallography

BRDT-BD2 was co-crystallized with CDD-1102 or CDD-1302 by hanging drop vapor diffusion method. For crystallization, the purified BRDT-BD2 at 10 mg/mL was mixed with 2 and 4 molar excess of CDD-1102 and CDD-1302, respectively. The protein-inhibitor mixture was concentrated using Amicon Ultra-15 centrifugal filters (Millipore Sigma) to 30 mg/mL. Mosquito (TTP labtech) was used to dispense equal volumes of protein and reservoir (250 nL each) against 70 µL reservoir buffer in 96 wells crystallization tray (96-Well clear polystyrene microplate from SPT labtech). For the CDD-1102 complex, crystals were observed after 10 days in drop containing 0.8 M succinic acid (pH 7.0), whereas for the CDD-1302 complex, crystals were observed after 15 days in drop containing 1.1 M sodium malonate 0.1 M, HEPES pH 7.0, 0.5 % *v/v* Jeffamine ED-2001. The diffraction data were collected at Advanced Light Source (ALS), Beam Line 5.0.2 (UC Berkeley, USA) at wavelength (λ) = 1.0000 Å, by using an ADSC Q210 CCD detector. The data were integrated and scaled by using iMosflm and SCALA, respectively (2, 3). The crystal structures of the CDD-1102 and CDD-1302 complexes were determined by molecular replacement (4) using crystal structure of the human C-terminal bromodomain BRD2 (PDB ID: 4UYG) and the determined structure of the CDD-1102 complex as search models. The final models have gone through several rounds of refinement using phenix.refine (5) followed by manual model building using COOT (6). For all structural analysis and figure preparation, the visualization program PyMOL was used (7). The data collection and refinement statistics are summarized in Table S2.

4. Syntheses of Compounds

4a) Materials and instrumentation. All reactions involving air-sensitive reagents were carried out in anhydrous solvents under an atmosphere of nitrogen. Reagents and solvents purchased from commercial supplies were used as received. Reactions were monitored by thin-layer chromatography (TLC) on Baker-flex® silica gel plates (IB2-F) using UV-light (254 and 365 nm) detection or high-performance liquid chromatography/mass spectrometry (HPLC-MS). Column chromatography was carried out using Teledyne ISCO CombiFlash system equipped with either a silica or C-18 column. NMR spectra were recorded at room temperature using a Bruker Avance III HD 600 MHz spectrometer (^{1}H NMR at 600 MHz and ^{13}C NMR at 150 MHz) or a Bruker Avance III HD 800 MHz spectrometer (^{13}C NMR at 200 MHz). Chemical shifts (δ) are reported in parts per million (ppm) with reference to solvent signals [^{1}H-NMR: DMSO-d_6 (2.50 ppm); ^{13}C-NMR: DMSO-d_6 (39.51 ppm)]. Signal patterns are reported as s (singlet), d (doublet), t (triplet), q (quartet), h (heptet), m (multiplet) and br (broad). Coupling constants (*J*) are given in Hz. HRMS measurements were performed using ThermoFisher Scientific Q Exactive instrument. Abbreviations presented in experimental procedures are referred to the following definitions: *t*-BuOK, potassium *tert*-butylate; CH_2Cl_2, dichloromethane; CH_3CN, acetonitrile; CH_3OH, methanol; DIEA, *N,N*-diisopropylethylamine; DME, 1,2-dimethoxyethane; DMF, *N,N*-dimethylformamide; EtOAc, ethyl acetate; HATU, *O*-(7-azabenzotriazol-1-yl)-*N,N,N′,N′*-tetramethyluronium hexafluorophosphate; $LiOH{\cdot}H_2O$, lithium hydroxide monohydrate; $NaOH_{(aq)}$, aqueous sodium hydroxide solution; $Na_2CO_{3(sat)}$, saturated aqueous sodium carbonate solution; Na_2SO_4, sodium sulfate; $NaHCO_3$, sodium bicarbonate; NBS, *N*-bromosuccinimide; $Pd(dppf)Cl_2{\cdot}CH_2Cl_2$, [1,1′-bis(diphenylphosphino)ferrocene]dichloropalladium(II) dichloromethane complex; TEA, triethylamine; THF, tetrahydrofuran.

4b) General procedure for the Suzuki-Miyaura reaction: C-C bond formation between aryl halides and boronic acid derivatives. An oven-dried microwave vial, which was equipped with a magnetic stir bar, was charged with aryl halide (1.0 equiv.), boronic acid derivative (1.5−2.0 equiv.) and $Pd(dppf)Cl_2{\cdot}CH_2Cl_2$ (10 mol%). The vial was sealed with a microwave cap, evacuated and backfilled with nitrogen. Anhydrous DME and nitrogen-flushed $Na_2CO_{3(sat)}$ (*v/v*, 1:1) were then sequentially added *via* syringe. The mixture was vacuum-purged and refilled with nitrogen for three cycles and then heated by microwave reactor at 110 ºC for 1 h (or at indicated temperature for indicated period of time). The resulting mixture was diluted with EtOAc and washed with water. The organic layer was dried over anhydrous Na_2SO_4, filtered, concentrated *in vacuo* and purified by column chromatography using a silica-packed column with CH_3OH-CH_2Cl_2 gradient and/or C-18 column with water-CH_3CN gradient to afford desired cross-coupling product.

4c) Synthesis of intermediate 2a:

Step 1: *N*[1]-(2-Methoxy-5-nitrophenyl)-*N*[4]-methylterephthalamide

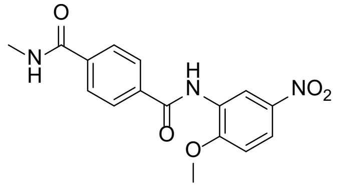

Chemical Formula: $C_{16}H_{15}N_3O_5$
Exact Mass: 329.1012

To a solution of 4-(methylcarbamoyl)benzoic acid (198 mg, 1.1 mmol, 1.0 equiv.), 2-methoxy-5-nitroaniline (**1a**) (277 mg, 1.65 mmol, 1.5 equiv.) and HATU (502 mg, 1.32 mmol, 1.2 equiv.) in anhydrous DMF (2 mL) was added DIEA (290 µL, 1.65 mmol, 1.5 equiv.) under nitrogen. The reaction mixture was stirred at room temperature for 16 h and then quenched by the addition of water. The aqueous layer was extracted twice with EtOAc, and the combined organic extracts were washed with brine, dried over anhydrous Na_2SO_4, filtered and concentrated. The residue was purified by column chromatography on silica gel

(CH_3OH/CH_2Cl_2, 1:99 to 5:95) to afford *N*[1]-(2-methoxy-5-nitrophenyl)-*N*[4]-methylterephthalamide (364 mg, 99%) as a pale yellow solid; ^{1}H NMR (600 MHz, DMSO-*d*$_6$) δ 9.90 (s, 1H), 8.77 (d, *J* = 2.6 Hz, 1H), 8.63 (d, *J* = 4.4 Hz, 1H), 8.15 (dd, *J* = 9.2, 2.6 Hz, 1H), 8.04 (d, *J* = 8.2 Hz, 2H), 7.97 (d, *J* = 8.2 Hz, 2H), 7.34 (d, *J* = 9.2 Hz, 1H), 4.00 (s, 3H), 2.82 (d, *J* = 4.4 Hz, 3H); HRMS (ESI) *m/z* calcd for $C_{16}H_{16}N_3O_5$ [M + H]$^+$ 330.1084, found 330.1079.

Step 2: *N*[1]-(5-Amino-2-methoxyphenyl)-*N*[4]-methylterephthalamide

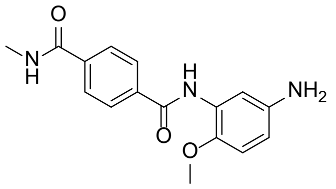

Chemical Formula: $C_{16}H_{17}N_3O_3$
Exact Mass: 299.1270

To a solution of *N*[1]-(2-methoxy-5-nitrophenyl)-*N*[4]-methylterephthalamide (344 mg, 1 mmol, 1.0 equiv.) and acetic acid (1.5 mL) in CH_3OH (5 mL) was added zinc powder (650 mg, 10 mmol, 10.0 equiv.) at 0 ºC with vigorous stirring. The resulting mixture was allowed to warm up to room temperature and stirred for 1 h. The excess zinc powder was then filtered off through a pad of Celite, and the resulting solution was neutralized to pH 7 with 4M $NaOH_{(aq)}$. The residue was concentrated *in vacuo* and purified by column chromatography on silica gel (CH_3OH/CH_2Cl_2, 1:99 to 10:90) to afford *N*[1]-(5-amino-2-methoxyphenyl)-*N*[4]-methylterephthalamide (90 mg, 29%) as a yellow solid; ^{1}H NMR (600 MHz, DMSO-*d*$_6$) δ 9.32 (s, 1H), 8.60−8.58 (m, 1H), 7.99 (d, *J* = 8.4 Hz, 2H), 7.94 (d, *J* = 8.4 Hz, 2H), 7.14 (d, *J* = 2.6 Hz, 1H), 6.80 (d, *J* = 8.7 Hz, 1H), 6.38 (dd, *J* = 8.7, 2.6 Hz, 1H), 4.75 (br s, 2H), 3.70 (s, 3H), 2.81 (d, *J* = 4.6 Hz, 3H); HRMS (ESI) *m/z* calcd for $C_{16}H_{18}IN_3O_3$ [M + H]$^+$ 300.1342, found 300.1338.

Step 3: *N*[1]-(5-(3-Iodo-1-methyl-1*H*-indazole-5-carboxamido)-2-methoxyphenyl)-*N*[4]-methylterephthalamide (2a)

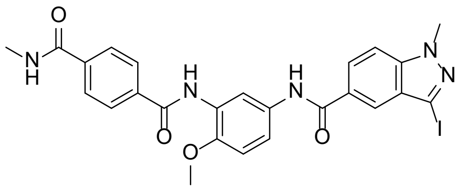

Chemical Formula: $C_{25}H_{22}IN_5O_4$
Exact Mass: 583.0716

To a solution of *N*[1]-(5-amino-2-methoxyphenyl)-*N*[4]-methylterephthalamide (60 mg, 0.2 mmol, 1.0 equiv.), 3-iodo-1-methyl-1*H*-indazole-5-carboxylic acid (67 mg, 0.22 mmol, 1.1 equiv.) and HATU (91 mg, 0.24 mmol, 1.2 equiv.) in anhydrous DMF (1.5 mL) was added DIEA (53 μL, 0.3 mmol, 1.5 equiv.) under nitrogen. The reaction mixture was stirred at room temperature for 16 h and then quenched by the addition of water. The aqueous layer was extracted twice with EtOAc, and the combined organic extracts were washed with brine, dried over anhydrous Na_2SO_4, filtered and concentrated. The residue was purified by column chromatography on silica gel (CH_3OH/CH_2Cl_2, 1:99 to 5:95) to afford **2a** (111 mg, 95%) as a pale yellow solid; ^{1}H NMR (600 MHz, DMSO-*d*$_6$) δ 10.37 (s, 1H), 9.64 (s, 1H), 8.61 (d, *J* = 4.4 Hz, 1H), 8.20 (d, *J* = 2.0 Hz, 1H), 8.17 (s, 1H), 8.09 (d, *J* = 8.8, 1H), 8.05 (d, *J* = 8.2 Hz, 2H), 7.96 (d, *J* = 8.2 Hz, 2H), 7.79 (d, *J* = 8.8 Hz, 1H), 7.69 (dd, *J* = 9.0, 2.0 Hz, 1H), 7.11 (d, *J* = 9.0 Hz, 1H), 4.12 (s, 3H), 3.85 (s, 3H), 2.82 (d, *J* = 4.4 Hz, 3H); HRMS (ESI) *m/z* calcd for $C_{25}H_{23}IN_5O_4$ [M + H]$^+$ 584.0789, found 584.0782.

4d) Synthesis of intermediate 2b:

Step 1: *N*[1]-Methyl-*N*[4]-(2-methyl-5-nitrophenyl)terephthalamide

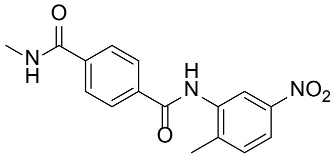

Chemical Formula: $C_{16}H_{15}N_3O_4$
Exact Mass: 313.1063

To a solution of 4-(methylcarbamoyl)benzoic acid (900 mg, 5 mmol, 1.0 equiv.), 2-methyl-5-nitroaniline (**1b**) (913 mg, 6 mmol, 1.2 equiv.) and HATU (2.28 g, 6 mmol, 1.2 equiv.) in anhydrous DMF (10 mL) was added DIEA (1.3 mL, 7.5 mmol, 1.5 equiv.) under nitrogen. The reaction mixture was stirred at room temperature for 16 h and then quenched by the addition of water. The resulting precipitate was collected, washed with ether, and

dried *in vacuo* to afford *N*[1]-methyl-*N*[4]-(2-methyl-5-nitrophenyl)terephthalamide (776 mg, 50%) as a pale yellow solid; ^{1}H NMR (600 MHz, DMSO-*d*$_6$) δ 10.26 (s, 1H), 8.63 (d, *J* = 4.3 Hz, 1H), 8.36 (d, *J* = 1.1 Hz, 1H), 8.07−8.04 (m, 3H), 7.99 (d, *J* = 8.1 Hz, 2H), 7.58 (d, *J* = 8.5 Hz, 1H), 2.82 (d, *J* = 4.3 Hz, 3H), 2.40 (s, 3H); HRMS (ESI) *m/z* calcd for $C_{16}H_{16}N_3O_4$ $[M + H]^+$ 314.1135, found 314.1126.

Step 2: *N*^{1}-(5-Amino-2-methylphenyl)-*N*^{4}-methylterephthalamide

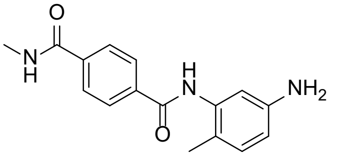

Chemical Formula: $C_{16}H_{17}N_3O_2$
Exact Mass: 283.1321

To a solution of *N*[1]-methyl-*N*[4]-(2-methyl-5-nitrophenyl)terephthalamide (690 mg, 2.2 mmol, 1.0 equiv.) and acetic acid (3.3 mL) in THF/CH_3OH (15 mL, *v/v* = 2:1) was added zinc powder (1.44 g, 22 mmol, 10.0 equiv.) at 0 ºC with vigorous stirring. The resulting mixture was allowed to warm up to room temperature and stirred for 1 h. The excess zinc powder was then filtered off through a pad of Celite, and the filtrate was neutralized to pH 7 with 4M $NaOH_{(aq)}$ and concentrated. The resulting residue was washed with water and dried *in vacuo* to afford *N*[1]-(5-amino-2-methylphenyl)-*N*[4]-methylterephthalamide (543 mg, 87%) as a pale yellow solid, which was directly used in the next step without further purification.

Step 3: *N*^{1}-(5-(3-Iodo-1-methyl-1*H*-indazole-5-carboxamido)-2-methylphenyl)-*N*^{4}-methylterephthalamide (2b)

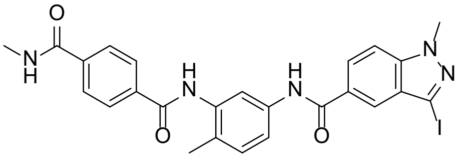

Chemical Formula: $C_{25}H_{22}IN_5O_3$
Exact Mass: 567.0767

To a solution of *N*[1]-(5-amino-2-methylphenyl)-*N*[4]-methylterephthalamide (255 mg, 0.9 mmol, 1.0 equiv.), 3-iodo-1-methyl-1*H*-indazole-5-carboxylic acid (270 mg, 0.9 mmol, 1.0 equiv.) and HATU (411 mg, 1.08 mmol, 1.2 equiv.) in anhydrous DMF (3 mL) was added DIEA (188 μL, 1.08 mmol, 1.2 equiv.) under nitrogen. The reaction mixture was stirred at room temperature for 16 h and then quenched by the addition of water. The precipitate was collected and washed with water. The aqueous solution was extracted twice with EtOAc, and the combined organic extracts were washed with brine, dried over anhydrous Na_2SO_4, filtered and concentrated. The residue and collected precipitate were combined and purified by column chromatography on silica gel (CH_3OH/CH_2Cl_2, 1:99 to 5:95) to afford **2b** (420 mg, 82%) as a pale yellow solid; ^{1}H NMR (600 MHz, DMSO-*d*$_6$) δ 10.41 (s, 1H), 10.06 (s, 1H), 8.62 (d, *J* = 4.0 Hz, 1H), 8.17 (s, 1H), 8.09−8.06 (m, 3H), 7.98 (d, *J* = 8.0 Hz, 2H), 7.88 (s, 1H), 7.79 (d, *J* = 8.8 Hz, 1H), 7.64 (d, *J* = 8.3 Hz, 1H), 7.27 (d, *J* = 8.3 Hz, 1H), 4.12 (s, 3H), 2.82 (d, *J* = 4.0 Hz, 3H), 2.23 (s, 3H); HRMS (ESI) *m/z* calcd for $C_{25}H_{23}IN_5O_3$ $[M + H]^+$ 568.0840, found 568.0826.

4e) Syntheses of DEC-Tec hits 3−12 and compounds 13−15:

***N*^{1}-(2-Methoxy-5-(1-methyl-3-(1-methyl-1*H*-indol-2-yl)-1*H*-indazole-5-carboxamido)phenyl)-*N*^{4}-methylterephthalamide (3)**

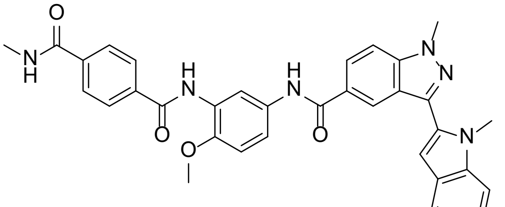

3

Chemical Formula: $C_{34}H_{30}N_6O_4$
Exact Mass: 586.2329

The general procedure employed for the Suzuki-Miyaura reaction was followed using aryl iodide **2a** (29 mg, 0.05 mmol, 1.0 equiv.), (1-methyl-1*H*-indol-2-yl)boronic acid (17 mg, 0.1 mmol, 2.0 equiv.) and Pd(dppf)$Cl_2 \cdot CH_2Cl_2$ (4 mg, 0.005 mmol, 0.1 equiv.) in DME/$Na_2CO_{3(sat)}$ (0.4 mL, *v/v* = 1:1) at 90 ºC for 5 h. Isolation and purification afforded title compound **3** (10 mg, 34%) as a pale yellow solid; ^{1}H NMR (600 MHz, DMSO-*d*$_6$) δ 10.37 (s, 1H), 9.64 (s, 1H), 8.67 (s, 1H), 8.62 (s, 1H), 8.19 (s, 1H), 8.11 (d, *J* = 8.6 Hz, 1H), 8.04 (d, *J* = 7.4 Hz, 2H), 7.96 (d, *J* = 7.4 Hz, 2H), 7.86 (d, *J* = 8.6 Hz, 1H), 7.69 (d, *J* = 7.7 Hz, 2H), 7.57 (d, *J* = 7.9 Hz, 1H), 7.26−7.22 (m, 2H), 7.11 (d, *J* =

7.7 Hz, 2H), 4.22 (s, 3H), 4.08 (s, 3H), 3.85 (s, 3H), 2.82 (s, 3H); ^{13}C NMR (150 MHz, DMSO-d_6) δ 165.9, 165.2, 164.5, 148.1, 141.6, 138.2, 137.5, 137.1, 136.6, 132.0, 131.3, 128.2, 127.6 (2 ×), 127.5, 127.2 (2 ×), 126.4, 126.3, 122.1, 121.7, 121.1, 120.5, 119.8, 118.5, 117.9, 111.3, 110.2, 110.0, 102.9, 56.0, 36.0, 31.7, 26.3; HRMS (ESI) *m/z* calcd for $C_{34}H_{31}IN_6O_4$ [M + H]$^+$ 587.2401, found 587.2401.

N[1]-(5-(3-(4-Amino-2-methylphenyl)-1-methyl-1*H*-indazole-5-carboxamido)-2-methoxyphenyl)-*N*[4]-methylterephthalamide (4)

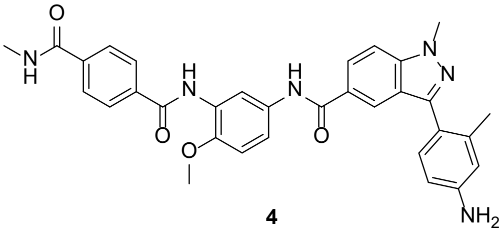

Chemical Formula: $C_{32}H_{30}N_6O_4$
Exact Mass: 562.2329

The general procedure employed for the Suzuki-Miyaura reaction was followed using aryl iodide **2a** (13 mg, 0.022 mmol, 1.0 equiv.), 3-methyl-4-(4,4,5,5-tetramethyl-1,3,2-dioxaborolan-2-yl)aniline (10 mg, 0.044 mmol, 2.0 equiv.) and Pd(dppf)Cl_2·CH_2Cl_2 (1.6 mg, 0.002 mmol, 0.1 equiv.) in DME/$Na_2CO_{3(sat)}$ (0.4 mL, *v/v* = 1:1) at 85 ºC for 90 min. Isolation and purification afforded title compound **4** (8.5 mg, 68%) as a pale yellow solid; ^{1}H NMR (600 MHz, DMSO-d_6) δ 10.25 (s, 1H), 9.62 (s, 1H), 8.61 (d, *J* = 4.4 Hz, 1H), 8.31 (s, 1H), 8.15 (d, *J* = 2.0 Hz, 1H), 8.04−8.02 (m, 3H), 7.95 (d, *J* = 8.2 Hz, 2H), 7.73 (d, *J* = 8.8 Hz, 1H), 7.66 (dd, *J* = 9.0, 2.0 Hz, 1H), 7.23 (d, *J* = 8.0 Hz, 1H), 7.08 (d, *J* = 9.0 Hz, 1H), 6.58−6.55 (m, 2H), 5.24 (s, 2H), 4.10 (s, 3H), 3.83 (s, 3H), 2.81 (d, *J* = 4.4 Hz, 3H), 2.25 (s, 3H); ^{13}C NMR (150 MHz, DMSO-d_6) δ 165.9, 165.3, 164.4, 148.8, 148.0, 145.6, 141.5, 137.1, 137.0, 136.6, 132.2, 131.1, 127.6 (2 ×), 127.2 (2 ×), 127.0, 126.2, 125.7, 121.9, 121.4, 119.0, 118.4, 117.9, 115.9, 111.5, 111.3, 109.5, 56.0, 35.5, 26.3, 20.6; HRMS (ESI) *m/z* calcd for $C_{32}H_{31}N_6O_4$ [M + H]$^+$ 563.2401, found 563.2387.

N[1]-(5-(3-(2,4-Dimethylphenyl)-1-methyl-1*H*-indazole-5-carboxamido)-2-methoxyphenyl)-*N*[4]-methylterephthalamide (5)

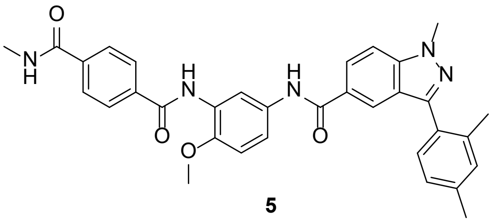

Chemical Formula: $C_{33}H_{31}N_5O_4$
Exact Mass: 561.2376

The general procedure employed for the Suzuki-Miyaura reaction was followed using aryl iodide **2a** (13 mg, 0.022 mmol, 1.0 equiv.), (2,4-dimethylphenyl)boronic acid (7 mg, 0.044 mmol, 2.0 equiv.) and Pd(dppf)Cl_2·CH_2Cl_2 (1.6 mg, 0.002 mmol, 0.1 equiv.) in DME/$Na_2CO_{3(sat)}$ (0.4 mL, *v/v* = 1:1). Isolation and purification afforded title compound **5** (3.5 mg, 28%) as a yellow solid; ^{1}H NMR (600 MHz, DMSO-d_6) δ 10.25 (s, 1H), 9.62 (s, 1H), 8.62 (d, *J* = 4.4 Hz, 1H), 8.32 (s, 1H), 8.14 (d, *J* = 1.9 Hz, 1H), 8.06 (d, *J* = 8.9, 1H), 8.03 (d, *J* = 8.2 Hz, 2H), 7.95 (d, *J* = 8.2 Hz, 2H), 7.79 (d, *J* = 8.9 Hz, 1H), 7.66 (dd, *J* = 8.9, 1.9 Hz, 1H), 7.46 (d, *J* = 7.7 Hz, 1H), 7.23 (s, 1H), 7.19 (d, *J* = 7.7 Hz, 1H), 7.08 (d, *J* = 8.9 Hz, 1H), 4.15 (s, 3H), 3.83 (s, 3H), 2.81 (d, *J* = 4.4 Hz, 3H), 2.37 (s, 3H), 2.35 (s, 3H); ^{13}C NMR (150 MHz, DMSO-d_6) δ 165.9, 165.2, 164.4, 148.1, 144.7, 141.5, 137.6, 137.1, 136.6, 136.3, 132.1, 131.5, 130.3, 128.8, 127.6 (2 ×), 127.4, 127.2 (2 ×), 126.6, 126.2, 125.9, 121.7, 121.0, 118.4, 117.8, 111.3, 109.8, 55.9, 35.6, 26.3, 20.8, 20.3; HRMS (ESI) *m/z* calcd for $C_{33}H_{32}N_5O_4$ [M + H]$^+$ 562.2448, found 562.2442.

N[1]-(2-Methoxy-5-(1-methyl-3-(*o*-tolyl)-1*H*-indazole-5-carboxamido)phenyl)-*N*[4]-methylterephthalamide (6)

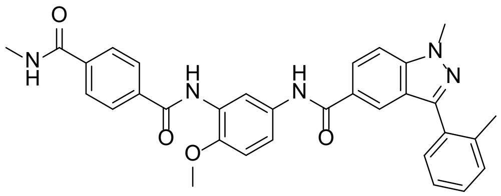

6

Chemical Formula: $C_{32}H_{29}N_5O_4$
Exact Mass: 547.2220

The general procedure employed for the Suzuki-Miyaura reaction was followed using aryl iodide **2a** (13 mg, 0.022 mmol, 1.0 equiv.), *o*-tolylboronic acid (6 mg, 0.044 mmol, 2.0 equiv.) and Pd(dppf)Cl_2·CH_2Cl_2 (1.6 mg, 0.002 mmol, 0.1 equiv.) in DME/$Na_2CO_{3(sat)}$ (0.4 mL, *v/v* = 1:1). Isolation and purification afforded title compound **6** (12 mg, 99%) as a pale yellow solid; ^{1}H NMR (600 MHz, DMSO-d_6) δ 10.26 (s, 1H), 9.62 (s, 1H), 8.61 (d, *J* = 4.4 Hz, 1H), 8.33 (s, 1H), 8.14 (d, *J* = 2.2 Hz, 1H), 8.07 (d, *J* = 8.9 Hz, 1H), 8.03 (d, *J* = 8.2 Hz, 2H), 7.95 (d, *J* = 8.2 Hz, 2H), 7.81 (d, *J* = 8.9 Hz, 1H), 7.65 (dd, *J* = 9.0, 2.2 Hz, 1H), 7.58−7.57 (m, 1H), 7.43−7.38 (m, 3H), 7.09 (d, *J* = 9.0 Hz, 1H), 4.16 (s, 3H), 3.83 (s, 3H), 2.81 (d, *J* = 4.4 Hz, 3H), 2.39 (s, 3H); ^{13}C NMR (150 MHz, DMSO-d_6) δ 165.9, 165.2, 164.4, 148.1, 144.7, 141.5, 137.1, 136.6, 136.6, 132.1, 131.7, 130.9, 130.3, 128.3, 127.6, 127.6 (2 ×), 127.2 (2 ×), 126.2, 126.0, 125.9, 121.7, 121.0, 118.4, 117.8, 111.3, 109.9, 55.9, 35.7, 26.3, 20.4; HRMS (ESI) *m/z* calcd for $C_{32}H_{30}N_5O_4$ [M + H]$^+$ 548.2292, found 548.2286.

***N*^1^-(5-(3-(4-Aminophenyl)-1-methyl-1*H*-indazole-5-carboxamido)-2-methoxyphenyl)-*N*^4^-methylterephthalamide (7)**

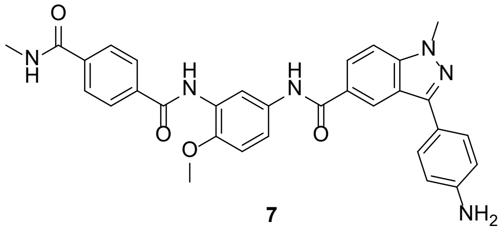

7

Chemical Formula: $C_{31}H_{28}N_6O_4$
Exact Mass: 548.2172

The general procedure employed for the Suzuki-Miyaura reaction was followed using aryl iodide **2a** (13 mg, 0.022 mmol, 1.0 equiv.), 4-(4,4,5,5-tetramethyl-1,3,2-dioxaborolan-2-yl)aniline (10 mg, 0.044 mmol, 2.0 equiv.) and Pd(dppf)Cl_2·CH_2Cl_2 (1.6 mg, 0.002 mmol, 0.1 equiv.) in DME/$Na_2CO_{3(sat)}$ (0.4 mL, *v/v* = 1:1). Isolation and purification afforded title compound **7** (7 mg, 58%) as a pale yellow solid; 1H NMR (600 MHz, DMSO-d6) δ 10.31 (s, 1H), 9.64 (s, 1H), 8.63 (s, 1H), 8.62 (d, *J* = 4.4 Hz, 1H), 8.18 (d, *J* = 2.2 Hz, 1H), 8.04 (d, *J* = 8.3 Hz, 2H), 8.01 (d, *J* = 8.9 Hz, 1H), 7.96 (d, *J* = 8.3 Hz, 2H), 7.72−7.71 (m, 3H), 7.69 (dd, *J* = 9.0, 2.2 Hz, 1H), 7.11 (d, *J* = 9.0 Hz, 1H), 6.73 (d, *J* = 8.5 Hz, 2H), 5.37 (s, 2H), 4.08 (s, 3H), 3.85 (s, 3H), 2.82 (d, *J* = 4.4 Hz, 3H); ^{13}C NMR (150 MHz, DMSO-d_6) δ 165.9, 165.4, 164.5, 149.0, 148.0, 144.7, 142.2, 137.1, 136.6, 132.2, 128.0 (2 ×), 127.6 (2 ×), 127.2 (2 ×), 127.2, 126.3, 125.7, 121.6, 120.3, 119.9, 118.4, 117.9, 114.1 (2 ×), 111.3, 109.6, 56.0, 35.4, 26.3; HRMS (ESI) *m/z* calcd for $C_{31}H_{29}N_6O_4$ [M + H]$^+$ 549.2244, found 549.2236.

***N*^1^-Methyl-*N*^4^-(2-methyl-5-(1-methyl-3-(1-methyl-1*H*-indol-2-yl)-1*H*-indazole-5-carboxamido)phenyl)terephthalamide (8)**

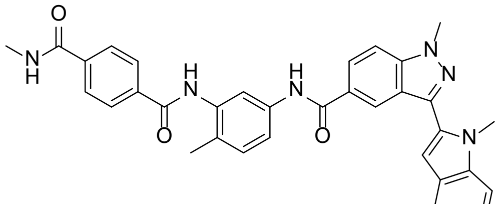

8

Chemical Formula: $C_{34}H_{30}N_6O_3$
Exact Mass: 570.2379

The general procedure employed for the Suzuki-Miyaura reaction was followed using aryl iodide **2b** (11 mg, 0.02 mmol, 1.0 equiv.), (1-methyl-1*H*-indol-2-yl)boronic acid (10 mg, 0.04 mmol, 2.0 equiv.) and Pd(dppf)Cl_2·CH_2Cl_2 (1.6 mg, 0.002 mmol, 0.1 equiv.) in DME/$Na_2CO_{3(sat)}$ (0.4 mL, *v/v* = 1:1) at 110 °C for 2 h. Isolation and purification afforded title compound **8** (8 mg, 71%) as a pale yellow solid; ^{1}H NMR (600 MHz, DMSO-d_6) δ 10.41 (s, 1H), 10.06 (s, 1H), 8.66 (s, 1H), 8.62 (d, *J* = 4.5 Hz, 1H), 8.09 (d, *J* = 8.9 Hz, 1H), 8.06 (d, *J* = 8.3 Hz, 2H), 7.97 (d, *J* = 8.3 Hz, 2H), 7.87−7.86 (m, 2H), 7.69 (d, *J* = 7.9 Hz, 1H), 7.65 (dd, *J* = 8.2, 1.9 Hz, 1H), 7.57 (d, *J* = 8.2 Hz, 1H), 7.28−7.24 (m, 2H), 7.21 (s, 1H), 7.12 (t, *J* = 7.4 Hz, 1H), 4.22 (s, 3H), 4.07 (s, 3H), 2.82 (d, *J* = 4.5 Hz,

3H), 2.23 (s, 3H); ^{13}C NMR (150 MHz, DMSO-d_6) δ 165.9, 165.5, 164.8, 141.6, 138.2, 137.6, 137.3, 137.1, 136.7, 136.2, 131.3, 130.2, 128.9, 128.2, 127.7 (2 ×), 127.5, 127.2 (2 ×), 126.5, 122.2, 121.7, 121.2, 120.5, 119.8, 118.9, 118.5, 110.2, 110.1, 103.0, 36.0, 31.7, 26.3, 17.5; HRMS (ESI) *m/z* calcd for $C_{34}H_{31}N_6O_3$ [M + H]$^+$ 571.2452, found 571.2445.

N^1-(5-(3-(4-Amino-2-methylphenyl)-1-methyl-1*H*-indazole-5-carboxamido)-2-methylphenyl)-N^4-methylterephthalamide (9, CDD-1102)

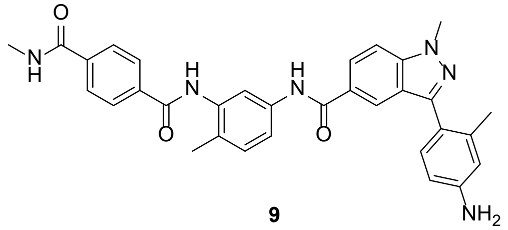

9

Chemical Formula: $C_{32}H_{30}N_6O_3$
Exact Mass: 546.2379

The general procedure employed for the Suzuki-Miyaura reaction was followed using aryl iodide **2b** (28 mg, 0.05 mmol, 1.0 equiv.), 3-methyl-4-(4,4,5,5-tetramethyl-1,3,2-dioxaborolan-2-yl)aniline (18 mg, 0.075 mmol, 1.5 equiv.) and Pd(dppf)$Cl_2 \cdot CH_2Cl_2$ (4 mg, 0.005 mmol, 0.1 equiv.) in DME/$Na_2CO_{3(sat)}$ (0.4 mL, *v/v* = 1:1). Isolation and purification afforded title compound **9** (15 mg, 53%) as a pale yellow solid; ^{1}H NMR (600 MHz, DMSO-d_6) δ 10.28 (s, 1H), 10.04 (s, 1H), 8.61 (d, *J* = 4.5 Hz, 1H), 8.31 (s, 1H), 8.05 (d, *J* = 8.3 Hz, 2H), 8.02 (d, *J* = 8.9 Hz, 1H), 7.97 (d, *J* = 8.3 Hz, 2H), 7.84 (d, *J* = 1.7 Hz, 1H), 7.74 (d, *J* = 8.9 Hz, 1H), 7.61 (dd, *J* = 8.3, 1.7 Hz, 1H), 7.25−7.22 (m, 2H), 6.58−6.55 (m, 2H), 5.25 (s, 2H), 4.10 (s, 3H), 2.82 (d, *J* = 4.5 Hz, 3H), 2.25 (s, 3H), 2.21 (s, 3H); ^{13}C NMR (150 MHz, DMSO-d_6) δ 165.9, 165.5, 164.7, 148.8, 145.6, 141.5, 137.4, 137.0, 137.0, 136.7, 136.1, 131.1, 130.1, 128.8, 127.6 (2 ×), 127.2 (2 ×), 126.9, 125.7, 121.9, 121.5, 118.9, 118.8, 118.4, 115.9, 111.5, 109.5, 35.5, 26.3, 20.6, 17.4; HRMS (ESI) *m/z* calcd for $C_{32}H_{31}N_6O_3$ [M + H]$^+$ 547.2452, found 547.2437.

N^1-(5-(3-(6-Aminopyridin-3-yl)-1-methyl-1*H*-indazole-5-carboxamido)-2-methylphenyl)-N^4-methylterephthalamide (10)

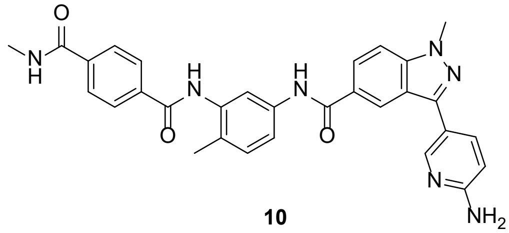

10

Chemical Formula: $C_{30}H_{27}N_7O_3$
Exact Mass: 533.2175

The general procedure employed for the Suzuki-Miyaura reaction was followed using aryl iodide **2b** (11 mg, 0.02 mmol, 1.0 equiv.), (6-aminopyridin-3-yl)boronic acid (6 mg, 0.04 mmol, 2.0 equiv.) and Pd(dppf)$Cl_2 \cdot CH_2Cl_2$ (1.6 mg, 0.002 mmol, 0.1 equiv.) in DME/$Na_2CO_{3(sat)}$ (0.4 mL, *v/v* = 1:1). Isolation and purification afforded title compound **10** (10 mg, 94%) as a pale yellow solid; ^{1}H NMR (600 MHz, DMSO-d_6) δ 10.37 (s, 1H), 10.05 (s, 1H), 8.64−8.60 (m, 3H), 8.06 (d, *J* = 8.3 Hz, 2H), 8.02 (dd, *J* = 8.9, 1.5 Hz, 1H), 7.99 (dd, *J* = 8.6, 2.4 Hz, 1H), 7.97 (d, *J* = 8.3 Hz, 2H), 7.86 (d, *J* = 2.0 Hz, 1H), 7.76 (d, *J* = 8.9 Hz, 1H), 7.63 (dd, *J* = 8.4, 2.0 Hz, 1H), 7.27 (d, *J* = 8.4 Hz, 1H), 6.62 (d, *J* = 8.6 Hz, 1H), 6.23 (s, 2H), 4.11 (s, 3H), 2.82 (d, *J* = 4.5 Hz, 3H), 2.23 (s, 3H); ^{13}C NMR (150 MHz, DMSO-d_6) δ 166.0, 165.5, 164.8, 159.5, 146.1, 142.4, 142.1, 137.4, 137.1, 136.7, 136.2, 135.9, 130.2, 128.8, 127.7 (2 ×), 127.4, 127.2 (2 ×), 126.0, 121.3, 119.8, 118.9, 118.5, 117.2, 109.9, 108.3, 35.6, 26.3, 17.5; HRMS (ESI) *m/z* calcd for $C_{30}H_{28}N_7O_3$ [M + H]$^+$ 534.2248, found 534.224.

N^1-(5-(3-(6-Aminopyridin-3-yl)-1-methyl-1*H*-indazole-5-carboxamido)-2-methylphenyl)-N^4-methylterephthalamide (11)

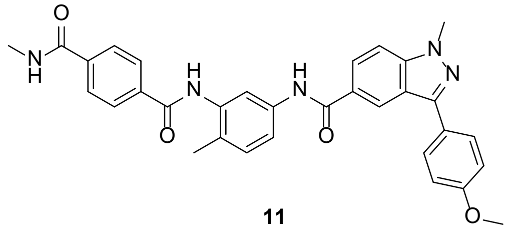

11

Chemical Formula: $C_{32}H_{29}N_5O_4$
Exact Mass: 547.2220

The general procedure employed for the Suzuki-Miyaura reaction was followed using aryl iodide **2b** (9 mg, 0.015 mmol, 1.0 equiv.), (4-methoxyphenyl)boronic acid (5 mg, 0.03 mmol, 2.0 equiv.) and Pd(dppf)Cl_2·CH_2Cl_2 (1.3 mg, 0.0015 mmol, 0.1 equiv.) in DME/$Na_2CO_{3(sat)}$ (0.4 mL, *v/v* = 1:1). Isolation and purification afforded title compound **11** (7 mg, 80%) as a white solid; ^{1}H NMR (600 MHz, DMSO-d_6) δ 10.38 (s, 1H), 10.06 (s, 1H), 8.66 (s, 1H), 8.61 (d, *J* = 4.5 Hz, 1H), 8.06 (d, *J* = 8.3 Hz, 2H), 8.03 (d, *J* = 8.9 Hz, 1H), 7.99−7.96 (m, 4H), 7.84 (d, *J* = 1.5 Hz, 1H), 7.78 (d, *J* = 8.9 Hz, 1H), 7.66 (dd, *J* = 8.4, 1.5 Hz, 1H), 7.27 (d, *J* = 8.4 Hz, 1H), 7.13 (d, *J* = 8.7 Hz, 2H), 4.13 (s, 3H), 3.83 (s, 3H), 2.82 (d, *J* = 4.5 Hz, 3H), 2.23 (s, 3H); ^{13}C NMR (200 MHz, DMSO-d_6) δ 166.0, 165.6, 164.8, 159.3, 143.7, 142.2, 137.4, 137.1, 136.7, 136.1, 130.2, 128.9, 128.4 (2 ×), 127.7, 127.7 (2 ×), 127.2 (2 ×), 126.0, 125.3, 121.3, 119.9, 118.8, 118.4, 114.5 (2 ×), 110.0, 55.3, 35.6, 26.3, 17.4; HRMS (ESI) *m/z* calcd for $C_{32}H_{30}N_5O_4$ [M + H]$^+$ 548.2292, found 548.2285.

N^1-Methyl-N^4-(2-methyl-5-(1-methyl-3-(pyridin-4-yl)-1*H*-indazole-5-carboxamido)phenyl)terephthalamide (12)

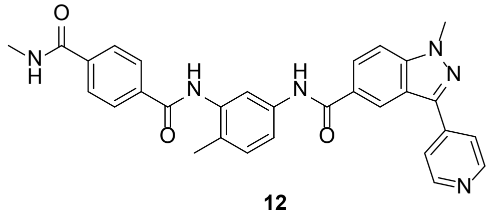

12

Chemical Formula: $C_{30}H_{26}N_6O_3$
Exact Mass: 518.2066

The general procedure employed for the Suzuki-Miyaura reaction was followed using aryl iodide **2b** (11 mg, 0.02 mmol, 1.0 equiv.), pyridin-4-ylboronic acid (4 mg, 0.03 mmol, 1.5 equiv.) and Pd(dppf)Cl_2·CH_2Cl_2 (1.6 mg, 0.002 mmol, 0.1 equiv.) in DME/$Na_2CO_{3(sat)}$ (0.4 mL, *v/v* = 1:1). Isolation and purification afforded title compound **12** (2.5 mg, 24%) as a pale yellow foam; ^{1}H NMR (600 MHz, DMSO-d_6) δ 10.42 (s, 1H), 10.06 (s, 1H), 8.79 (s, 1H), 8.75−8.74 (m, 2H), 8.61 (d, *J* = 4.5 Hz, 1H), 8.08−8.06 (m, 5H), 7.97 (d, *J* = 8.4 Hz, 2H), 7.89 (d, *J* = 8.8 Hz, 1H), 7.85 (d, *J* = 2.0 Hz, 1H), 7.67 (dd, *J* = 8.4, 2.0 Hz, 1H), 7.28 (d, *J* = 8.4 Hz, 1H), 4.21 (s, 3H), 2.82 (d, *J* = 4.5 Hz, 3H), 2.24 (s, 3H); ^{13}C NMR (150 MHz, DMSO-d_6) δ 166.4, 165.8, 165.3, 150.9 (2 ×), 142.9, 141.3, 140.3, 137.7, 137.5, 137.1, 136.6, 130.7, 129.4, 129.2, 128.1 (2 ×), 127.6 (2 ×), 126.8, 121.5 (2 ×), 121.3, 120.6, 119.3, 118.9, 111.1, 36.5, 26.8, 17.9; HRMS (ESI) *m/z* calcd for $C_{30}H_{27}N_6O_3$ [M + H]$^+$ 519.2139, found 519.2131.

N^1-(5-(3-(2-Aminopyrimidin-5-yl)-1-methyl-1*H*-indazole-5-carboxamido)-2-methylphenyl)-N^4-methylterephthalamide (13)

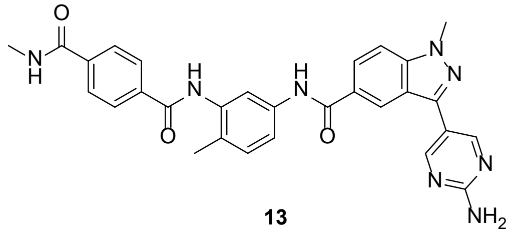

13

Chemical Formula: $C_{29}H_{26}N_8O_3$
Exact Mass: 534.2128

The general procedure employed for the Suzuki-Miyaura reaction was followed using aryl iodide **2b** (11 mg, 0.02 mmol, 1.0 equiv.), (2-aminopyrimidin-5-yl)boronic acid (4 mg, 0.03 mmol, 1.5 equiv.) and Pd(dppf)Cl_2·CH_2Cl_2 (1.6 mg, 0.002 mmol, 0.1 equiv.) in DME/$Na_2CO_{3(sat)}$ (0.4 mL, *v/v* = 1:1). Isolation and purification afforded title compound **13** (7.5 mg, 72%) as a pale yellow solid; ^{1}H NMR (600 MHz, DMSO-d_6) δ 10.36 (s, 1H), 10.06 (s, 1H), 8.89 (s, 2H), 8.66 (s, 1H), 8.62 (d, *J* = 3.4 Hz, 1H), 8.07−8.03 (m, 3H), 7.97 (d, *J* = 7.7 Hz, 2H), 7.86 (s, 1H), 7.79 (d, *J* = 8.8 Hz, 1H), 7.62 (d, *J* = 8.2 Hz, 1H), 7.27 (d, *J* = 8.2 Hz, 1H), 6.96 (s, 2H), 4.13 (s, 3H), 2.82 (d, *J* = 3.4 Hz, 3H), 2.23 (s, 3H); ^{13}C NMR (150 MHz, DMSO-d_6) δ 166.0, 165.3, 164.8, 163.1, 156.3 (2 ×), 142.1, 140.0,

137.3, 137.1, 136.7, 136.2, 130.2, 128.9, 127.7 (2 ×), 127.5, 127.2 (2 ×), 126.3, 121.0, 119.7, 119.0, 118.5, 115.8, 110.1, 35.7, 26.4, 17.5; HRMS (ESI) *m/z* calcd for $C_{29}H_{27}N_8O_3$ $[M + H]^+$ 535.2200, found 535.2191.

N^1-(5-(3-(6-Amino-2-methylpyridin-3-yl)-1-methyl-1*H*-indazole-5-carboxamido)-2-methylphenyl)-N^4-methylterephthalamide (14)

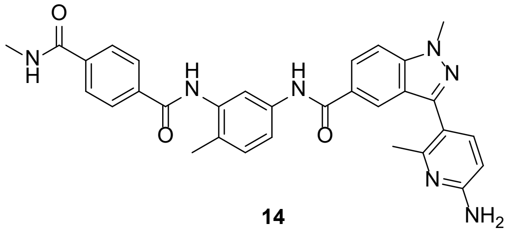

14

Chemical Formula: $C_{31}H_{29}N_7O_3$
Exact Mass: 547.2332

The general procedure employed for the Suzuki-Miyaura reaction was followed using aryl iodide **2b** (11 mg, 0.02 mmol, 1.0 equiv.), 6-methyl-5-(4,4,5,5-tetramethyl-1,3,2-dioxaborolan-2-yl)pyridin-2-amine (7 mg, 0.03 mmol, 1.5 equiv.) and Pd(dppf)Cl_2·CH_2Cl_2 (1.6 mg, 0.002 mmol, 0.1 equiv.) in DME/$Na_2CO_{3(sat)}$ (0.4 mL, *v/v* = 1:1). Isolation and purification afforded title compound **14** (8 mg, 73%) as a pale yellow solid; ^{1}H NMR (600 MHz, DMSO-*d*$_6$) δ 10.27 (s, 1H), 10.04 (s, 1H), 8.61 (d, *J* = 4.5 Hz, 1H), 8.31 (s, 1H), 8.06−8.03 (m, 3H), 7.96 (d, *J* = 8.3 Hz, 2H), 7.83 (d, *J* = 1.4 Hz, 1H), 7.76 (d, *J* = 8.9 Hz, 1H), 7.60 (dd, *J* = 8.5, 1.4 Hz, 1H), 7.55 (d, *J* = 8.3 Hz, 1H), 7.24 (d, *J* = 8.5 Hz, 1H), 6.45 (d, *J* = 8.3 Hz, 1H), 6.13 (br s, 2H), 4.12 (s, 3H), 2.82 (d, *J* = 4.5 Hz, 3H), 2.34 (s, 3H), 2.21 (s, 3H); ^{13}C NMR (200 MHz, DMSO-*d*$_6$) δ 166.0, 165.4, 164.8, 158.8, 154.5, 144.3, 141.6, 139.3, 137.4, 137.0, 136.7, 136.1, 130.2, 128.8, 127.7 (2 ×), 127.2 (2 ×), 127.1, 125.9, 121.7, 121.1, 118.9, 118.5, 114.6, 109.8, 105.4, 35.6, 26.3, 22.9, 17.4; HRMS (ESI) *m/z* calcd for $C_{31}H_{30}N_7O_3$ $[M + H]^+$ 548.2404, found 548.2393.

N^1-(5-(3-(6-Amino-4-methylpyridin-3-yl)-1-methyl-1*H*-indazole-5-carboxamido)-2-methylphenyl)-N^4-methylterephthalamide (15, CDD-1349)

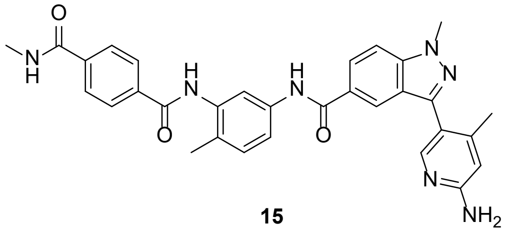

15

Chemical Formula: $C_{31}H_{29}N_7O_3$
Exact Mass: 547.2332

The general procedure employed for the Suzuki-Miyaura reaction was followed using aryl iodide **2b** (11 mg, 0.02 mmol, 1.0 equiv.), 4-methyl-5-(4,4,5,5-tetramethyl-1,3,2-dioxaborolan-2-yl)-2-pyridinamine hydrochloride (8 mg, 0.03 mmol, 1.5 equiv.) and Pd(dppf)Cl_2·CH_2Cl_2 (1.6 mg, 0.002 mmol, 0.1 equiv.) in DME/$Na_2CO_{3(sat)}$ (0.4 mL, *v/v* = 1:1). Isolation and purification afforded title compound **15** (3.2 mg, 29%) as a pale yellow solid; ^{1}H NMR (600 MHz, DMSO-*d*$_6$) δ 10.31 (s, 1H), 10.04 (s, 1H), 8.62 (d, *J* = 4.4 Hz, 1H), 8.36 (s, 1H), 8.11 (s, 1H), 8.06−8.03 (m, 3H), 7.97 (d, *J* = 8.2 Hz, 2H), 7.83 (d, *J* = 1.3 Hz, 1H), 7.77 (d, *J* = 8.9 Hz, 1H), 7.61 (dd, *J* = 8.4, 1.3 Hz, 1H), 7.24 (d, *J* = 8.4 Hz, 1H), 6.46 (s, 1H), 6.08 (s, 2H), 4.12 (s, 3H), 2.82 (d, *J* = 4.4 Hz, 3H), 2.25 (s, 3H), 2.22 (s, 3H); ^{13}C NMR (150 MHz, DMSO-*d*$_6$) δ 165.9, 165.4, 164.7, 159.6, 148.7, 146.3, 142.9, 141.5, 137.4, 137.0, 136.7, 136.1, 130.2, 128.8, 127.6 (2 ×), 127.2 (3 ×), 125.9, 121.8, 121.1, 118.8, 118.4, 116.7, 109.8, 108.9, 35.6, 26.3, 20.1, 17.4; HRMS (ESI) *m/z* calcd for $C_{31}H_{30}N_7O_3$ $[M + H]^+$ 548.2404, found 548.2393.

4f) Syntheses of compounds 16, 17 and 20:

Step 1: *N*-(3-Acetamido-4-methylphenyl)-3-iodo-1-methyl-1*H*-indazole-5-carboxamide

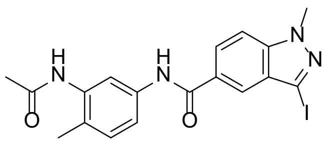

Chemical Formula: $C_{18}H_{17}IN_4O_2$
Exact Mass: 448.0396

To a solution of 3-iodo-1-methyl-1*H*-indazole-5-carboxylic acid (604 mg, 2 mmol, 1.0 equiv.), *N*-(5-amino-2-methylphenyl)acetamide (493 mg, 3 mmol, 1.5 equiv.) and HATU (913 mg, 2.4 mmol, 1.2 equiv.) in anhydrous DMF (2 mL) was added DIEA (700 µL, 4 mmol, 2.0 equiv.) under nitrogen. The reaction mixture was stirred at room temperature for 16 h and then quenched by the addition of water. The precipitate was collected, sequentially washed with water and ether and dried *in vacuo* to afford *N*-(3-acetamido-4-methylphenyl)-3-iodo-1-methyl-1*H*-indazole-5-carboxamide (810 mg, 90%) as a pale yellow solid, which was directly used in the next step without further purification; ^{1}H NMR (600 MHz, DMSO-d_6) δ 10.35 (s, 1H), 9.33 (s, 1H), 8.15 (s, 1H), 8.06 (d, J = 8.8 Hz, 1H), 7.87 (s, 1H), 7.78 (d, J = 8.8 Hz, 1H), 7.54 (d, J = 8.2 Hz, 1H), 7.17 (d, J = 8.2 Hz, 1H), 4.11 (s, 3H), 2.17 (s, 3H), 2.07 (s, 3H); HRMS (ESI) *m/z* calcd for $C_{18}H_{18}N_4O_2$ [M + H]$^+$ 449.0468, found 449.0461.

Step 2a: *N*-(3-Acetamido-4-methylphenyl)-3-(6-amino-4-methylpyridin-3-yl)-1-methyl-1*H*-indazole-5-carboxamide (16)

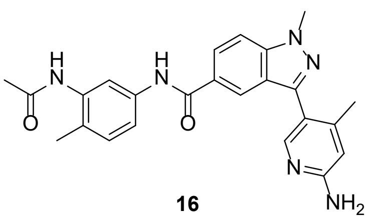

16

Chemical Formula: $C_{24}H_{24}N_6O_2$
Exact Mass: 428.1961

The general procedure employed for the Suzuki-Miyaura reaction was followed using *N*-(3-acetamido-4-methylphenyl)-3-iodo-1-methyl-1*H*-indazole-5-carboxamide (23 mg, 0.05 mmol, 1.0 equiv.), 4-methyl-5-(4,4,5,5-tetramethyl-1,3,2-dioxaborolan-2-yl)-2-pyridinamine hydrochloride (20 mg, 0.075 mmol, 1.5 equiv.) and Pd(dppf)Cl_2·CH_2Cl_2 (4 mg, 0.005 mmol, 0.1 equiv.) in DME/$Na_2CO_{3(sat)}$ (0.4 mL, *v/v* = 1:1). Isolation and purification afforded title compound **16** (9 mg, 42%) as a pale yellow solid; ^{1}H NMR (600 MHz, DMSO-d_6) δ 10.25 (s, 1H), 9.32 (s, 1H), 8.34 (s, 1H), 8.11 (s, 1H), 8.03 (d, J = 8.8 Hz, 1H), 7.82 (s, 1H), 7.75 (d, J = 8.8 Hz, 1H), 7.52 (d, J = 8.3 Hz, 1H), 7.15 (d, J = 8.3 Hz, 1H), 6.46 (s, 1H), 6.08 (s, 2H), 4.12 (s, 3H), 2.25 (s, 3H), 2.16 (s, 3H), 2.06 (s, 3H); ^{13}C NMR (150 MHz, DMSO-d_6) δ 168.2, 165.4, 159.7, 148.7, 146.4, 142.9, 141.6, 137.2, 136.4, 130.0, 127.2, 126.8, 126.0, 121.8, 121.1, 117.6, 117.5, 116.7, 109.7, 108.9, 35.6, 23.3, 20.1, 17.4; HRMS (ESI) *m/z* calcd for $C_{24}H_{25}N_6O_2$ [M + H]$^+$ 429.2033, found 429.2024.

Step 2b: *N*-(3-Acetamido-4-methylphenyl)-3-(6-aminopyridin-3-yl)-1-methyl-1*H*-indazole-5-carboxamide (17)

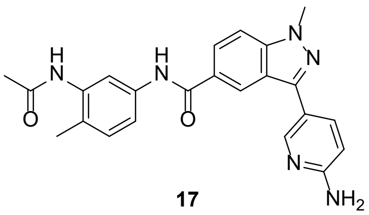

17

Chemical Formula: $C_{23}H_{22}N_6O_2$
Exact Mass: 414.1804

The general procedure employed for the Suzuki-Miyaura reaction was followed using *N*-(3-acetamido-4-methylphenyl)-3-iodo-1-methyl-1*H*-indazole-5-carboxamide (225 mg, 0.5 mmol, 1.0 equiv.), (6-aminopyridin-3-yl)boronic acid (104 mg, 0.75 mmol, 1.5 equiv.) and Pd(dppf)Cl_2·CH_2Cl_2 (41 mg, 0.05 mmol, 0.1 equiv.) in DME/$Na_2CO_{3(sat)}$ (4 mL, *v/v* = 1:1). Isolation and purification afforded title compound **17** (145 mg, 70%) as a white solid; ^{1}H NMR (600 MHz, DMSO-d_6) δ 10.31 (s, 1H), 9.32 (s, 1H), 8.62 (s, 2H), 8.03−8.01 (m, 2H), 7.85 (s, 1H), 7.75 (d, J = 8.8 Hz, 1H), 7.54 (d, J = 8.3 Hz, 1H), 7.17 (d, J = 8.3 Hz, 1H), 6.64 (d, J = 8.6 Hz, 1H), 6.31 (s, 2H), 4.10 (s, 3H), 2.18 (s, 3H), 2.07 (s, 3H); ^{13}C NMR (150 MHz, DMSO-d_6) δ 168.2, 165.4, 159.4, 145.9, 142.3, 142.1, 137.2, 136.4, 136.0, 130.0, 127.4, 126.7, 126.1, 121.3, 119.8, 117.5, 117.5, 117.2, 109.9, 108.4, 35.6, 23.3, 17.4; HRMS (ESI) *m/z* calcd for $C_{23}H_{23}N_6O_2$ [M + H]$^+$ 415.1877, found 415.1865.

Step 2c: *N*-(3-Acetamido-4-methylphenyl)-3-(4-amino-2-methylphenyl)-1-methyl-1*H*-indazole-5-carboxamide (20, CDD-1302)

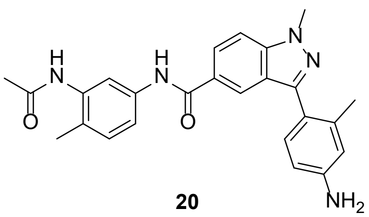

20

Chemical Formula: $C_{25}H_{25}N_5O_2$
Exact Mass: 427.2008

The general procedure employed for the Suzuki-Miyaura reaction was followed using *N*-(3-acetamido-4-methylphenyl)-3-iodo-1-methyl-1*H*-indazole-5-carboxamide (23 mg, 0.05 mmol, 1.0 equiv.), 3-methyl-4-(4,4,5,5-tetramethyl-1,3,2-dioxaborolan-2-yl)aniline (19 mg, 0.075 mmol, 1.5 equiv.) and Pd(dppf)Cl_2·CH_2Cl_2 (4 mg, 0.005 mmol, 0.1 equiv.) in DME/$Na_2CO_{3(sat)}$ (0.4 mL, *v/v* = 1:1). Isolation and purification afforded title compound **20** (18 mg, 84%) as a pale yellow solid; ^{1}H NMR (600 MHz, DMSO-d_6) δ 10.23 (s, 1H), 9.31 (s, 1H), 8.29 (s, 1H), 8.00 (d, *J* = 8.8 Hz, 1H), 7.82 (s, 1H), 7.72 (d, *J* = 8.8 Hz, 1H), 7.52 (d, *J* = 8.3 Hz, 1H), 7.22 (d, *J* = 8.1 Hz, 1H), 7.14 (d, *J* = 8.3 Hz, 1H), 6.58 (s, 1H), 6.56 (d, *J* = 8.1 Hz, 1H), 5.25 (s, 2H), 4.10 (s, 3H), 2.25 (s, 3H), 2.16 (s, 3H), 2.06 (s, 3H); ^{13}C NMR (150 MHz, DMSO-d_6) δ 168.1, 165.5, 148.8, 145.6, 141.5, 137.2, 137.0, 136.4, 131.1, 130.0, 126.9, 126.7, 125.7, 121.9, 121.5, 119.0, 117.4, 117.4, 115.9, 111.5, 109.5, 35.5, 23.3, 20.6, 17.4; HRMS (ESI) *m/z* calcd for $C_{25}H_{26}N_5O_2$ $[M + H]^+$ 428.2081, found 428.2070.

4g) Synthesis of compound 18:

Step 1: Methyl 1-ethyl-3-iodo-1*H*-indazole-5-carboxylate

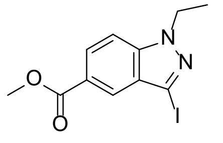

Chemical Formula: $C_{11}H_{11}IN_2O_2$
Exact Mass: 329.9865

To a solution of methyl 3-iodo-1*H*-indazole-5-carboxylate (91 mg, 0.3 mmol, 1.0 equiv.) and *t*-BuOK (40 mg, 0.36 mmol, 1.2 equiv.) in anhydrous THF (3 mL) was added iodoethane (30 µL, 0.36 mmol, 1.2 equiv.) at 0 °C under nitrogen. The reaction mixture was allowed to warm up to room temperature and stirred for 16 h. After being quenched by the addition of water, the mixture was extracted twice with EtOAc, and the combined organic extracts were washed with brine, dried over anhydrous Na_2SO_4, filtered and concentrated. The residue was purified by column chromatography on silica gel (EtOAc/hexane, 0:100 to 10:90) to afford methyl 1-ethyl-3-iodo-1*H*-indazole-5-carboxylate (48 mg, 48%) as a white solid; ^{1}H NMR (600 MHz, DMSO-d_6) δ 8.03 (s, 1H), 8.00 (d, *J* = 8.9 Hz, 1H), 7.82 (d, *J* = 8.9 Hz, 1H), 4.49 (q, *J* = 7.2 Hz, 2H), 3.89 (s, 3H), 1.40 (t, *J* = 7.2 Hz, 3H); HRMS (ESI) *m/z* calcd for $C_{11}H_{12}IN_2O_2$ $[M + H]^+$ 330.9937, found 330.9928.

Step 2: *N*-(3-Acetamido-4-methylphenyl)-3-(6-aminopyridin-3-yl)-1-ethyl-1*H*-indazole-5-carboxamide (18)

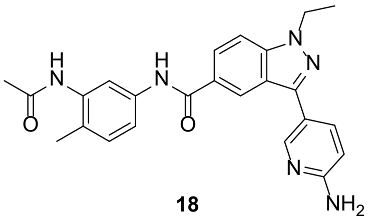

18

Chemical Formula: $C_{24}H_{24}N_6O_2$
Exact Mass: 428.1961

The general procedure employed for the Suzuki-Miyaura reaction was followed using methyl 1-ethyl-3-iodo-1*H*-indazole-5-carboxylate (17 mg, 0.05 mmol, 1.0 equiv.), (6-aminopyridin-3-yl)boronic acid (10.5 mg, 0.075 mmol, 1.5 equiv.) and Pd(dppf)Cl_2·CH_2Cl_2 (4 mg, 0.005 mmol, 0.1 equiv.) in DME/$Na_2CO_{3(sat)}$ (0.4 mL, *v/v* = 1:1). To the solution of crude cross-coupling product in THF/water (2 mL, *v/v* = 1:1) was added LiOH·H_2O (6 mg, 0.15 mmol, 3.0 equiv.), and the mixture was vigorously stirred at 45 °C for 16 h. The reaction mixture was neutralized to pH 7 with 4N $HCl_{(aq)}$ and concentrated *in vacuo*. To the solution of crude hydrolyzed product, *N*-(5-amino-2-methylphenyl)acetamide (12 mg, 0.075 mmol, 1.5 equiv.) and HATU (23 mg, 0.06 mmol, 1.2 equiv.) in anhydrous DMF (0.2 mL) was added DIEA (26 µL, 0.15 mmol, 3.0 equiv.) under nitrogen. The reaction mixture was stirred at room temperature for 16 h and then quenched by the addition of water. The aqueous layer was extracted twice with EtOAc, and the combined organic extracts were washed with brine, dried

over anhydrous Na_2SO_4, filtered and concentrated. The residue was purified by column chromatography on silica gel (CH_3OH/CH_2Cl_2, 3:97 to 5:95) to afford title compound **18** (17 mg, 77%) as a yellow foam; ^1H NMR (600 MHz, DMSO-d_6) δ 10.30 (s, 1H), 9.32 (s, 1H), 8.62 (s, 2H), 8.01−7.99 (m, 2H), 7.85 (s, 1H), 7.78 (d, J = 8.8 Hz, 1H), 7.54 (d, J = 8.2 Hz, 1H), 7.17 (d, J = 8.2 Hz, 1H), 6.62 (d, J = 8.5 Hz, 1H), 6.22 (s, 2H), 4.50 (q, J = 7.2 Hz, 2H), 2.18 (s, 3H), 2.07 (s, 3H), 1.44 (t, J = 7.2 Hz, 3H); ^{13}C NMR (150 MHz, DMSO-d_6) 168.2, 165.4, 159.6, 146.4, 142.6, 141.3, 137.2, 136.4, 135.8, 130.0, 127.4, 126.7, 126.0, 121.3, 119.9, 117.5, 117.5, 117.3, 109.7, 108.1, 43.2, 23.3, 17.4, 14.9; HRMS (ESI) *m/z* calcd for $C_{24}H_{25}N_6O_2$ $[M + H]^+$ 429.2033, found 429.2024.

4h) Synthesis of compound 19:

Step 1: Methyl 3-iodo-1-isopropyl-1*H*-indazole-5-carboxylate

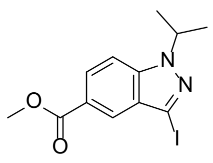

Chemical Formula: $C_{12}H_{13}IN_2O_2$
Exact Mass: 344.0022

To a solution of methyl 3-iodo-1*H*-indazole-5-carboxylate (91 mg, 0.3 mmol, 1.0 equiv.) and *t*-BuOK (40 mg, 0.36 mmol, 1.2 equiv.) in anhydrous THF (1 mL) was added 2-bromopropane (40 μL, 0.36 mmol, 1.2 equiv.) at 0 °C under nitrogen. The reaction mixture was allowed to warm up to room temperature and stirred for 16 h. After being quenched by the addition of water, the mixture was extracted twice with EtOAc, and the combined organic extracts were washed with brine, dried over anhydrous Na_2SO_4, filtered and concentrated. The residue was purified by column chromatography on silica gel (EtOAc/hexane, 0:100 to 5:95) to afford methyl 3-iodo-1-isopropyl-1*H*-indazole-5-carboxylate (56 mg, 54%) as a white solid; ^1H NMR (600 MHz, DMSO-d_6) δ 8.04 (s, 1H), 8.00 (d, J = 8.9 Hz, 1H), 7.84 (d, J = 8.9 Hz, 1H), 5.06 (h, J = 6.6 Hz, 1H), 3.89 (s, 3H), 1.48 (d, J = 6.6 Hz, 6H); HRMS (ESI) *m/z* calcd for $C_{12}H_{14}IN_2O_2$ $[M + H]^+$ 345.0094, found 345.009.

Step 2: *N*-(3-Acetamido-4-methylphenyl)-3-(6-aminopyridin-3-yl)-1-isopropyl-1*H*-indazole-5-carboxamide (19)

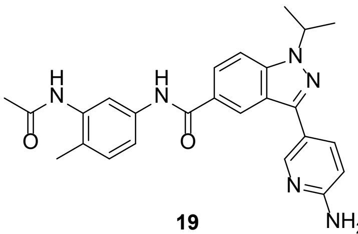

19

Chemical Formula: $C_{25}H_{26}N_6O_2$
Exact Mass: 442.2117

The general procedure employed for the Suzuki-Miyaura reaction was followed using methyl 3-iodo-1-isopropyl-1*H*-indazole-5-carboxylate (18 mg, 0.05 mmol, 1.0 equiv.), (6-aminopyridin-3-yl)boronic acid (10.5 mg, 0.075 mmol, 1.5 equiv.) and $Pd(dppf)Cl_2 \cdot CH_2Cl_2$ (4 mg, 0.005 mmol, 0.1 equiv.) in DME/$Na_2CO_{3(sat)}$ (0.4 mL, *v/v* = 1:1). To the solution of crude cross-coupling product in THF/water (2 mL, *v/v* = 1:1) was added $LiOH \cdot H_2O$ (6 mg, 0.15 mmol, 3.0 equiv.), and the mixture was vigorously stirred at 45 °C for 16 h. The resulting mixture was neutralized to pH 7 with 4N $HCl_{(aq)}$ and concentrated *in vacuo*. To the solution of crude hydrolyzed product, *N*-(5-amino-2-methylphenyl)acetamide (12 mg, 0.075 mmol, 1.5 equiv.) and HATU (23 mg, 0.06 mmol, 1.2 equiv.) in anhydrous DMF (0.2 mL) was added DIEA (26 μL, 0.15 mmol, 3.0 equiv.) under nitrogen. The reaction mixture was stirred at room temperature for 16 h and then quenched by the addition of water. The aqueous layer was extracted twice with EtOAc, and the combined organic extracts were washed with brine, dried over anhydrous Na_2SO_4, filtered and concentrated. The residue was purified by column chromatography on silica gel (CH_3OH/CH_2Cl_2, 3:97 to 5:95) to afford title compound **19** (17 mg, 81%) as a yellow foam; ^1H NMR (600 MHz, DMSO-d_6) δ 10.30 (s, 1H), 9.32 (s, 1H), 8.62 (s, 2H), 8.00−7.98 (m, 2H), 7.85 (s, 1H), 7.81 (d, J = 8.9 Hz, 1H), 7.55 (d, J = 8.2 Hz, 1H), 7.17 (d, J = 8.2 Hz, 1H), 6.63 (d, J = 8.5 Hz, 1H), 6.22 (s, 2H), 5.08−5.02 (m, 1H), 2.18 (s, 3H), 2.07 (s, 3H), 1.54 (s, 3H), 1.53 (s, 3H); ^{13}C NMR (150 MHz, DMSO-d_6) 168.2, 165.4, 159.6, 146.4, 142.4, 140.8, 137.2, 136.4, 135.9,

130.0, 127.4, 126.7, 125.8, 121.3, 119.9, 117.5, 117.5, 117.5, 109.7, 108.1, 49.7, 23.3, 22.0 (2 ×), 17.4; HRMS (ESI) *m/z* calcd for $C_{25}H_{27}N_6O_2$ [M + H]$^+$ 443.2190, found 443.2179.

4i) Synthesis of compound 21:

Step 1: *N*-(3-Acetamidophenyl)-3-iodo-1-methyl-1*H*-indazole-5-carboxamide

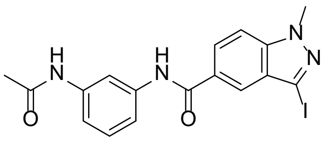

Chemical Formula: $C_{17}H_{15}IN_4O_2$
Exact Mass: 434.0240

To a solution of 3-iodo-1-methyl-1*H*-indazole-5-carboxylic acid (31 mg, 0.1 mmol, 1.0 equiv.), *N*-(3-aminophenyl)acetamide (23 mg, 0.15 mmol, 1.5 equiv.) and HATU (46 mg, 0.12 mmol, 1.2 equiv.) in anhydrous DMF (0.2 mL) was added DIEA (35 µL, 0.2 mmol, 2.0 equiv.) under nitrogen. The resulting mixture was stirred at room temperature for 16 h and then quenched by the addition of water. The precipitate was collected, sequentially washed with water and ether and dried *in vacuo* to afford *N*-(3-acetamidophenyl)-3-iodo-1-methyl-1*H*-indazole-5-carboxamide (37 mg, 85%) as a light brown solid, which was directly used in the next step without further purification; ^{1}H NMR (600 MHz, DMSO-*d*$_6$) δ 10.41 (s, 1H), 9.99 (s, 1H), 8.15 (s, 1H), 8.12 (s, 1H), 8.07 (d, *J* = 8.7 Hz, 1H), 7.78 (d, *J* = 8.7 Hz, 1H), 7.45 (d, *J* = 7.4 Hz, 1H), 7.33 (d, *J* = 7.4 Hz, 1H), 7.26 (t, *J* = 7.4 Hz, 1H), 4.12 (s, 3H), 2.05 (s, 3H); HRMS (ESI) *m/z* calcd for $C_{17}H_{16}N_4O_2$ [M + H]$^+$ 435.0312, found 435.0305.

Step 2: *N*-(3-Acetamidophenyl)-3-(4-amino-2-methylphenyl)-1-methyl-1*H*-indazole-5-carboxamide (21)

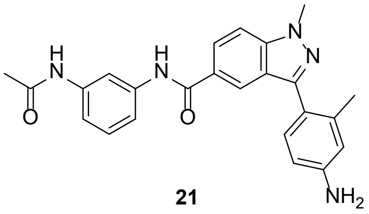

21

Chemical Formula: $C_{24}H_{23}N_5O_2$
Exact Mass: 413.1852

The general procedure employed for the Suzuki-Miyaura reaction was followed using *N*-(3-acetamidophenyl)-3-iodo-1-methyl-1*H*-indazole-5-carboxamide (22 mg, 0.05 mmol, 1.0 equiv.), 3-methyl-4-(4,4,5,5-tetramethyl-1,3,2-dioxaborolan-2-yl)aniline (19 mg, 0.075 mmol, 1.5 equiv.) and Pd(dppf)Cl_2·CH_2Cl_2 (4 mg, 0.005 mmol, 0.1 equiv.) in DME/$Na_2CO_{3(sat)}$ (0.4 mL, *v/v* = 1:1). Isolation and purification afforded title compound **21** (8.5 mg, 41%) as a pale yellow solid; ^{1}H NMR (600 MHz, DMSO-*d*$_6$) δ 10.28 (s, 1H), 9.96 (s, 1H), 8.29 (s, 1H), 8.06 (s, 1H), 8.00 (d, *J* = 8.8 Hz, 1H), 7.73 (d, *J* = 8.8 Hz, 1H), 7.42 (d, *J* = 8.2 Hz, 1H), 7.32 (d, *J* = 8.2 Hz, 1H), 7.24−7.21 (m, 2H), 6.57−6.54 (m, 2H), 5.25 (s, 2H), 4.10 (s, 3H), 2.25 (s, 3H), 2.04 (s, 3H); ^{13}C NMR (150 MHz, DMSO-*d*$_6$) δ 172.2, 168.5, 149.2, 147.1, 142.9, 139.9, 139.7, 138.9, 132.6, 130.6, 128.1, 127.2, 123.1, 123.1, 121.1, 118.7, 118.4, 117.7, 114.6, 114.1, 111.3, 36.4, 24.7, 21.3; HRMS (ESI) *m/z* calcd for $C_{24}H_{24}N_5O_2$ [M + H]$^+$ 414.1924, found 414.1918.

4j) Synthesis of compound 22:

Step 1: 3-Iodo-1-methyl-*N*-(4-methyl-3-(methylcarbamoyl)phenyl)-1*H*-indazole-5-carboxamide

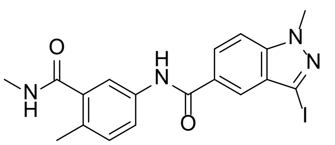

Chemical Formula: $C_{18}H_{17}IN_4O_2$
Exact Mass: 448.0396

To a solution of 3-iodo-1-methyl-1*H*-indazole-5-carboxylic acid (31 mg, 0.1 mmol, 1.0 equiv.), 5-amino-*N*,2-dimethylbenzamide (25 mg, 0.15 mmol, 1.5 equiv.) and HATU (46 mg, 0.12 mmol, 1.2 equiv.) in anhydrous DMF (0.2 mL) was added DIEA (35 µL, 0.2 mmol, 2.0 equiv.) under nitrogen. The resulting mixture was stirred at room temperature for 16 h and then quenched by the addition of water. The precipitate was collected, sequentially washed with water and ether and dried *in vacuo* to afford 3-iodo-1-methyl-*N*-(4-methyl-3-(methylcarbamoyl)phenyl)-1*H*-indazole-5-carboxamide (34 mg, 75%) as a pale yellow solid, which was

directly used in the next step without further purification; ^{1}H NMR (600 MHz, DMSO-d_6) δ 10.41 (s, 1H), 8.19 (d, J = 4.5 Hz, 1H), 8.16 (s, 1H), 8.07 (d, J = 8.9 Hz, 1H), 7.80−7.77 (m, 3H), 7.22 (d, J = 8.2 Hz, 1H), 4.12 (s, 3H), 2.76 (d, J = 4.5 Hz, 3H), 2.29 (s, 3H); HRMS (ESI) *m/z* calcd for $C_{18}H_{18}IN_4O_2$ [M + H]$^+$ 449.0468, found 449.0458.

Step 2: 3-(4-Amino-2-methylphenyl)-1-methyl-*N*-(4-methyl-3-(methylcarbamoyl)phenyl)-1*H*-indazole-5-carboxamide (22)

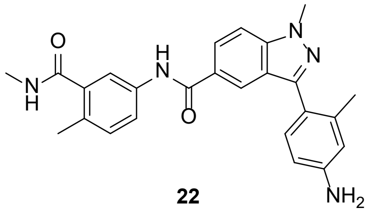

22

Chemical Formula: $C_{25}H_{25}N_5O_2$
Exact Mass: 427.2008

The general procedure employed for the Suzuki-Miyaura reaction was followed using 3-iodo-1-methyl-*N*-(4-methyl-3-(methylcarbamoyl)phenyl)-1*H*-indazole-5-carboxamide (22 mg, 0.05 mmol, 1.0 equiv.), 3-methyl-4-(4,4,5,5-tetramethyl-1,3,2-dioxaborolan-2-yl)aniline (19 mg, 0.075 mmol, 1.5 equiv.) and Pd(dppf)Cl_2·CH_2Cl_2 (4 mg, 0.005 mmol, 0.1 equiv.) in DME/$Na_2CO_{3(sat)}$ (0.4 mL, *v/v* = 1:1). Isolation and purification afforded title compound **22** (7 mg, 32%) as a pale yellow foam; ^{1}H NMR (600 MHz, DMSO-d_6) δ 10.28 (s, 1H), 8.29 (s, 1H), 8.16 (d, J = 4.5 Hz, 1H), 8.01 (d, J = 8.8 Hz, 1H), 7.75−7.73 (m, 3H), 7.22 (d, J = 8.1 Hz, 1H), 7.19 (d, J = 8.1 Hz, 1H), 6.58−6.55 (m, 2H), 5.25 (s, 2H), 4.10 (s, 3H), 2.74 (d, J = 4.5 Hz, 3H), 2.28 (s, 3H), 2.24 (s, 3H); ^{13}C NMR (150 MHz, DMSO-d_6) δ 169.4, 165.6, 148.8, 145.7, 141.5, 137.4, 137.0, 139.9, 131.1, 130.5, 129.9, 126.8, 125.6, 121.9, 121.5, 121.0, 119.1, 118.9, 115.9, 111.5, 109.6, 35.5, 25.9, 20.6, 18.8; HRMS (ESI) *m/z* calcd for $C_{25}H_{26}N_5O_2$ [M + H]$^+$ 428.2081, found 428.2076.

4k) Synthesis of compound 23:

Step 1: *N*-(2-Methoxy-5-nitrophenyl)benzamide

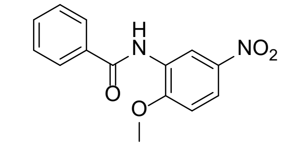

Chemical Formula: $C_{14}H_{12}N_2O_4$
Exact Mass: 272.0797

To a solution of 2-methoxy-5-nitroaniline (**1a**) (37 mg, 0.22 mmol, 1.0 equiv.) and TEA (46 μL, 0.33 mmol, 1.5 equiv.) in anhydrous CH_2Cl_2 (2 mL) was added benzoyl chloride (30 μL, 0.24 mmol, 1.1 equiv.) under nitrogen. The reaction mixture was stirred at room temperature for 16 h and then quenched by the addition of water. The aqueous layer was extracted twice with EtOAc, and the combined organic extracts were washed with brine, dried over anhydrous Na_2SO_4, filtered and concentrated. The residue was purified by column chromatography on silica gel (EtOAc/hexane, 20:80 to 40:60) to afford *N*-(2-methoxy-5-nitrophenyl)benzamide (60 mg, 99%) as a white solid; ^{1}H NMR (600 MHz, DMSO-d_6) δ 9.74 (s, 1H), 8.80 (d, J = 2.8 Hz, 1H), 8.14 (dd, J = 9.2, 2.8 Hz, 1H), 7.98 (d, J = 7.4 Hz, 2H), 7.63 (t, J = 7.4 Hz, 1H), 7.55 (t, J = 7.4 Hz, 2H), 7.33 (d, J = 9.2 Hz, 1H), 4.00 (s, 3H); HRMS (ESI) *m/z* calcd for $C_{14}H_{13}N_2O_4$ [M + H]$^+$ 273.0869, found 273.0865.

Step 2: *N*-(3-Benzamido-4-methoxyphenyl)-3-iodo-1-methyl-1*H*-indazole-5-carboxamide

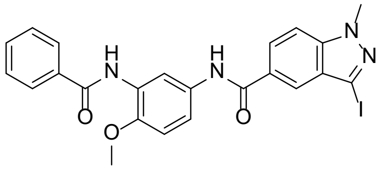

Chemical Formula: $C_{23}H_{19}IN_4O_3$
Exact Mass: 526.0502

To a solution of *N*-(2-methoxy-5-nitrophenyl)benzamide (60 mg, 0.22 mmol, 1.0 equiv.) and acetic acid (330 μL) in CH_3OH (3 mL) was added zinc powder (143 mg, 2.2 mmol, 10.0 equiv.) at 0 ºC with vigorous stirring. The reaction mixture was allowed to warm up to room temperature and stirred for 1 h. The excess zinc powder was then filtered off through a pad of Celite, and the resulting solution was neutralized to pH 7 with 4M $NaOH_{(aq)}$. The residue was concentrated *in vacuo* and purified by reverse-phase column chromatography on C-18 (CH_3CN/water, 5:95 to 100:0) to afford amine as a light yellow solid. To the solution of the amine, 3-iodo-1-methyl-1*H*-indazole-5-carboxylic acid (72

mg, 0.24 mmol, 1.1 equiv.) and HATU (125 mg, 0.33 mmol, 1.5 equiv.) in anhydrous DMF (0.5 mL) was added DIEA (57 µL, 0.33 mmol, 1.5 equiv.) under nitrogen. The reaction mixture was stirred at room temperature for 16 h and then quenched by the addition of water. The aqueous layer was extracted twice with EtOAc, and the combined organic extracts were washed with brine, dried over anhydrous Na_2SO_4, filtered and concentrated. The residue was purified by column chromatography on silica gel (CH_3OH/CH_2Cl_2, 1:99 to 2:98) to afford *N*-(3-benzamido-4-methoxyphenyl)-3-iodo-1-methyl-1*H*-indazole-5-carboxamide (84 mg, 72%) as a pale yellow foam; ^{1}H NMR (600 MHz, DMSO-d_6) δ 10.36 (s, 1H), 9.49 (s, 1H), 8.24 (d, J = 2.3 Hz, 1H), 8.18 (s, 1H), 8.09 (d, J = 8.8 Hz, 1H), 7.98 (d, J = 7.4 Hz, 2H), 7.79 (d, J = 8.8 Hz, 1H), 7.68 (dd, J = 8.9, 2.3 Hz, 1H), 7.60 (t, J = 7.4 Hz, 1H), 7.54 (t, J = 7.4 Hz, 2H), 7.11 (d, J = 8.9 Hz, 1H), 4.12 (s, 3H), 3.85 (s, 3H); HRMS (ESI) *m/z* calcd for $C_{23}H_{20}IN_4O_3$ $[M + H]^+$ 527.0574, found 527.0573.

Step 3: 3-(4-Amino-2-methylphenyl)-*N*-(3-benzamido-4-methoxyphenyl)-1-methyl-1*H*-indazole-5-carboxamide (23)

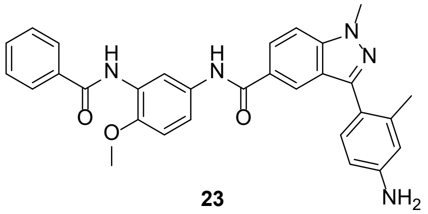

23

Chemical Formula: $C_{30}H_{27}N_5O_3$
Exact Mass: 505.2114

The general procedure employed for the Suzuki-Miyaura reaction was followed using *N*-(3-benzamido-4-methoxyphenyl)-3-iodo-1-methyl-1*H*-indazole-5-carboxamide (42 mg, 0.08 mmol, 1.0 equiv.), 3-methyl-4-(4,4,5,5-tetramethyl-1,3,2-dioxaborolan-2-yl)aniline (28 mg, 0.12 mmol, 1.5 equiv.) and $Pd(dppf)Cl_2{\cdot}CH_2Cl_2$ (6.5 mg, 0.008 mmol, 0.1 equiv.) in DME/$Na_2CO_{3(sat)}$ (0.4 mL, *v/v* = 1:1) at 110 °C for 2 h. Isolation and purification afforded title compound **23** (29 mg, 57%) as a pale yellow foam; ^{1}H NMR (600 MHz, DMSO-d_6) δ 10.24 (s, 1H), 9.47 (s, 1H), 8.32 (s, 1H), 8.19 (d, J = 2.3 Hz, 1H), 8.03 (d, J = 8.8 Hz, 1H), 7.97 (d, J = 7.4 Hz, 2H), 7.73 (d, J = 8.8 Hz, 1H), 7.65 (dd, J = 9.0, 2.3 Hz, 1H), 7.60 (t, J = 7.4 Hz, 1H), 7.53 (t, J = 7.4 Hz, 2H), 7.23 (d, J = 8.0 Hz, 1H), 7.08 (d, J = 9.0 Hz, 1H), 6.58−6.55 (m, 2H), 5.25 (s, 2H), 4.10 (s, 3H), 3.83 (s, 3H), 2.25 (s, 3H); ^{13}C NMR (150 MHz, DMSO-d_6) δ 165.3, 164.9, 148.8, 147.8, 145.6, 141.5, 137.0, 134.5, 132.2, 131.7, 131.1, 128.5 (2 ×), 127.5 (2 ×), 127.0, 126.4, 125.6, 121.9, 121.4, 119.0, 118.1, 117.5, 115.9, 111.5, 111.2, 109.5, 56.0, 35.5, 20.6; HRMS (ESI) *m/z* calcd for $C_{30}H_{28}N_5O_3$ $[M + H]^+$ 506.2186, found 506.2179.

4l) Synthesis of compound 24:

Step 1: 3-Iodo-*N*-(4-methoxyphenyl)-1-methyl-1*H*-indazole-5-carboxamide

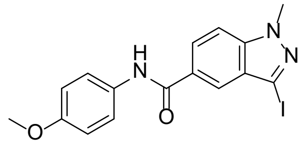

Chemical Formula: $C_{16}H_{14}IN_3O_2$
Exact Mass: 407.0131

To a solution of 3-iodo-1-methyl-1*H*-indazole-5-carboxylic acid (60 mg, 0.2 mmol, 1.0 equiv.), 4-methoxyaniline (37 mg, 0.3 mmol, 1.5 equiv.) and HATU (91 mg, 0.24 mmol, 1.2 equiv.) in anhydrous DMF (0.2 mL) was added DIEA (42 µL, 0.24 mmol, 1.2 equiv.) under nitrogen. The reaction mixture was stirred at room temperature for 16 h and then quenched by the addition of water. The precipitate was collected, sequentially washed with water and ether and dried *in vacuo* to afford 3-iodo-*N*-(4-methoxyphenyl)-1-methyl-1*H*-indazole-5-carboxamide (77 mg, 95%) as a pale yellow solid, which was directly used in the next step without further purification; ^{1}H NMR (600 MHz, DMSO-d_6) δ 10.28 (s, 1H), 8.14 (s, 1H), 8.07 (d, J = 8.8 Hz, 1H), 7.78 (d, J = 8.8 Hz, 1H), 7.69 (d, J = 9.0 Hz, 2H), 6.94 (d, J = 9.0 Hz, 2H), 4.11 (s, 3H), 3.75 (s, 3H); HRMS (ESI) *m/z* calcd for $C_{16}H_{15}IN_3O_2$ $[M + H]^+$ 408.0203, found 408.0191.

Step 2: 3-(4-Amino-2-methylphenyl)-*N*-(4-methoxyphenyl)-1-methyl-1*H*-indazole-5-carboxamide (24)

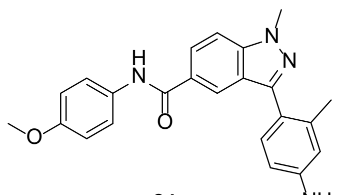

24

Chemical Formula: $C_{23}H_{22}N_4O_2$
Exact Mass: 386.1743

The general procedure employed for the Suzuki-Miyaura reaction was followed using 3-iodo-*N*-(4-methoxyphenyl)-1-methyl-1*H*-indazole-5-carboxamide (21 mg, 0.05 mmol, 1.0 equiv.), 3-methyl-4-(4,4,5,5-tetramethyl-1,3,2-dioxaborolan-2-yl)aniline (19 mg, 0.075 mmol, 1.5 equiv.) and Pd(dppf)Cl_2·CH_2Cl_2 (4 mg, 0.005 mmol, 0.1 equiv.) in DME/$Na_2CO_{3(sat)}$ (0.4 mL, *v/v* = 1:1) at 100 ºC for 1 h. Isolation and purification afforded title compound **24** (6 mg, 31%) as a pale yellow foam; ^{1}H NMR (600 MHz, DMSO-d_6) δ 10.15 (s, 1H), 8.27 (s, 1H), 8.00 (d, *J* = 8.8 Hz, 1H), 7.72 (d, *J* = 8.8 Hz, 1H), 7.65 (d, *J* = 9.0 Hz, 2H), 7.21 (d, *J* = 8.1 Hz, 1H), 6.91 (d, *J* = 9.0 Hz, 2H), 6.57−6.54 (m, 2H), 5.25 (s, 2H), 4.10 (s, 3H), 3.74 (s, 3H), 2.24 (s, 3H); ^{13}C NMR (150 MHz, DMSO-d_6) δ 165.3, 155.4, 148.8, 145.6, 141.4, 137.0, 132.4, 131.0, 127.1, 125.6, 122.1 (2 ×), 121.9, 121.3, 119.0, 115.9, 113.7 (2 ×), 111.5, 109.5, 55.2, 35.5, 20.6; HRMS (ESI) *m/z* calcd for $C_{23}H_{23}N_4O_2$ $[M + H]^+$ 387.1815, found 387.1808.

4m) Synthesis of compound 25:

Step 1: 3-Iodo-1-methyl-*N*-phenyl-1*H*-indazole-5-carboxamide

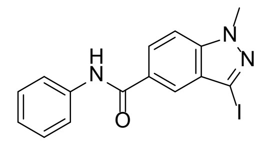

Chemical Formula: $C_{15}H_{12}IN_3O$
Exact Mass: 377.0025

To a solution of 3-iodo-1-methyl-1*H*-indazole-5-carboxylic acid (60 mg, 0.2 mmol, 1.0 equiv.), aniline (28 mg, 0.3 mmol, 1.5 equiv.) and HATU (91 mg, 0.24 mmol, 1.2 equiv.) in anhydrous DMF (0.5 mL) was added DIEA (42 μL, 0.24 mmol, 1.2 equiv.) under nitrogen. The reaction mixture was stirred at room temperature for 16 h and then quenched by the addition of water. The aqueous layer was extracted twice with EtOAc, and the combined organic extracts were washed with brine, dried over anhydrous Na_2SO_4, filtered and concentrated. The residue was purified by column chromatography on silica gel (CH_3OH/CH_2Cl_2, 1:99 to 5:95) to afford 3-iodo-1-methyl-*N*-phenyl-1*H*-indazole-5-carboxamide (64 mg, 85%) as a pale yellow solid; ^{1}H NMR (600 MHz, DMSO-d_6) δ 10.39 (s, 1H), 8.15 (d, *J* = 1.6 Hz, 1H), 8.07 (dd, *J* = 8.8, 1.6 Hz, 1H), 7.81−7.79 (m, 3H), 7.37 (dd, *J* = 8.4, 7.4 Hz, 2H), 7.11 (t, *J* = 7.4 Hz, 1H), 4.12 (s, 3H); HRMS (ESI) *m/z* calcd for $C_{15}H_{13}IN_3O$ $[M + H]^+$ 378.0097, found 378.0090.

Step 2: 3-(4-Amino-2-methylphenyl)-1-methyl-*N*-phenyl-1*H*-indazole-5-carboxamide (25)

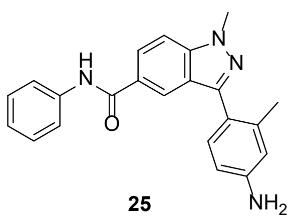

25

Chemical Formula: $C_{22}H_{20}N_4O$
Exact Mass: 356.1637

The general procedure employed for the Suzuki-Miyaura reaction was followed using 3-iodo-1-methyl-*N*-phenyl-1*H*-indazole-5-carboxamide (38 mg, 0.1 mmol, 1.0 equiv.), 3-methyl-4-(4,4,5,5-tetramethyl-1,3,2-dioxaborolan-2-yl)aniline (35 mg, 0.15 mmol, 1.5 equiv.) and Pd(dppf)Cl_2·CH_2Cl_2 (7.5 mg, 0.01 mmol, 0.1 equiv.) in DME/$Na_2CO_{3(sat)}$ (0.4 mL, *v/v* = 1:1) at 90 ºC for 3 h. Isolation and purification afforded title compound **25** (15 mg, 42%) as a pale yellow foam; ^{1}H NMR (600 MHz, DMSO-d_6) δ 10.27 (s, 1H), 8.29 (s, 1H), 8.01 (d, *J* = 8.8 Hz, 1H), 7.76 (d, *J* = 7.9 Hz, 2H), 7.74 (d, *J* = 8.8 Hz, 1H), 7.34 (t, *J* = 7.8 Hz, 2H), 7.22 (d, *J* = 8.1 Hz, 1H), 7.08 (t, *J* = 7.8 Hz, 1H), 6.58 (s, 1H), 6.56 (d, *J* = 8.1 Hz, 1H), 5.25 (s, 2H), 4.10 (s, 3H), 2.25 (s, 3H); ^{13}C NMR (150 MHz, DMSO-d_6) δ 165.7, 148.8, 145.6, 141.5, 139.4, 137.0, 131.1, 128.5 (2 ×), 127.0, 125.7, 123.5, 121.9, 121.5, 120.5 (2 ×), 118.9, 115.9, 111.5, 109.5, 35.5, 20.6; HRMS (ESI) *m/z* calcd for $C_{22}H_{21}N_4O$ $[M + H]^+$ 357.1709, found 357.1702.

4n) Synthesis of compound 26:

Step 1: 3-Iodo-*N*-(4-methoxyphenyl)-*N*,1-dimethyl-1*H*-indazole-5-carboxamide

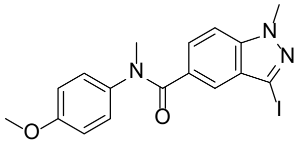

Chemical Formula: $C_{17}H_{16}IN_3O_2$
Exact Mass: 421.0287

To a solution of 3-iodo-1-methyl-1*H*-indazole-5-carboxylic acid (60 mg, 0.2 mmol, 1.0 equiv.), 4-methoxy-*N*-methylaniline (42 mg, 0.3 mmol, 1.5 equiv.) and HATU (91 mg, 0.24 mmol, 1.2 equiv.) in anhydrous DMF (0.5 mL) was added DIEA (42 μL, 0.24 mmol, 1.2 equiv.) under nitrogen. The reaction mixture was stirred at room temperature for 16 h and then quenched by the addition of water. The aqueous layer was extracted twice with EtOAc, and the combined organic extracts were washed with brine, dried over anhydrous Na_2SO_4, filtered and concentrated. The residue was purified by column chromatography on silica gel (CH_3OH/CH_2Cl_2, 1:99 to 2:98) to afford 3-iodo-*N*-(4-methoxyphenyl)-*N*,1-dimethyl-1*H*-indazole-5-carboxamide (80 mg, 95%) as a light yellow oil; ^{1}H NMR (600 MHz, DMSO-d_6) δ 7.47 (d, *J* = 8.7 Hz, 1H), 7.35−7.32 (m, 2H), 7.12 (d, *J* = 8.8 Hz, 2H), 6.80 (d, *J* = 8.8 Hz, 2H), 4.00 (s, 3H), 3.66 (s, 3H), 3.36 (s, 3H); HRMS (ESI) *m/z* calcd for $C_{17}H_{17}IN_3O_2$ $[M + H]^+$ 422.0359, found 422.0352.

Step 2: 3-(4-Amino-2-methylphenyl)-*N*-(4-methoxyphenyl)-*N*,1-dimethyl-1*H*-indazole-5-carboxamide (26)

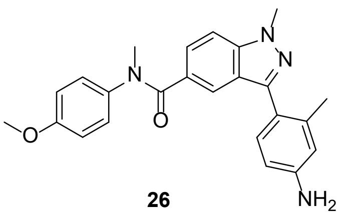

26

Chemical Formula: $C_{24}H_{24}N_4O_2$
Exact Mass: 400.1899

The general procedure employed for the Suzuki-Miyaura reaction was followed using 3-iodo-*N*-(4-methoxyphenyl)-*N*,1-dimethyl-1*H*-indazole-5-carboxamide (22 mg, 0.05 mmol, 1.0 equiv.), 3-methyl-4-(4,4,5,5-tetramethyl-1,3,2-dioxaborolan-2-yl)aniline (18 mg, 0.075 mmol, 1.5 equiv.) and $Pd(dppf)Cl_2·CH_2Cl_2$ (4 mg, 0.005 mmol, 0.1 equiv.) in DME/$Na_2CO_{3(sat)}$ (0.4 mL, *v/v* = 1:1) at 110 ºC for 2 h. Isolation and purification afforded title compound **26** (17 mg, 85%) as a pale yellow oil; ^{1}H NMR (600 MHz, DMSO-d_6) δ 7.48 (d, *J* = 8.7 Hz, 1H), 7.41 (d, *J* = 8.7 Hz, 1H), 7.38 (s, 1H), 7.10 (d, *J* = 8.5 Hz, 2H), 6.83 (d, *J* = 8.5 Hz, 2H), 6.69 (d, *J* = 7.9 Hz, 1H), 6.50 (s, 1H), 6.45 (d, *J* = 7.9 Hz, 1H), 5.22 (s, 2H), 3.99 (s, 3H), 3.69 (s, 3H), 3.34 (s, 3H), 2.11 (s, 3H); ^{13}C NMR (150 MHz, DMSO-d_6) δ 169.4, 157.4, 148.5, 144.9, 140.1, 138.2, 136.7, 130.7, 128.2 (2 ×), 127.7, 126.7, 122.6, 121.0, 118.7, 115.9, 114.3 (2 ×), 111.3, 109.0, 55.3, 38.3, 35.3, 20.4; HRMS (ESI) *m/z* calcd for $C_{24}H_{25}N_4O_2$ $[M + H]^+$ 401.1972, found 401.1965.

4o) Synthesis of compound 27:

Step 1: Methyl 3-bromo-2-methyl-2*H*-indazole-5-carboxylate

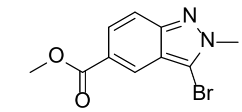

Chemical Formula: $C_{10}H_9BrN_2O_2$
Exact Mass: 267.9847

To a solution of methyl 2-methyl-2*H*-indazole-5-carboxylate (190 mg, 1 mmol, 1.0 equiv.) in anhydrous DMF (3 mL) was added NBS (267 mg, 1.5 mmol, 1.5 equiv.) at 0 ºC under nitrogen. The reaction mixture was allowed to warm up to room temperature and stirred for 16 h. Saturated aqueous $NaHCO_3$ was added and the aqueous layer was extracted twice with EtOAc. The combined organic extracts were washed with brine, dried over anhydrous Na_2SO_4, filtered and concentrated. The residue was purified by column chromatography on silica gel (EtOAc/hexane, 30:70) to afford methyl 3-bromo-2-methyl-2*H*-indazole-5-carboxylate (221 mg, 82%) as a white solid; ^{1}H NMR (600 MHz, DMSO-d_6) δ 8.18 (s, 1H), 7.79 (d, *J* = 9.1 Hz, 1H), 7.70 (d, *J* = 9.1 Hz, 1H), 4.18 (s, 3H), 3.87 (s, 3H); HRMS (ESI) *m/z* calcd for $C_{10}H_{10}BrN_2O_2$ $[M + H]^+$ 268.9920, found 268.9918.

Step 2: 3-(4-((*tert*-Butoxycarbonyl)amino)-2-methylphenyl)-2-methyl-2*H*-indazole-5-carboxylic acid

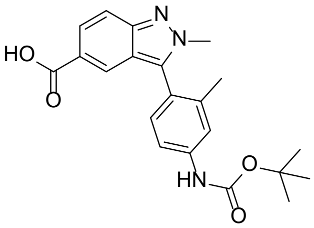

Chemical Formula: $C_{21}H_{23}N_3O_4$
Exact Mass: 381.1689

The general procedure employed for the Suzuki-Miyaura reaction was followed using methyl 3-bromo-2-methyl-2*H*-indazole-5-carboxylate (68 mg, 0.25 mmol, 1.0 equiv.), *tert*-butyl (3-methyl-4-(4,4,5,5-tetramethyl-1,3,2-dioxaborolan-2-yl)phenyl)carbamate (125 mg, 0.375 mmol, 1.5 equiv.) and Pd(dppf)Cl_2·CH_2Cl_2 (20 mg, 0.025 mmol, 0.1 equiv.) in DME/$Na_2CO_{3(sat)}$ (4 mL, *v/v* = 1:1). To the solution of crude cross-coupling product in THF/water (1.5 mL, *v/v* = 2:1) was added LiOH·H_2O (22 mg, 0.5 mmol, 2.0 equiv.), and the mixture was vigorously stirred at 45 °C for 16 h. The resulting mixture was neutralized to pH 7 with 4N $HCl_{(aq)}$ and concentrated *in vacuo*. The residue was purified by reverse-phase column chromatography on C-18 (CH_3CN/water, 5:95 to 100:0) to afford 3-(4-((*tert*-butoxycarbonyl)amino)-2-methylphenyl)-2-methyl-2*H*-indazole-5-carboxylic acid (91 mg, 95%) as a white foam; ^{1}H NMR (600 MHz, DMSO-*d*$_6$) δ 9.61 (s, 1H), 7.87−7.86 (m, 2H), 7.61 (s, 1H), 7.44−7.42 (m, 2H), 7.23 (d, *J* = 8.3 Hz, 1H), 3.88 (s, 3H), 2.01 (s, 3H), 1.50 (s, 9H); ^{13}C NMR (150 MHz, DMSO-*d*$_6$) δ 169.8, 152.8, 147.8, 140.6, 138.0, 135.8, 133.4, 131.2, 127.8, 122.3, 121.3, 120.9, 119.5, 115.8, 114.6, 79.3, 37.7, 28.1 (3 ×), 19.9; HRMS (ESI) *m/z* calcd for $C_{21}H_{24}N_3O_4$ [M + H]$^+$ 382.1761, found 382.1751.

Step 3: 3-(4-Amino-2-methylphenyl)-*N*-(4-methoxyphenyl)-2-methyl-2*H*-indazole-5-carboxamide (27)

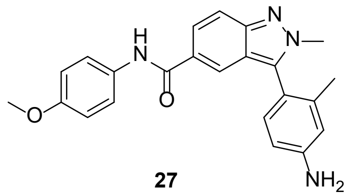

27

Chemical Formula: $C_{23}H_{22}N_4O_2$
Exact Mass: 386.1743

To a solution of 3-(4-((*tert*-butoxycarbonyl)amino)-2-methylphenyl)-2-methyl-2*H*-indazole-5-carboxylic acid (38 mg, 0.1 mmol, 1.0 equiv.), 4-methoxyaniline (13 mg, 0.1 mmol, 1.0 equiv.) and HATU (46 mg, 0.12 mmol, 1.2 equiv.) in anhydrous DMF (0.2 mL) was added DIEA (26 µL, 0.15 mmol, 1.5 equiv.) under nitrogen. The reaction mixture was stirred at room temperature for 16 h and then quenched by the addition of water. The aqueous layer was extracted twice with EtOAc, and the combined organic extracts were washed with brine, dried over anhydrous Na_2SO_4, filtered and concentrated. The crude amide-coupling product was directly treated with a 4N HCl solution in 1,4-dioxane (1 mL), and the mixture was stirred at room temperature for 1 h. The resulting mixture was neutralized to pH 7 with 4M $NaOH_{(aq)}$ and concentrated *in vacuo*. The residue was purified by reverse-phase column chromatography on C-18 (CH_3CN/water, 5:95 to 100:0) to afford title compound **27** (26 mg, 67%) as a white foam; ^{1}H NMR (600 MHz, DMSO-*d*$_6$) δ 10.06 (s, 1H), 8.06 (s, 1H), 7.82 (d, *J* = 9.1 Hz, 1H), 7.65 (d, *J* = 9.1 Hz, 1H), 7.63 (d, *J* = 9.0 Hz, 2H), 7.03 (d, *J* = 8.2 Hz, 1H), 6.89 (d, *J* = 9.0 Hz, 2H), 6.63 (d, *J* = 1.7 Hz, 1H), 6.58 (dd, *J* = 8.2, 1.7 Hz, 1H), 5.45 (s, 2H), 3.92 (s, 3H), 3.73 (s, 3H), 1.94 (s, 3H); ^{13}C NMR (150 MHz, DMSO-*d*$_6$) δ 165.3, 155.4, 150.1, 147.9, 138.4, 138.1, 132.4, 131.6, 127.2, 124.9, 122.2 (2 ×), 121.4, 120.5, 116.5, 115.2, 114.5, 113.6 (2 ×), 111.6, 55.2, 37.9, 19.8; HRMS (ESI) *m/z* calcd for $C_{23}H_{23}N_4O_2$ [M + H]$^+$ 387.1815, found 387.1807.

4p) Syntheses of compounds 28−36:

Step 1: 1-Methyl-3-(1-methyl-1*H*-indol-2-yl)-1*H*-indazole-5-carboxylic acid

The general procedure employed for the Suzuki-Miyaura reaction was followed using 3-iodo-1-methyl-1*H*-indazole-5-carboxylic acid (362 mg, 1.2 mmol, 1.0 equiv.), 1-methyl-2-(4,4,5,5-tetramethyl-1,3,2-dioxaborolan-2-yl)-1*H*-indole (618 mg, 2.4 mmol, 2.0 equiv.) and Pd(dppf)Cl_2·CH_2Cl_2 (100 mg, 0.12

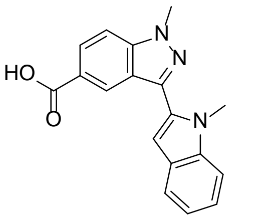

Chemical Formula: $C_{18}H_{15}N_3O_2$
Exact Mass: 305.1164

mmol, 0.1 equiv.) in DME/$Na_2CO_{3(sat)}$ (4 mL, *v/v* = 1:1). Isolation and purification afforded 1-methyl-3-(1-methyl-1*H*-indol-2-yl)-1*H*-indazole-5-carboxylic acid (300 mg, 80%) as a light yellow solid; ^{1}H NMR (600 MHz, DMSO-d_6) δ 12.98 (br s, 1H), 8.59 (s, 1H), 8.04 (d, *J* = 8.8 Hz, 1H), 7.81 (d, *J* = 8.8 Hz, 1H), 7.70 (d, *J* = 7.8 Hz, 1H), 7.55 (d, *J* = 7.8 Hz, 1H), 7.25 (t, *J* = 7.8 Hz, 1H), 7.12 (t, *J* = 7.8 Hz, 1H), 7.04 (s, 1H), 4.19 (s, 3H), 4.05 (s, 3H); HRMS (ESI) *m/z* calcd for $C_{18}H_{16}N_3O_2$ [M + H]$^+$ 306.1237, found 306.1228.

Step 2a: *N*-(4-Methoxyphenyl)-1-methyl-3-(1-methyl-1*H*-indol-2-yl)-1*H*-indazole-5-carboxamide (28)

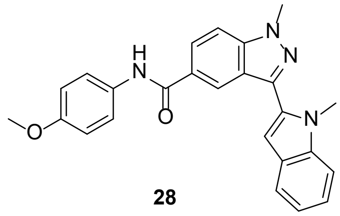

28

Chemical Formula: $C_{25}H_{22}N_4O_2$
Exact Mass: 410.1743

To the solution of 1-methyl-3-(1-methyl-1*H*-indol-2-yl)-1*H*-indazole-5-carboxylic acid (10 mg, 0.032 mmol, 1.0 equiv.), 4-methoxyaniline (5 mg, 0.038 mmol, 1.2 equiv.) and HATU (15 mg, 0.038 mmol, 1.2 equiv.) in anhydrous DMF (0.2 mL) was added DIEA (7 µL, 0.038 mmol, 1.2 equiv.) under nitrogen. The reaction mixture was stirred at room temperature for 16 h and then quenched by the addition of water. The aqueous layer was extracted twice with EtOAc, and the combined organic extracts were washed with brine, dried over anhydrous Na_2SO_4, filtered and concentrated. The residue was purified by column chromatography on silica gel (EtOAc/hexane, 30:70 to 40:60) to afford title compound **28** (8 mg, 60%) as a pale yellow solid; ^{1}H NMR (600 MHz, DMSO-d_6) δ 10.27 (s, 1H), 8.63 (s, 1H), 8.08 (d, *J* = 8.8 Hz, 1H), 7.85 (d, *J* = 8.8 Hz, 1H), 7.69−7.68 (m, 3H), 7.57 (d, *J* = 7.9 Hz, 1H), 7.25 (t, *J* = 7.9 Hz, 1H), 7.20 (s, 1H), 7.12 (t, *J* = 7.9 Hz, 1H), 6.94 (d, *J* = 9.0 Hz, 2H), 4.21 (s, 3H), 4.07 (s, 3H), 3.75 (s, 3H); ^{13}C NMR (150 MHz, DMSO-d_6) δ 165.2, 155.6, 141.5, 138.2, 137.5, 132.3, 131.3, 128.3, 127.5, 126.4, 122.3 (2 ×), 122.2, 121.7, 121.0, 120.5, 119.8, 113.7 (2 ×), 110.2, 110.1, 102.9, 55.2, 36.0, 31.7; HRMS (ESI) *m/z* calcd for $C_{25}H_{23}N_4O_2$ [M + H]$^+$ 411.1815, found 411.1810.

Step 2b: *N*-(4-Methoxy-3-methylphenyl)-1-methyl-3-(1-methyl-1*H*-indol-2-yl)-1*H*-indazole-5-carboxamide (29)

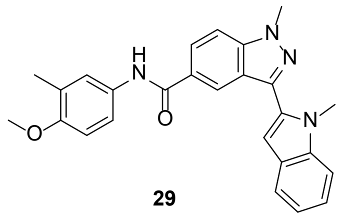

29

Chemical Formula: $C_{26}H_{24}N_4O_2$
Exact Mass: 424.1899

To the solution of 1-methyl-3-(1-methyl-1*H*-indol-2-yl)-1*H*-indazole-5-carboxylic acid (10 mg, 0.032 mmol, 1.0 equiv.), 4-methoxy-3-methylaniline (7 mg, 0.05 mmol, 1.5 equiv.) and HATU (19 mg, 0.05 mmol, 1.5 equiv.) in anhydrous DMF (0.2 mL) was added DIEA (9 µL, 0.05 mmol, 1.5 equiv.) under nitrogen. The reaction mixture was stirred at room temperature for 16 h and then quenched by the addition of water. The aqueous layer was extracted twice with EtOAc, and the combined organic extracts were washed with brine, dried over anhydrous Na_2SO_4, filtered and concentrated. The residue was purified by column chromatography on silica gel (EtOAc/hexane, 20:80 to 30:70) to afford title compound **29** (11 mg, 79%) as a pale yellow solid; ^{1}H NMR (600 MHz, DMSO-d_6) δ 10.20 (s, 1H), 8.63 (s, 1H), 8.08 (d, *J* = 8.8 Hz, 1H), 7.85 (d, *J* = 8.8 Hz, 1H), 7.69 (d, *J* = 7.6 Hz, 1H), 7.57−7.56 (m, 2H), 7.54 (s, 1H), 7.25 (t, *J* = 7.6 Hz, 1H), 7.20 (s, 1H), 7.11 (t, *J* = 7.6 Hz, 1H), 6.93 (d, *J* = 8.7 Hz, 1H), 4.21 (s, 3H), 4.07 (s, 3H), 3.78 (s, 3H), 2.17 (s, 3H); ^{13}C NMR (150 MHz, DMSO-d_6) δ 165.1, 153.7, 141.5, 138.1, 137.5, 131.8, 131.3, 128.4, 127.4, 126.4, 125.3, 123.6, 122.1, 121.7, 120.9, 120.5, 119.8, 119.5, 110.2 (2 ×), 110.0, 102.9, 55.4, 35.9, 31.7, 16.2; HRMS (ESI) *m/z* calcd for $C_{26}H_{25}N_4O_2$ [M + H]$^+$ 425.1972, found 425.1966.

Step 2c: *N*-(3,4-Dimethoxyphenyl)-1-methyl-3-(1-methyl-1*H*-indol-2-yl)-1*H*-indazole-5-carboxamide (30)

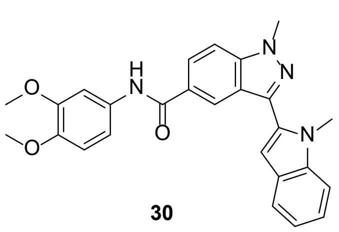

30

Chemical Formula: $C_{26}H_{24}N_4O_3$
Exact Mass: 440.1848

The same procedure for the synthesis of **29** (step 2b) was followed by using 3,4-dimethoxyaniline as the amine. The residue was purified by column chromatography on silica gel (EtOAc/hexane, 30:70 to 35:65) to afford title compound **30** (10 mg, 69%) as a pale yellow solid; ^{1}H NMR (600 MHz, DMSO-d_6) δ 10.25 (s, 1H), 8.63 (s, 1H), 8.09 (d, *J* = 8.7 Hz, 1H), 7.86 (d, *J* = 8.7 Hz, 1H), 7.69 (d, *J* = 7.6 Hz, 1H), 7.57 (d, *J* = 7.6 Hz, 1H), 7.49 (s, 1H), 7.33 (d, *J* = 8.6 Hz, 1H), 7.25 (t, *J* = 7.6 Hz, 1H), 7.19 (s, 1H), 7.12 (t, *J* = 7.6 Hz, 1H), 6.95 (d, *J* = 8.6 Hz, 1H), 4.22 (s, 3H), 4.07 (s, 3H), 3.76 (s, 3H), 3.75 (s, 3H); ^{13}C NMR (150 MHz, DMSO-d_6) δ 165.2, 148.4, 145.1, 141.5, 138.1, 137.5, 132.8, 131.3, 128.4, 127.5, 126.3, 122.2, 121.7, 121.0, 120.5, 119.8, 112.6, 111.8, 110.2, 110.1, 105.8, 102.9, 55.7, 55.4, 36.0, 31.7; HRMS (ESI) *m/z* calcd for $C_{26}H_{25}N_4O_2$ $[M + H]^+$ 441.1921, found 441.1912.

Step 2d: *N*-(3-Fluoro-4-methoxyphenyl)-1-methyl-3-(1-methyl-1*H*-indol-2-yl)-1*H*-indazole-5-carboxamide (31)

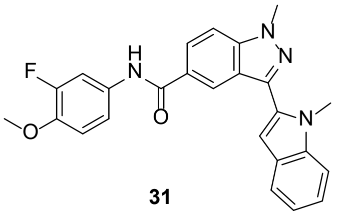

31

Chemical Formula: $C_{25}H_{21}FN_4O_2$
Exact Mass: 428.1649

The same procedure for in the synthesis of **29** (step 2b) was followed by using 3-fluoro-4-methoxyaniline as the amine. The residue was purified by column chromatography on silica gel (EtOAc/hexane, 30:70 to 35:65) to afford title compound **31** (10 mg, 69%) as a pale yellow solid; ^{1}H NMR (600 MHz, DMSO-d_6) δ 10.40 (s, 1H), 8.63 (s, 1H), 8.07 (d, *J* = 8.8 Hz, 1H), 7.86 (d, *J* = 8.8 Hz, 1H), 7.77 (dd, *J* = 13.7, 2.1 Hz, 1H), 7.69 (d, *J* = 7.9 Hz, 1H), 7.57 (d, *J* = 7.9 Hz, 1H), 7.51 (d, *J* = 8.8 Hz, 1H), 7.25 (t, *J* = 7.9 Hz, 1H), 7.19 (s, 1H), 7.17 (d, *J* = 8.8 Hz, 1H), 7.12 (t, *J* = 7.9 Hz, 1H), 4.21 (s, 3H), 4.07 (s, 3H), 3.83 (s, 3H); ^{13}C NMR (150 MHz, DMSO-d_6) δ 165.4, 150.8 (d, *J* = 242 Hz), 143.2 (d, *J* = 11 Hz), 141.6, 138.2, 137.6, 132.7 (d, *J* = 10 Hz), 131.2, 128.0, 127.4, 126.3, 122.2, 121.7, 121.2, 120.5, 119.8, 116.5 (d, *J* = 3 Hz), 113.8, 110.2, 110.1, 108.9 (d, *J* = 22 Hz), 103.0, 56.2, 36.0, 31.7; HRMS (ESI) *m/z* calcd for $C_{25}H_{22}FN_4O_2$ $[M + H]^+$ 429.1721, found 429.1717.

Step 2e: *N*-(3-Chloro-4-methoxyphenyl)-1-methyl-3-(1-methyl-1*H*-indol-2-yl)-1*H*-indazole-5-carboxamide (32)

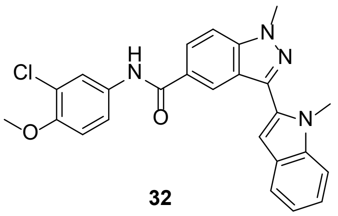

32

Chemical Formula: $C_{25}H_{21}ClN_4O_2$
Exact Mass: 444.1353

The same procedure for the synthesis of **29** (step 2b) was followed by using 3-chloro-4-methoxyaniline as the amine. The residue was purified by column chromatography on silica gel (EtOAc/hexane, 30:70 to 35:65) to afford title compound **32** (12 mg, 84%) as a pale yellow solid; ^{1}H NMR (600 MHz, DMSO-d_6) δ 10.38 (s, 1H), 8.64 (s, 1H), 8.08 (d, *J* = 8.8 Hz, 1H), 7.95 (d, *J* = 2.3 Hz, 1H), 7.86 (d, *J* = 8.8 Hz, 1H), 7.71−7.69 (m, 2H), 7.57 (d, *J* = 7.5 Hz, 1H), 7.26 (t, *J* = 7.5 Hz, 1H), 7.19 (s, 1H), 7.17 (d, *J* = 8.9 Hz, 1H), 7.12 (t, *J* = 7.5 Hz, 1H), 4.21 (s, 3H), 4.07 (s, 3H), 3.85 (s, 3H); ^{13}C NMR (150 MHz, DMSO-d_6) δ 165.4, 150.8, 141.6, 138.2, 137.6, 132.9, 131.2, 127.9, 127.4, 126.3, 122.2 (2 ×), 121.7, 121.2, 120.5, 120.4, 120.4, 119.8, 112.8, 110.2, 110.1, 102.9, 56.2, 36.0, 31.7; HRMS (ESI) *m/z* calcd for $C_{25}H_{22}ClN_4O_2$ $[M + H]^+$ 445.1425, found 445.1414.

Step 2f: ***N*-(4-Methoxy-2-methylphenyl)-1-methyl-3-(1-methyl-1*H*-indol-2-yl)-1*H*-indazole-5-carboxamide (33)**

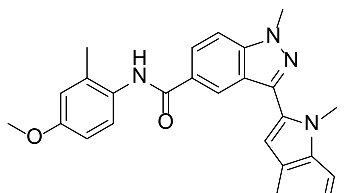

33

Chemical Formula: $C_{26}H_{24}N_4O_2$
Exact Mass: 424.1899

The same procedure for the synthesis of **29** (step 2b) was followed by using 4-methoxy-2-methylaniline as the amine. The residue was purified by column chromatography on silica gel (EtOAc/hexane, 30:70 to 35:65) to afford title compound **33** (10 mg, 72%) as a pale yellow solid; ^{1}H NMR (600 MHz, DMSO-d_6) δ 9.98 (s, 1H), 8.69 (s, 1H), 8.09 (d, J = 8.8 Hz, 1H), 7.85 (d, J = 8.8 Hz, 1H), 7.67 (d, J = 7.8 Hz, 1H), 7.57 (d, J = 7.8 Hz, 1H), 7.26−7.20 (m, 3H), 7.11 (t, J = 7.8 Hz, 1H), 6.87 (d, J = 2.6 Hz, 1H), 6.80 (dd, J = 8.6, 2.6 Hz, 1H), 4.21 (s, 3H), 4.08 (s, 3H), 3.76 (s, 3H), 2.21 (s, 3H); ^{13}C NMR (150 MHz, DMSO-d_6) δ 165.4, 157.4, 141.6, 138.2, 137.5, 135.8, 131.3, 129.4, 128.3, 127.9, 127.4, 126.4, 122.2, 121.7, 120.9, 120.4, 119.8, 115.4, 111.3, 110.2, 110.0, 102.9, 55.2, 36.0, 31.7, 18.2; HRMS (ESI) *m/z* calcd for $C_{26}H_{25}N_4O_2$ [M + H]$^+$ 425.1972, found 425.1965.

Step 2g: ***N*-(2,4-Dimethoxyphenyl)-1-methyl-3-(1-methyl-1*H*-indol-2-yl)-1*H*-indazole-5-carboxamide (34)**

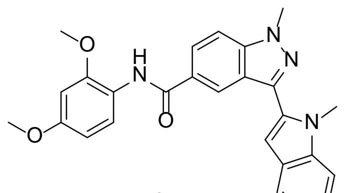

34

Chemical Formula: $C_{26}H_{24}N_4O_3$
Exact Mass: 440.1848

The same procedure for the synthesis of **29** (step 2b) was followed by using 2,4-dimethoxyaniline as the amine. The residue was purified by column chromatography on silica gel (EtOAc/hexane, 30:70 to 35:65) to afford title compound **34** (11 mg, 76%) as a pale yellow solid; ^{1}H NMR (600 MHz, DMSO-d_6) δ 9.67 (s, 1H), 8.66 (s, 1H), 8.06 (d, J = 8.8 Hz, 1H), 7.83 (d, J = 8.8 Hz, 1H), 7.67 (d, J = 7.8 Hz, 1H), 7.57 (d, J = 7.8 Hz, 1H), 7.43 (d, J = 8.6 Hz, 1H), 7.26−7.23 (m, 2H), 7.11 (t, J = 7.8 Hz, 1H), 6.67 (d, J = 2.5 Hz, 1H), 6.56 (dd, J = 8.6, 2.5 Hz, 1H), 4.21 (s, 3H), 4.08 (s, 3H), 3.79 (s, 3H), 3.79 (s, 3H); ^{13}C NMR (150 MHz, DMSO-d_6) δ 165.4, 158.1, 154.0, 141.6, 138.2, 137.6, 131.3, 128.0, 127.5, 127.3, 126.4, 122.2, 121.7, 121.0, 120.4, 119.8 (2 ×), 110.2, 110.0, 104.2, 102.9, 98.9, 55.7, 55.4, 36.0, 31.7; HRMS (ESI) *m/z* calcd for $C_{26}H_{25}N_4O_3$ [M + H]$^+$ 441.1921, found 441.1909.

Step 2h: ***N*-(2-Fluoro-4-methoxyphenyl)-1-methyl-3-(1-methyl-1*H*-indol-2-yl)-1*H*-indazole-5-carboxamide (35)**

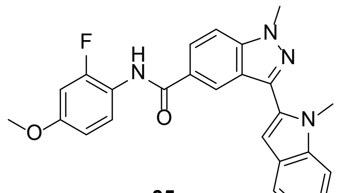

35

Chemical Formula: $C_{25}H_{21}FN_4O_2$
Exact Mass: 428.1649

The same procedure for the synthesis of **29** (step 2b) was followed by using 2-fluoro-4-methoxyaniline as the amine. The residue was purified by column chromatography on silica gel (EtOAc/hexane, 30:70 to 35:65) to afford title compound **35** (10.4 mg, 74%) as a pale yellow solid; ^{1}H NMR (600 MHz, DMSO-d_6) δ 10.15 (s, 1H), 8.69 (s, 1H), 8.09 (d, J = 8.8 Hz, 1H), 7.85 (d, J = 8.8 Hz, 1H), 7.68 (d, J = 7.8 Hz, 1H), 7.57 (d, J = 7.8 Hz, 1H), 7.43 (t, J = 8.9 Hz, 1H), 7.25 (t, J = 7.8 Hz, 1H), 7.21 (s, 1H), 7.12 (t, J = 7.8 Hz, 1H), 6.95 (dd, J= 12.2, 2.3 Hz, 1H), 6.82 (dd, J = 8.9, 2.3 Hz, 1H), 4.21 (s, 3H), 4.08 (s, 3H), 3.79 (s, 3H); ^{13}C NMR (150 MHz, DMSO-d_6) δ 165.5, 158.3 (d, J = 10 Hz), 157.0 (d, J = 246 Hz), 141.6, 138.2, 137.6, 131.2, 128.7, 127.4, 127.3, 126.5, 122.2, 121.7, 121.2, 120.4, 119.8, 118.3 (d, J = 13 Hz), 110.2, 110.1, 109.9, 102.9, 102.0 (d, J = 24 Hz), 55.7, 36.0, 31.7; HRMS (ESI) *m/z* calcd for $C_{25}H_{22}FN_4O_2$ [M + H]$^+$ 429.1721, found 429.1711.

Step 2i: *N*-(2-Chloro-4-methoxyphenyl)-1-methyl-3-(1-methyl-1*H*-indol-2-yl)-1*H*-indazole-5-carboxamide (36)

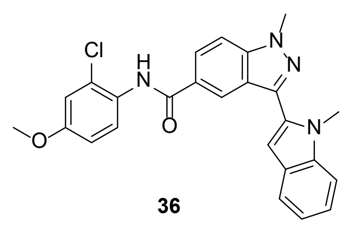

36

Chemical Formula: $C_{25}H_{21}ClN_4O_2$
Exact Mass: 444.1353

The same procedure for the synthesis of **29** (step 2b) was followed by using 2-chloro-4-methoxyaniline as the amine. The residue was purified by reverse-phase column chromatography on C-18 (CH_3CN/water, 5:95 to 100:0) to afford title compound **36** (8 mg, 56%) as a yellow solid; ^{1}H NMR (600 MHz, DMSO-d_6) δ 10.17 (s, 1H), 8.71 (s, 1H), 8.09 (d, *J* = 8.8 Hz, 1H), 7.86 (d, *J* = 8.8 Hz, 1H), 7.67 (d, *J* = 7.8 Hz, 1H), 7.57 (d, *J* = 7.8 Hz, 1H), 7.44 (d, *J* = 8.7 Hz, 1H), 7.25 (t, *J* = 7.8 Hz, 1H), 7.21 (s, 1H), 7.16 (t, *J* = 2.0 Hz, 1H), 7.12 (t, *J* = 7.8 Hz, 1H), 6.99 (dd, *J* = 8.7, 2.0 Hz, 1H), 4.21 (s, 3H), 4.08 (s, 3H), 3.81 (s, 3H); ^{13}C NMR (150 MHz, DMSO-d_6) δ 165.7, 158.1, 141.7, 138.2, 137.6, 131.2 (2 ×), 130.1, 128.0, 127.4, 127.4, 126.4, 122.2, 121.7, 121.2, 120.4, 119.8, 114.5, 113.5, 110.2, 110.1, 102.9, 55.8, 36.0, 31.7; HRMS (ESI) *m/z* calcd for $C_{25}H_{22}ClN_4O_2$ $[M + H]^+$ 445.1425, found 445.1427.

4q) ^{1}H and ^{13}C NMR spectra of CDD-1102 (9), CDD-1349 (15), and CDD-1302 (20)

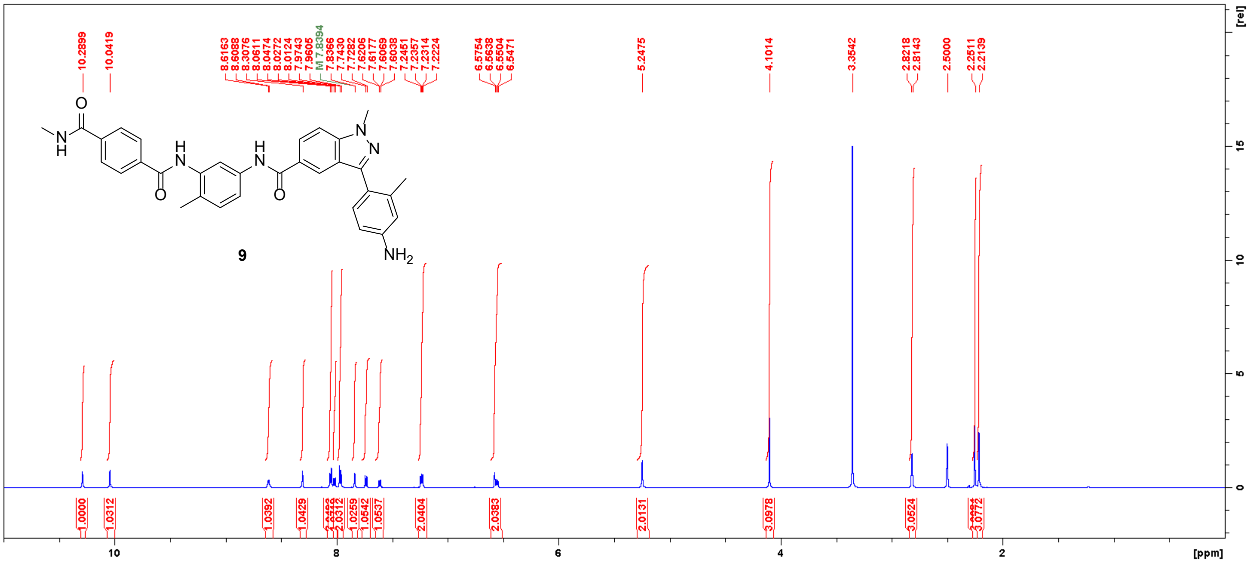

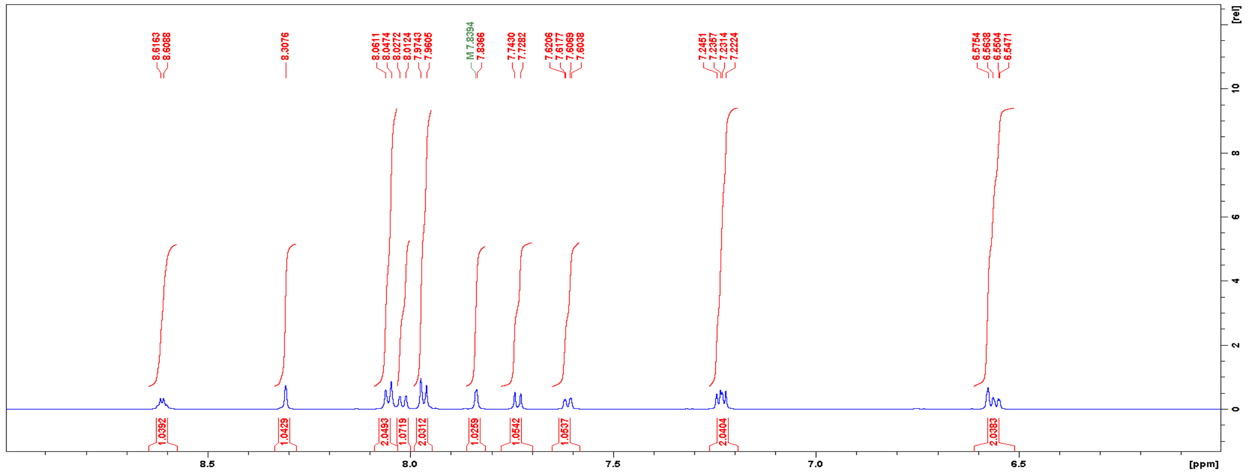

^{1}H NMR (DMSO-d_6, 600 MHz) and zoom-in (7–9 ppm) spectra of CDD-1102 (**9**)

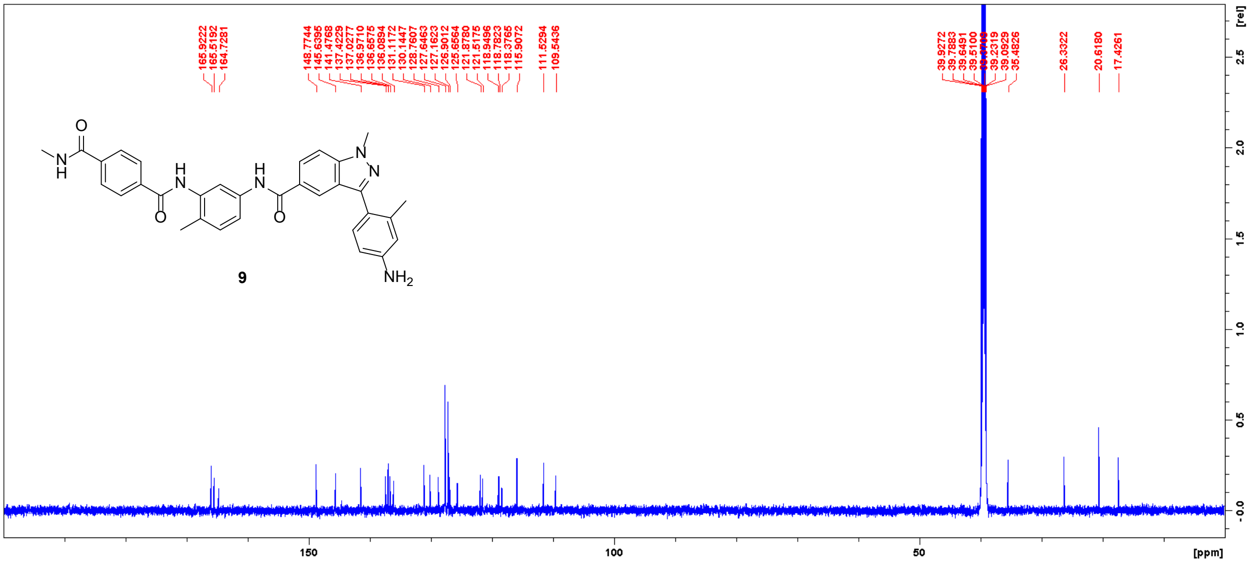

^{13}C NMR (DMSO-d_6, 150 MHz) spectrum of CDD-1102 (**9**)

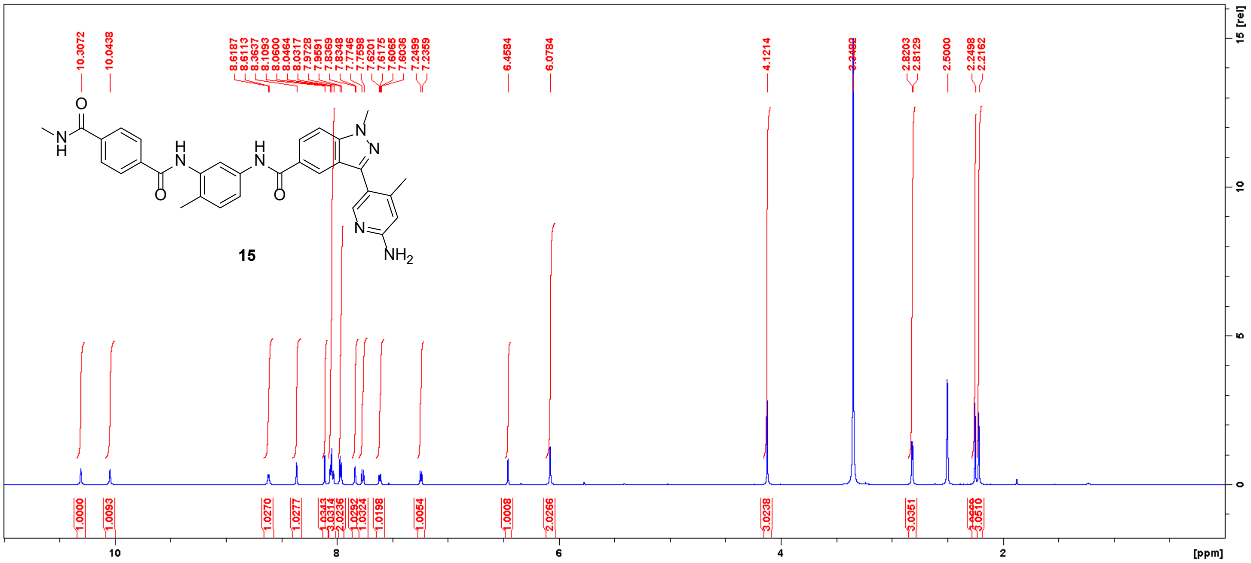

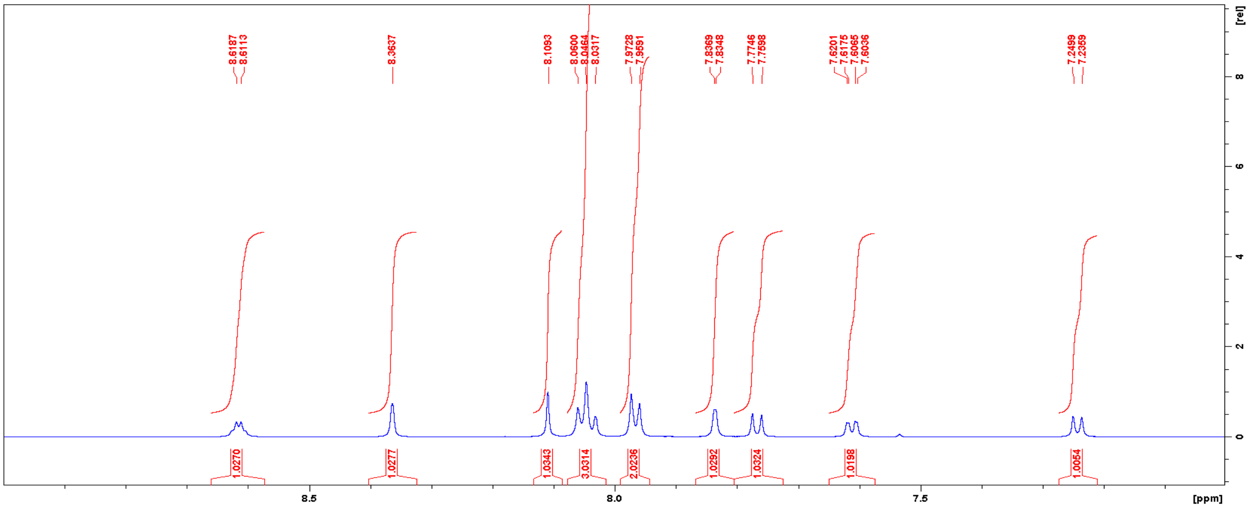

^{1}H NMR (DMSO-d_6, 600 MHz) and zoom-in (7–9 ppm) spectra of CDD-1349 (**15**)

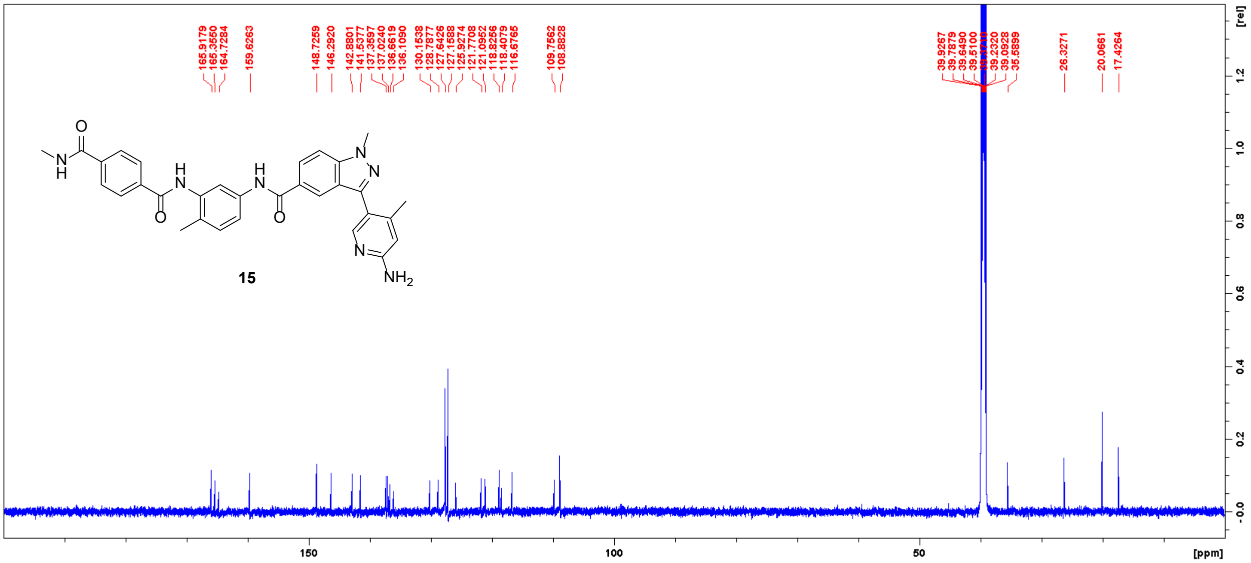

^{13}C NMR (DMSO-d_6, 150 MHz) spectrum of CDD-1349 (**15**)

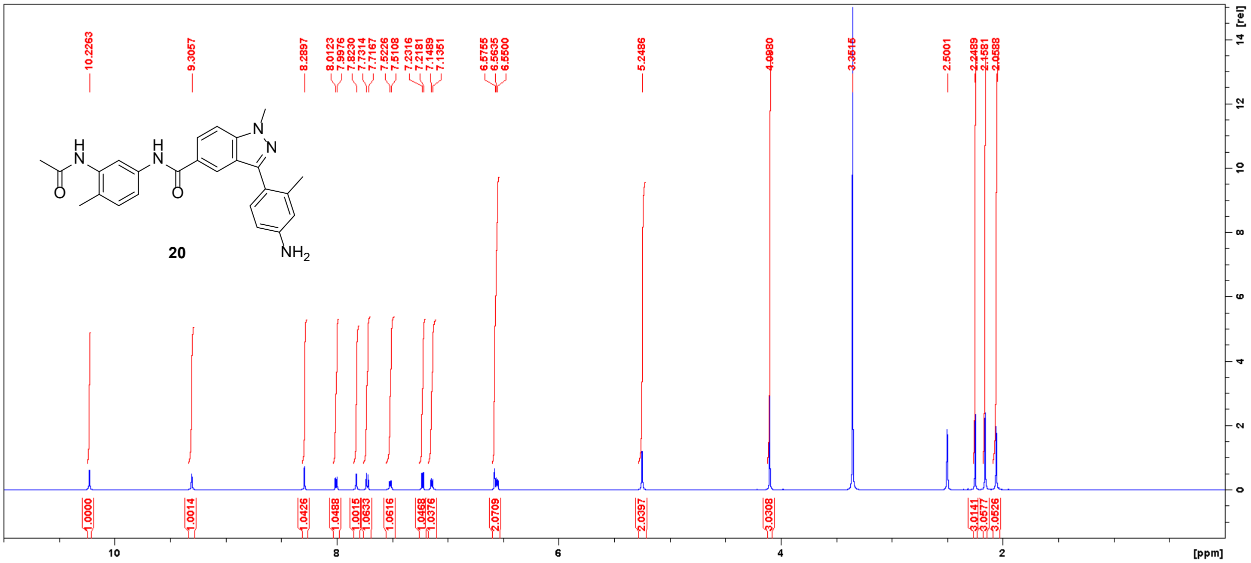

^{1}H NMR (DMSO-d_6, 600 MHz) spectrum of CDD-1302 (**20**)

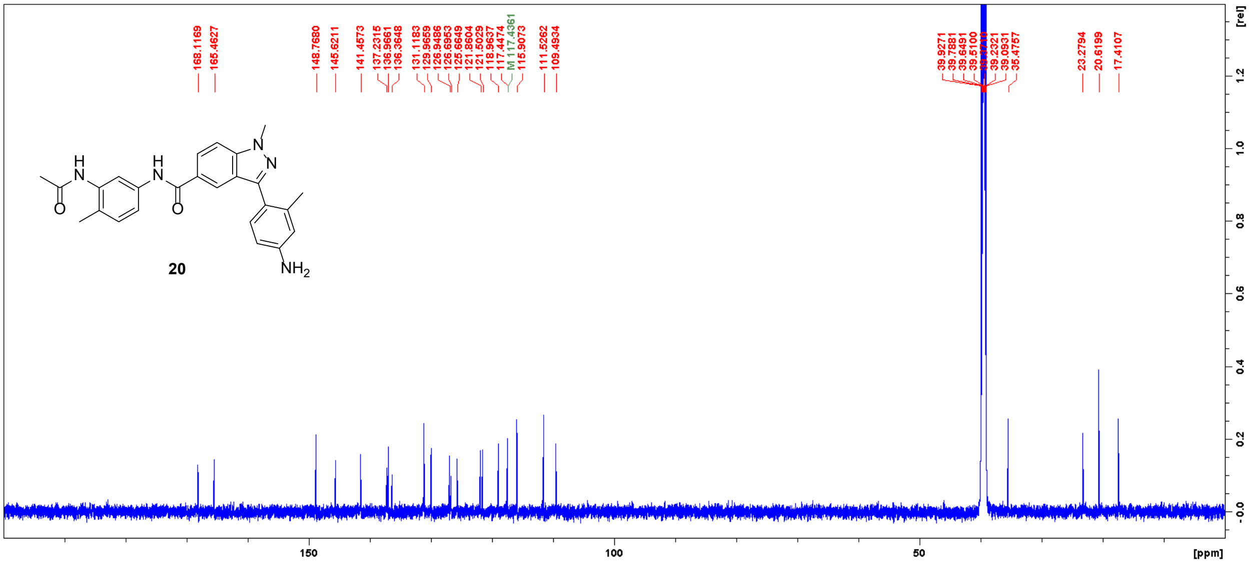

^{13}C NMR (DMSO-d_6, 150 MHz) spectrum of CDD-1302 (**20**)

5. Reference

1. M. M. Matzuk *et al.*, Small-molecule inhibition of BRDT for male contraception. *Cell* **150**, 673-684 (2012).
2. T. G. Battye, L. Kontogiannis, O. Johnson, H. R. Powell, A. G. Leslie, iMOSFLM: a new graphical interface for diffraction-image processing with MOSFLM. *Acta Crystallogr D Biol Crystallogr* **67**, 271-281 (2011).
3. P. R. Evans, An introduction to data reduction: space-group determination, scaling and intensity statistics. *Acta Crystallogr D Biol Crystallogr* **67**, 282-292 (2011).
4. A. J. McCoy *et al.*, Phaser crystallographic software. *J Appl Crystallogr* **40**, 658-674 (2007).
5. P. V. Afonine *et al.*, Towards automated crystallographic structure refinement with phenix.refine. *Acta Crystallogr D Biol Crystallogr* **68**, 352-367 (2012).
6. P. Emsley, K. Cowtan, Coot: model-building tools for molecular graphics. *Acta Crystallogr D Biol Crystallogr* **60**, 2126-2132 (2004).
7. W. DeLano, The PyMOL Molecular Graphics System. *San Carlos, CA: DeLano Scientific* (2002).
8. Schrödinger_Release_2019-1, Schrödinger LLC. *New York, NY* (2019).

6. Supplementary Figures and Tables

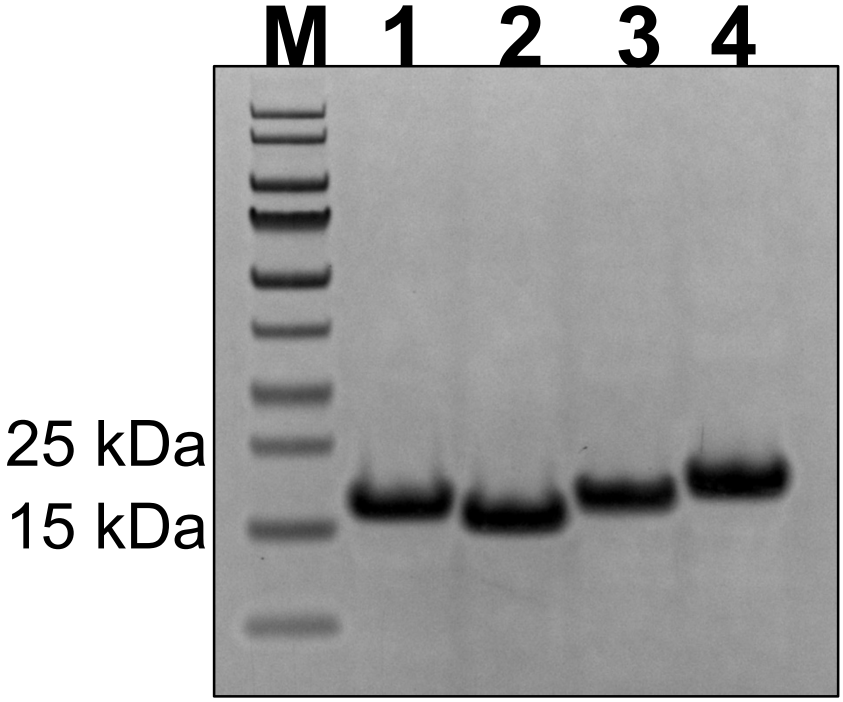

Fig. S1. The recombinant bromodomain proteins purified for the DEC-Tec selection were pure. Five micrograms His-BRDT-BD1 (lane 1), His-BRDT-BD2 (lane 2), His-BRD4-BD1 (lane 3), and His-BRD4-BD2 (lane 4) were loaded onto NuPAGE 4 to 12%, Bis-Tris gradient gel (Invitrogen) and stained with Instant blue protein stain.

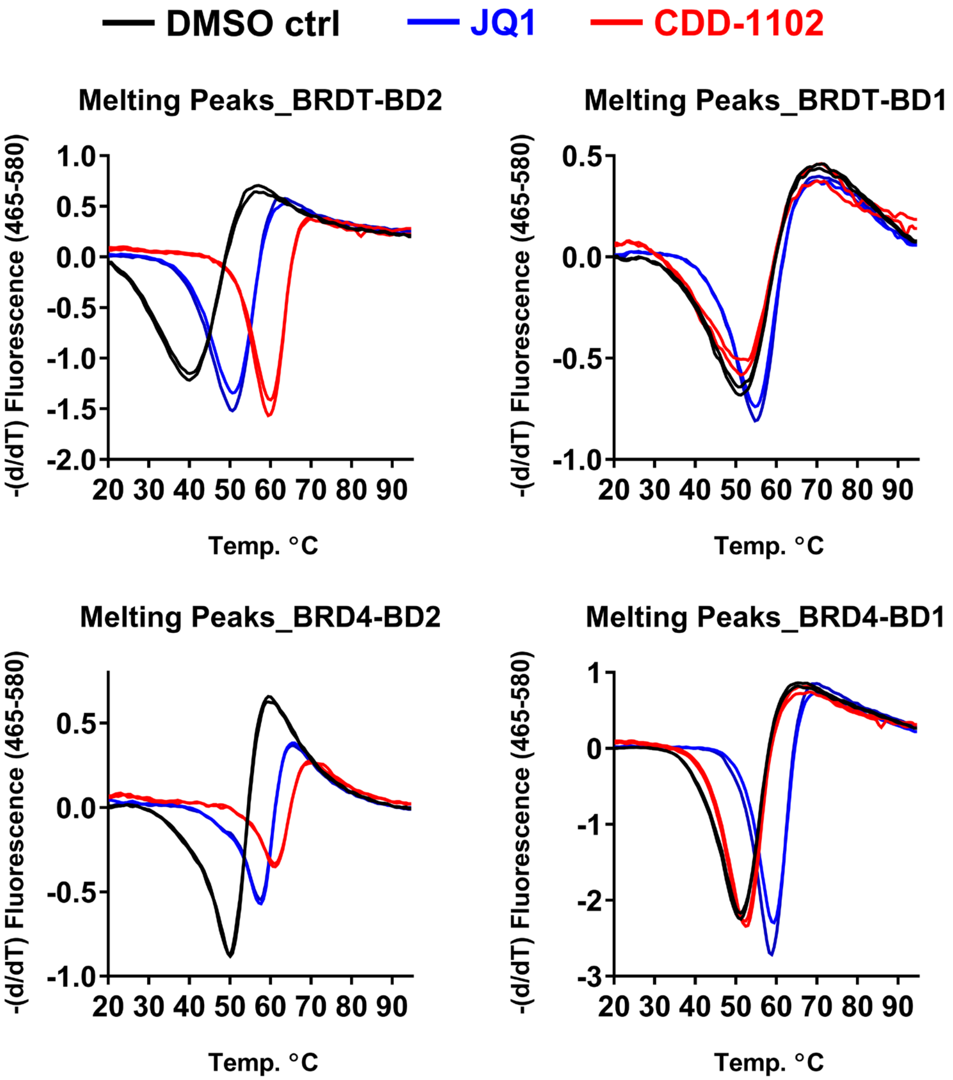

Compound	Concentration	Protein	ΔTm ± STDEV (°C)
CDD-1102	25 μM	BRDT-BD1	0.13 ± 0.20
		BRDT-BD2	19.47 ± 0.16
		BRD4-BD1	1.09 ± 0.12
		BRD4-BD2	11.10 ± 0.04
JQ1	25 μM	BRDT-BD1	3.48 ± 0.14
		BRDT-BD2	10.34 ± 0.16
		BRD4-BD1	7.84 ± 0.33
		BRD4-BD2	7.43 ± 0.08

Fig. S2. CDD-1102 significantly stabilized the 2nd but not the 1st bromodomain of BRDT and BRD4 in the protein thermal shift stability assay. (+)-JQ1 at the same concentration was run in parallel as a positive control. Data analysis and protein melting temperature (Tm) calculation were run on a Roche Lightcycler 480 real-time PCR instrument (n = 2).

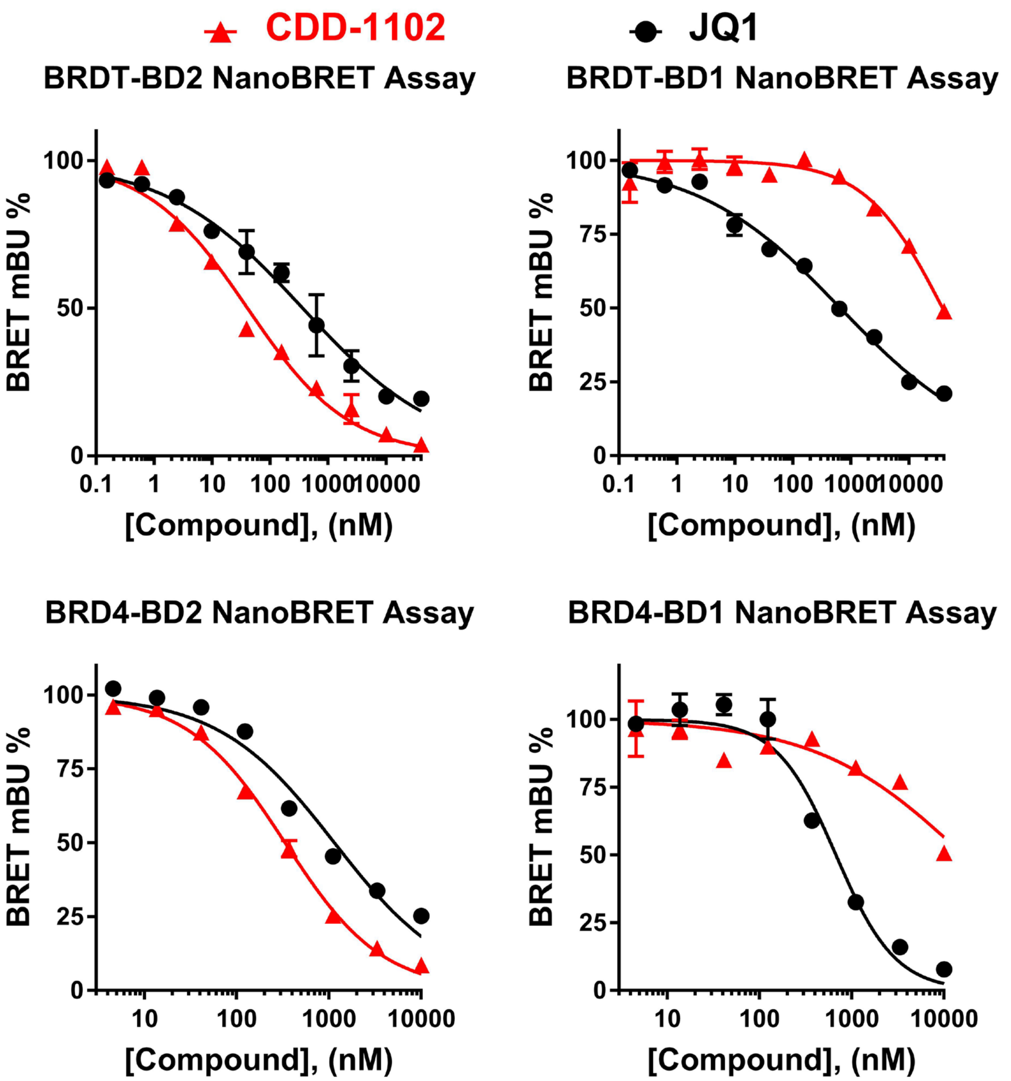

IC$_{50}$, nM	BRDT-BD2	BRDT-BD1	BRD4-BD2	BRD4-BD1
CDD-1102	40	37,600	335	16,400
JQ1	360	650	1,150	675

Fig. S3. CDD-1102 selectively inhibited the second bromodomain of BRDT and BRD4 in NanoBRET target engagement assay. NanoBRET tracer competition by CDD-1102 was performed in transiently transfected HEK293 cells expressing NanoLuc-BRDT-BD2, NanoLuc-BRDT-BD1, NanoLuc-BRD4-BD2, or NanoLuc-BRD4-BD1 fusion protein. (+)-JQ1 was measured in parallel as a positive control. GraphPad Prism software was used to generate inhibition fitting curves and to determine IC$_{50}$ values.

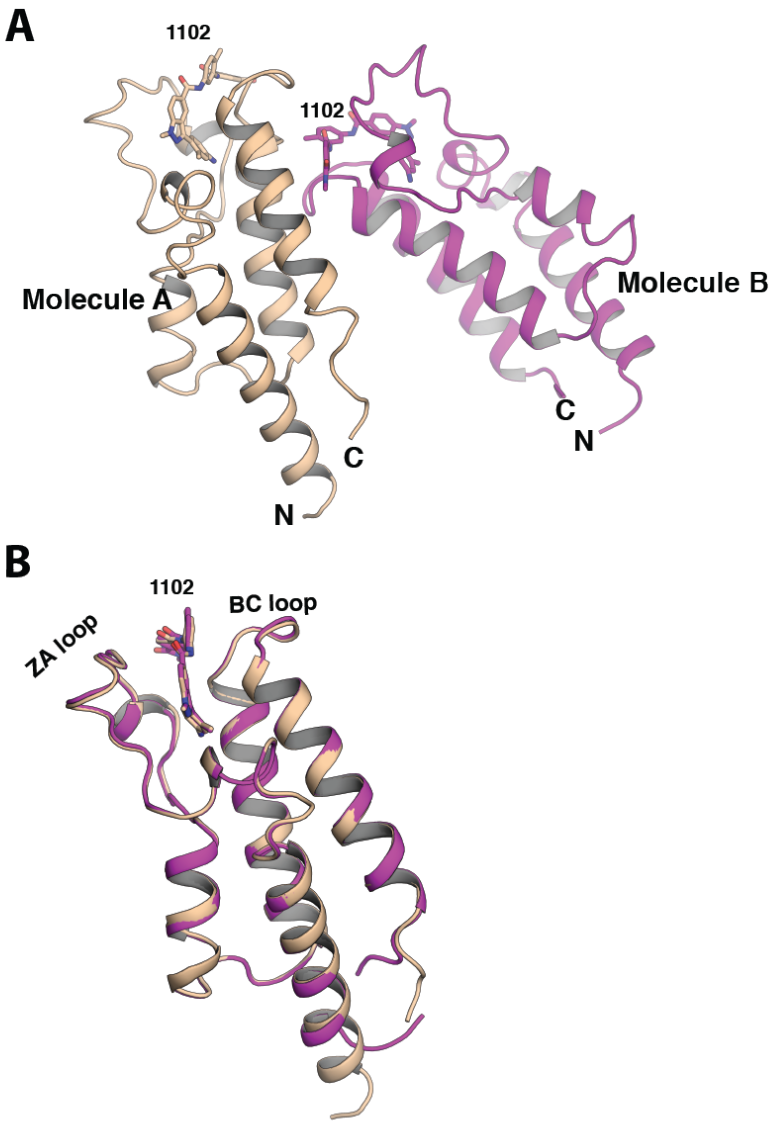

Fig. S4. Two molecules captured in the BRDT-BD2/CDD-1102 complex crystal.

A. Two BD2 molecule are captured in asymmetric unit with one CDD-1102 bound to each molecule. The molecule A is colored in wheat and B in magenta. CDD-1102 shown in sticks. N and C terminus are labelled.

B. Superimposition of two BD2/CDD-1102 complexes. Two molecules are nearly identical (showing an RMSD of 0.25 Å between 97 CA atoms).

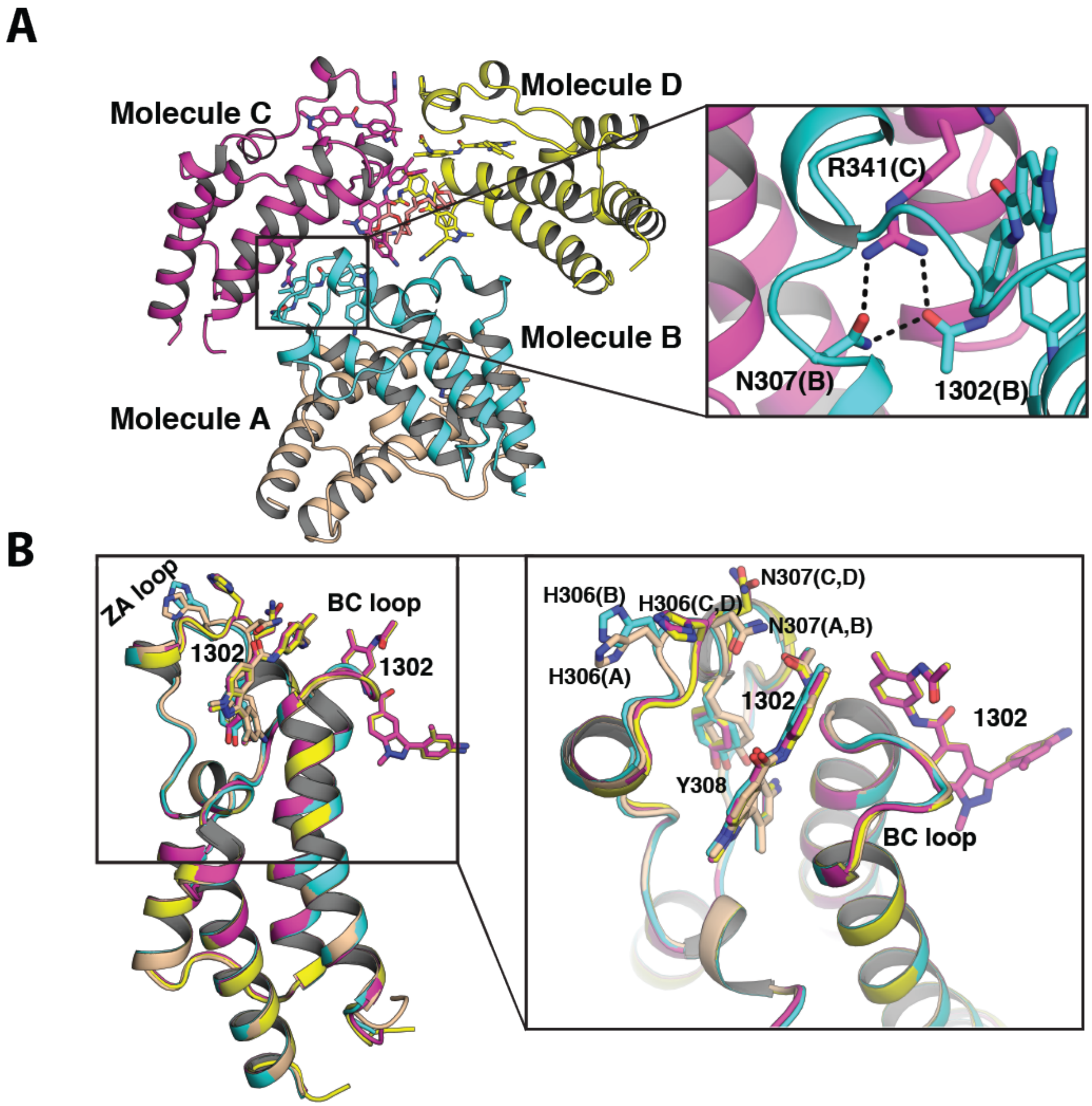

Fig. S5. Four molecules captured in the BRDT-BD2/CDD-1302 complex crystal.

A. Four BRDT-BD2/CDD-1302 complexes captured in asymmetric unit. The molecules A and B show one CDD-1302 bound at the KAc binding pocket whereas molecules C and D show additional CDD-1302 bound at the unseen site near the BC loop. Molecule A is colored in wheat, B in cyan, C in magenta and D in yellow. CDD-1302 and Jeffamine ED-2000 are shown in sticks. A zoom in view in right shows a unique crystal contact between molecule B and C that causes a different conformation of the ZA loop segment in molecules A and B.

B. Alignment of four BRDT-BD2/CDD-1302 complexes captured in the asymmetric unit. Four molecules show nearly identical structures except for their ZA loops. Molecules C and D show different conformations at the ZA loop. A zoom-in view of the ZA loop with key residues showing in sticks.

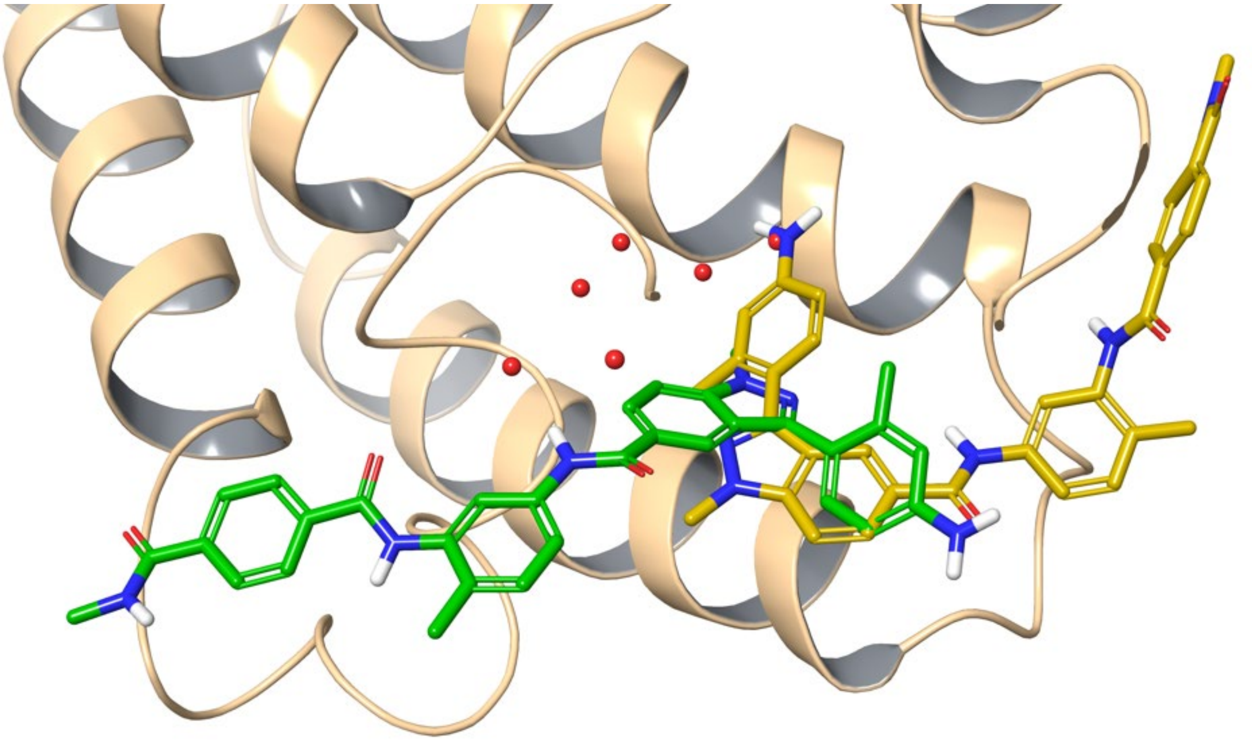

Fig. S6. The modeled (green sticks) and co-crystal (yellow sticks) poses of CDD-1102. The modeled pose was obtained through an extensive small molecule docking study using a homology model of human BRDT-BD2 containing the ZA channel waters of bromodomains (shown as red dots), as these water molecules frequently play a role in binding inside the KAc pocket. To our surprise, the aniline -NH_2 group of both CDD-1102 and CDD-1302 successfully replaced a ZA channel water, which allowed the ligands to penetrate into the KAc pocket very deeply. This water molecule usually bridges the conserved Y308 (BRDT-BD2 numbering) and the KAc of the acetylated histone or small molecule BET inhibitors. The computational modeling was performed using the Schrodinger Suite of Programs (8).

Table S1. Bromodomain constructs expressed and purified for the DEC-Tec selection and assay validation. All constructs were subcloned into bacterial vectors pET15b or pET28b (addgene) and were purified on Talon cobalt resins. The final buffer was exchanged to 50 mM HEPES pH 7.5, 150 mM NaCl, 1 mM TCEP and 10% glycerol on PD-10 columns (Amersham Biosciences).

Name	Expressed Sequence
**BRDT-BD1**	MGSSHHHHHHSSGLVPRGSHMNTKKNGRLTNQLQYLQKVVLKDLWKHSFS WPFQRPVDAVKLQLPDYYTIIKNPMDLNTIKKRLENKYYAKASECIEDFNTMFS NCYLYNKPGDDIVLMA QALEKLFMQKLSQMPQEE
**BRDT-BD2**	MGSSHHHHHHSSGLVPRGSHMASVTEQLRHCSEILKEMLAKKHFSYAWPFY NPVDVNALGLHNYYDVVKNPMDLGTIKEKMDNQEYKDAYKFAADVRLMFMN CYKYNPPDHEVVTMARMLQDVFETHFSKIPIEP
**BRD4-BD1**	MGSSHHHHHHSSGLVPRGSHMNPPPPETSNPNKPKRQTNQLQYLLRVVLKT LWKHQFAWPFQQPVDAVKLNLPDYYKIIKTPMDMGTIKKRLENNYYWNAQECI QDFNTMFTNCYIYNKPGDDIVLMAEALEKLFLQKINELPTEE
**BRD4-BD2**	MHHHHHHSSGVDLGTENLYFQSNAKDVPDSQQHPAPEKSSKVSEQLKCCSG ILKEMFAKKHAAYAWPFYKPVDVEALGLHDYCDIIKHPMDMSTIKSKLEAREYR DAQEFGADVRLMFSNCYKYNPPDHEVVAMARKLQDVFEMRFAKMPDE

Table S2. Data collection and refinement statistics.

Complex	BRDT-BD2/CDD-1102	BRDT-BD2/CDD-1302
PDB ID code	7L9A	7L99
Data collection		
Space group	$P\ 3_2\ 2\ 1$	$P\ 1\ 2_1\ 1$
Cell Dimensions		
a, b, c (Å)	56.47, 56.47, 191.90	47.23, 61.97, 99.18
α, β, γ (°)	90, 90, 120	90, 97.28, 90
Resolution (Å)	47.39−2.27 (2.35−2.27)[a]	44.55−1.90 (1.96−1.90)[a]
R_{merge} (%)	4.99 (60.81)	8.79 (108.30)
I/ σ (I)	10.90 (1.00)	8.24 (1.65)
$CC_{1/2}$	0.99 (0.67)	0.98 (0.20)
Completeness (%)	99.73 (99.47)	98.17 (94.68)
Multiplicity	2.0 (2.0)	2.0 (1.8)
Refinement		
No. of unique reflections	17,207 (1,684)	44,413 (4,301)
R_{work}/R_{free}[b]	23.46/27.13 (32.01/35.15)	17.82/19.44 (28.46/26.83)
No. of atoms		
Protein	1,854	3,641
Ligand	82	233
Water	42	325
R.m.s.[c] *deviations*		
Bond length (Å)	0.009	0.007
Bond angles (°)	0.99	1.00
Ramachandran plot (%)		
Favored region	96.05	99.08
Allowed region	3.07	0.69
Outliers	0.88	0.23

[a] Highest resolution shell is shown in parenthesis.

[b] 10% of the observed intensities was excluded from refinement for cross validation purposes.

[c] *R.m.s.* means root mean square.
